# Structural basis of step II spliced leader RNA trans-splicing in trypanosomatid parasites

**DOI:** 10.1038/s41467-026-77480-6

**Published:** 2026-09-23

**Authors:** Théo Nadenoen, Franco Agustín Biglione, Marylène Vandevenne, Arnaud Vanden Broeck

**Affiliations:** 1 Laboratory of RNA Structural Biology and Biochemistry, Centre for Protein Engineering, InBioS Research Unit, University of Liège, Liège, Belgium; 2 Robotein Facility, Centre for Protein Engineering, InBioS Research Unit, University of Liège, Liège, Belgium; 3 Laboratory of Protein and Nucleic Acid Chemistry, The Rockefeller University, New York, NY USA

**Keywords:** Cryoelectron microscopy, RNA, RNA splicing

## Abstract

Messenger RNA (mRNA) synthesis is fundamental to eukaryotic gene expression. In kinetoplastid parasites, including the human pathogens *Trypanosoma* and *Leishmania*, mRNAs are produced through a mechanism called Spliced Leader (SL) RNA trans-splicing. In this process, a short SL exon from a small noncoding RNA is joined to the 5’ end of every mRNA, ensuring transcript stability and translation. Despite decades of study, the structural and mechanistic basis of SL trans-splicing remains elusive. Here, we report cryogenic electron microscopy structures of step II SL trans-splicing machineries from *Leishmania tarentolae*. These structures reveal the molecular mechanism of SL exon ligation and uncover lineage-specific adaptations that remodel the trypanosomatid trans-spliceosome for SL trans-splicing while preserving fundamental spliceosomal chemistry. Altogether, our results establish a mechanistic framework for SL trans-splicing and illuminate the evolutionary diversification of RNA processing in deeply diverged eukaryotes.

## Abstract (web summary)

Trypanosomatid parasites rely on Spliced Leader (SL) RNA trans-splicing to produce every mature mRNA. Here, the authors present cryo-EM structures of the trans spliceosome during SL exon ligation, providing structural insight into this essential RNA processing pathway.

## Introduction

Trypanosomatids, a group of early-diverging eukaryotes, include several parasitic species responsible for major neglected tropical diseases. Among them, *Trypanosoma brucei*, *Trypanosoma cruzi*, and *Leishmania* species, cause sleeping sickness, Chagas disease, and leishmaniasis, respectively, collectively threatening millions of people worldwide^1–3^. These parasites impose a significant burden on global health, particularly in low-income regions, where treatment options remain limited and emerging drug resistance poses an increasing challenge. Understanding the molecular mechanisms that underpin their biology is therefore crucial for developing new therapeutic strategies^4^.

Unlike most eukaryotes, protein-coding genes in trypanosomatids are arranged in long polycistronic transcription units that are constitutively transcribed, as individual promoters controlling their expression are largely absent. Consequently, gene expression regulation occurs predominantly at the post-transcriptional level. As a result, individual mRNAs are generated from long precursor transcripts through coupled RNA processing reactions involving spliced leader (SL) trans-splicing and polyadenylation. SL trans-splicing is an essential mechanism in which a short capped SL exon is transferred from the SL RNA to the 5’ end of each pre-mRNA, converting polycistronic transcripts into mature monocistronic mRNAs^5,6^. Mechanistically, SL trans-splicing is evolutionary related to intron-removal cis-splicing as it proceeds through the same two sequential transesterification reactions–branching and SL exon ligation–catalyzed by the trans-spliceosome, a large RNA–protein complex that orchestrates the recognition and ligation of RNA substrates^7^. While virtually every pre-mRNA in trypanosomatids undergoes SL trans-splicing, conventional cis-splicing is rare and has been described for only two genes in *Trypanosoma* and most *Leishmania* species, the poly(A) polymerase PAP1 and the DEAD-box helicase DBP2B, each containing a single intron^8,9^.

While the trans-spliceosome shares core components with the canonical spliceosome, including U2, U4/U6, and U5 snRNPs, it also involves the SL snRNP, a dedicated complex unique to SL trans-splicing. In trypanosomatids, the U-rich snRNAs that form the core of these snRNPs are markedly divergent from their counterparts in other eukaryotes, both in sequence and structural features^10^. The canonical RNA-RNA interaction motifs that mediate base pairing between snRNAs and their targets on the SL and pre-mRNA substrates are often highly degenerate, obscuring the rules of molecular recognition during trans-splicing and hindering a complete understanding of the reaction mechanism. For instance, trypanosomatids U2 snRNA lacks the motif that typically recognizes the branch point sequence, and both U2 and U4 snRNAs contain degenerate Sm sites that are bound by trypanosomatid-specific Sm-like (SSm) proteins^11–13^. Crucially, how the SL RNA substrate is recognized, recruited, and stabilized by U2, U5 and U6 snRNAs within the catalytic core of the trans-spliceosome remains unclear. At the protein level, many spliceosomal factors exhibit limited sequence identity to their opisthokont orthologs, and several appear to be lineage-specific innovations^14,15^. This divergence has hindered efforts to comprehensively define the composition of spliceosomal subunits in trypanosomatids using genome annotation alone^16^. To date, only a partial repertoire of protein factors has been identified, primarily through mass spectrometry-based studies^14,17–20^. Despite its essential role, the molecular mechanism of SL trans-splicing in trypanosomatid parasites, as well as a comprehensive inventory of the factors involved, remain poorly defined owing to the absence of structural information and a knowledge base limited to biochemical studies (reviewed in refs. ^6,7,21^).

In this work, we present high-resolution cryogenic electron microscopy (cryo-EM) structures of the trypanosomatid trans-spliceosome captured at two states flanking the second step of SL trans-splicing, together with biochemical and AI-based protein-protein interaction analyses. These structures reveal how the trans-spliceosome accommodates the SL RNA, orchestrates 3’ splice site recognition, and catalyzes SL exon ligation. Structural comparisons with canonical cis-splicing machineries uncover adaptations that remodel the trans-spliceosome for SL trans-splicing while preserving core spliceosomal architecture and chemistry. Functional characterization of the DEAH-box helicase PRP22 reveals that its NTD modulates ATPase activity. Together, these findings establish a mechanistic framework for SL trans-splicing and provide insight into the evolutionary diversification of RNA processing in deeply diverged eukaryotes.

## Results

### Capturing SL trans*-*splicing machineries from trypanosomatid parasites

To gain biochemical and structural insight into the catalytically active stages of SL trans*-*splicing in trypanosomatid parasites, we sought to purify endogenous SL trans*-*splicing intermediates from *Leishmania tarentolae* (*Lt*). To this end, we introduced an ectopic copy of affinity-tagged CDC5L, a factor involved in catalytic activation of the trans-spliceosome^20^, into one of the *Lt* rDNA loci (Supplementary Fig. 1a–c). Affinity purification of CDC5L enabled direct isolation of trans-spliceosomes from nuclear extracts. These complexes co-purified with U2, U5, U6, and SL snRNAs, but lack U1 and U4, consistent with their assignment to catalytically activated trans-spliceosomal stages (Supplementary Fig. 1d–f). The predominance of Y-branched endogenous pre-mRNAs, together with only a minor amount of intact SL RNA, indicates that the first transesterification reaction (step I) has largely occurred and that the purified complexes mainly correspond to step II trans-spliceosomes.

To complement this observation at the protein level, we performed mass spectrometry analysis of the purified sample (Supplementary Data 1). This analysis revealed the presence of ubiquitous U2, U5, and U6 snRNP-associated proteins, NTC-NTR components, and step II-specific factors, including SLU7, PRP17, PRKRIP1, CACTIN, the helicase PRP22/DHX8, and components of the exon junction complex (EJC) (Supplementary Fig. 1g). Importantly, proteins characteristic of B^act^, step I or disassembly intermediates were not detected, consistent with enrichment of step II complexes. As expected, many of the identified proteins are highly divergent, showing only 10–30% sequence identity to opisthokont orthologs, and several have no identifiable orthologs (Supplementary Fig. 1h).

Together, the RNA and protein profiles indicate that our sample is highly enriched in step II trans-spliceosomes. This was unexpected as CDC5L is a core component of the NTC normally present from activation through disassembly, suggesting that step II may represent a rate-limiting stage in trypanosomatid SL trans-splicing, with activation and step I occurring more rapidly. Notably, similar affinity strategies targeting core NTC components have also yielded late spliceosomal intermediates, including the isolation of C* complexes upon CDC5L or PRP19 tagging in *Chlamydomonas reinhardtii*^22^ and ILS complexes upon PRP19 tagging in *Caenorhabditis elegans*^23^, consistent with system-specific spliceosomal kinetics. To gain structural insight into these step II trans-spliceosomes, we next analyzed the purified complexes using single-particle cryo-EM.

### Cryo-EM analysis of step II trypanosomatid trans-spliceosomes

Consistent with the biochemical and mass spectrometry data, our cryo-EM analysis revealed two step II assemblies: the trans*-*C* complex, poised for SL exon ligation, and the trans*-*P complex, representing a post-catalytic state in which the SL exon has been ligated to the pre-mRNA. These structures were determined at overall resolutions of 2.7 Å and 2.8 Å, respectively (Supplementary Fig. 2).

To facilitate model building and overall visualization, focused classifications and high-resolution refinements spanning 2.6–3.7 Å resolution were combined into composite maps (Supplementary Figs. 3–5, and Supplementary Table 1). In addition, we performed a systematic pairwise protein-protein interaction (PPI) screen using AlphaFold2-multimer^24^ to support assignment of densities and delineate interaction networks within the complex (Supplementary Fig. 6). Together, the high-quality cryo-EM maps and the predictome enabled the assignment and precise model building of four snRNAs (U2, U5, U6 and SL), one pre-mRNA strand, as well as 68 proteins, including 21 Sm proteins, 7 LSm proteins, and 40 snRNP-associated and splicing factors, corresponding to an overall molecular mass of ~3.2 MDa (Supplementary Table 2).

### Overall structure of step II trypanosomatid trans-spliceosomes

**Fig. 1:**
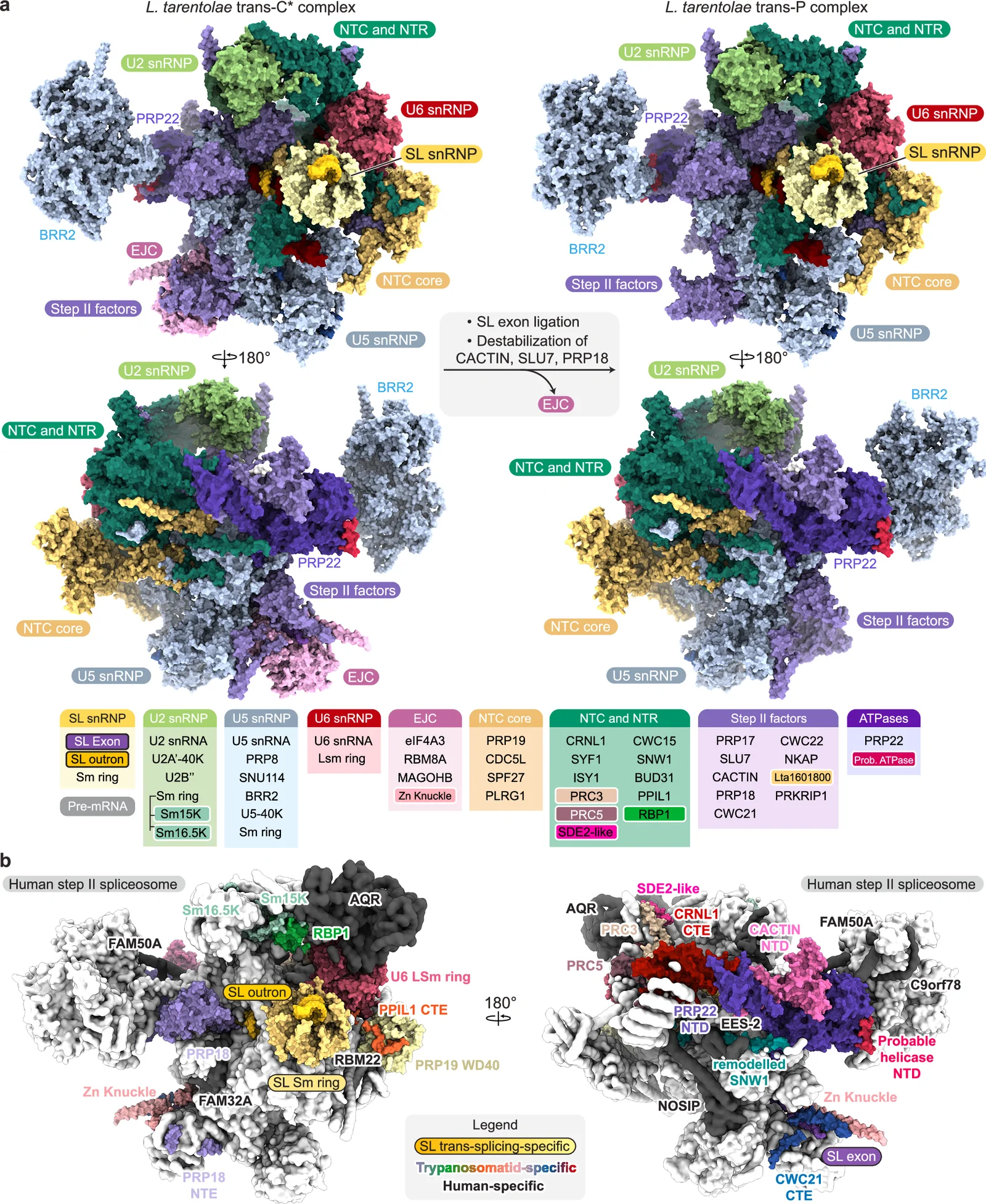
Cryo-EM structure of trypanosomatid step II trans-spliceosomes. **a** Cryo-EM structures of the *L. tarentolae* trans-C* and trans-P complexes. Two views rotated by 180° are shown for each complex. Components are grouped by subcomplex, displayed as colored surfaces, and listed below the structures. Trypanosomatid-specific proteins are highlighted with colored boxes with white borders, and SL trans-splicing-specific RNA species with colored boxes with black borders. **b** Structural comparison between the *L. tarentolae* trans-C* complex and the human step II cis-spliceosome shown in two views rotated by 180°. White surface represents structural elements of the human step II cis-spliceosome shared with the trans-C* complex, whereas components unique to the trans-C* complex are shown in colored surfaces and components unique to the human step II cis-spliceosome are shown in black surfaces. Trypanosomatid-specific proteins and extensions of conserved proteins are indicated with colored labels, whereas SL trans-splicing-specific components are highlighted using boxed labels. Proteins only found in the human step II spliceosome are indicated with black labels. EJC exon junction complex, NTC nineteen complex, NTR NTC-related complex, SL spliced leader.

The overall architecture of the step II trypanosomatid trans-spliceosomes closely resembles that of canonical step II cis-spliceosomes^25–32^ (Fig. 1 and Supplementary Figs. 7, 8). Conserved features include the assembly of U2, U5, and U6 snRNPs into the RNA catalytic core, which is supported by the NTC–NTR network that stabilizes the activated conformation and facilitates the transesterification reactions. Among step II factors, CACTIN, SLU7, and PRP18 are present in the trans-C* complex and become destabilized in the post-catalytic trans-P complex, as observed in the yeast P complex^29^ (Supplementary Fig. 9). The DEAH-box helicase PRP22/DHX8 is poised for mRNA release following SL exon ligation.

Beyond this conserved core, two divergent aspects distinguish the step II trypanosomatid trans-spliceosome: adaptations specific to SL trans-splicing and lineage-specific features that are present across cis- and trans-splicing in trypanosomatids (Fig. 1b). Unique to SL trans-splicing is the SL snRNP, which functions both as an architectural snRNP and as a substrate, providing the SL exon for the step II reaction. Prior to ligation, the SL exon is associated with an extended exon junction complex (EJC). In the post-catalytic state, density corresponding to the EJC is absent, indicating that the EJC is no longer stably associated with the trans-spliceosome through RNA or protein-mediated contacts following SL exon ligation (Fig. 1a and Supplementary Fig. 3). Trypanosomatid-specific adaptations further shape the step II complexes, including retention of the U6 LSm ring, incorporation of numerous lineage-specific proteins such as Sm16.5 K, Sm15K, PRC3, PRC5, SDE2-like and RBP1, and structural expansions and remodeling of conserved modules, including PRP18 NTE, CRNL1 CTE, CACTIN NTD, PRP22 NTD, CWC21 CTE, and PPIL1 CTE domains (Fig. 1b). These distinctive SL trans-splicing and trypanosomatid-specific features are described in more detail in the following sections. For an in-depth view of these states at the atomic coordinate level, PyMOL sessions and movies are available (Supplementary Data 2, 3, and Supplementary Movies 1, 2).

### Mechanisms of trypanosomatid step II SL trans-splicing

**Fig. 2:**
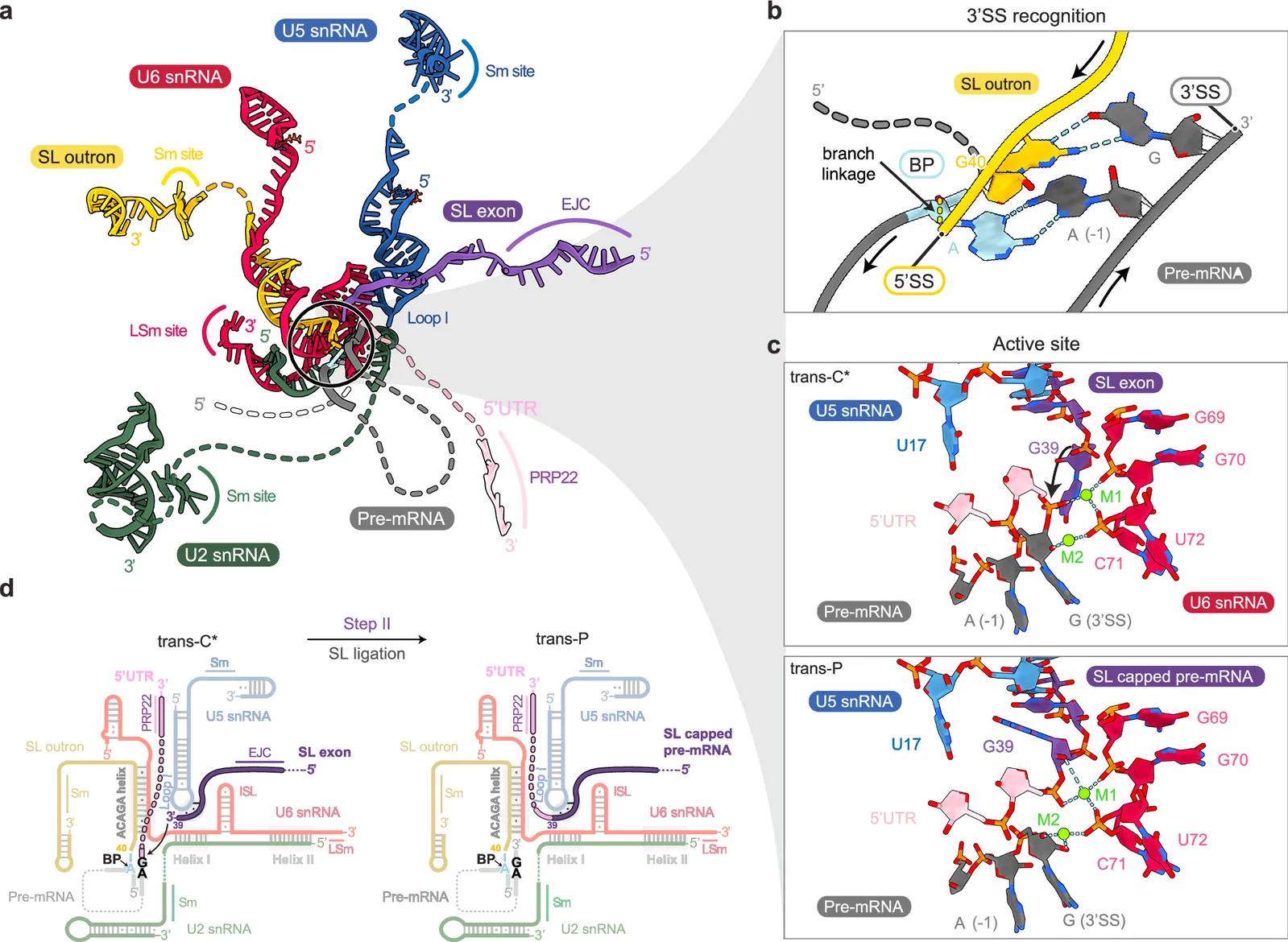
Mechanisms of trypanosomatid step II during SL trans-splicing. **a** Overview of the snRNA elements and pre-mRNA within the step II trans-spliceosome, highlighting the SL snRNA (exon and outron), as well as U2, U5, and U6 snRNAs, and the pre-mRNA. Protein binding sites, including Sm, LSm, EJC, and PRP22 are indicated. The disordered RNA sequences are indicated by dashed lines. **b** The 3’SS is recognized through non–Watson–Crick pairing, with the 3’SS G pairing with 5’SS G40 of the SL outron and the 3’SS A − 1 pairing with the branchpoint adenosine. **c** Active site architecture of the trans-C* (top) and trans-P (bottom) complexes showing key nucleotides of the SL exon, U5 snRNA, U6 snRNA, and pre-mRNA, together with coordinating catalytic Mg^2+^ ions (M1, M2). **d** Schematic comparison of the trans-C* and trans-P complexes, illustrating the step II transition of SL trans-splicing before and after SL exon ligation. 3’SS 3’ splice site, 5’SS 5’ splice site, EJC exon junction complex, SL spliced leader.

The step II trypanosomatid trans-spliceosomes reveal a highly organized and minimal RNA network orchestrating SL exon ligation (Fig. 2 and Supplementary Fig. 10). The U2 snRNP stabilizes the catalytic RNA core by maintaining interactions with U6 snRNA while positioning protein factors essential for SL exon ligation, including CACTIN, PRP17, SLU7, PRKRIP1 and PRP18 (Supplementary Figs. 8g, 10). U5 snRNA, unusually short in trypanosomatids^33,34^, aligns the 5’ splice site (5’SS) of the SL exon with the 3’ splice site (3’SS) of the pre-mRNA via loop I (Fig. 2c, d and Supplementary Figs. 10, 11), while PRP8 coordinates critical interactions at the catalytic core through its conserved helices in RT and NTD domains (Supplementary Fig. 12). Interestingly, despite previous reports suggesting the absence of a cap modification on U5 snRNA in trypanosomatids^33,34^, we identified an m⁷G modification on its first nucleotide, which base-pairs with C35 and C36 of U5 snRNA, further stabilizing U5 stem I (Supplementary Fig. 10f). U6 snRNA forms base-pairing interactions with U2 snRNA and the 5’ SS, coordinates the catalytic Mg^2^⁺ ions, and positions the splice sites for efficient SL exon ligation (Fig. 2c and Supplementary Fig. 10e). Through step I of SL trans-splicing, the SL snRNA is already cleaved at the 5’SS, producing the SL exon and branched SL outron. The SL exon is bound at its 5’ end by the exon junction complex (EJC) in the trans-C* complex and is further stabilized by U5 loop I and PRP8. The branched SL outron extensively pairs with the conserved U6 ACAGA helix and assembles into the SL snRNP with a canonical Sm ring (Fig. 2a, d and Supplementary Fig. 10).

The catalytic site remains highly conserved compared to the cis-spliceosome in opisthokonts, with base-pairing interactions stabilizing the 3’SS on the pre-mRNA and the 5’SS on the SL snRNA. Specifically, the Hoogsteen edge of the 3’SS G pairs with the Watson–Crick edge of the 5’SS G40 of the SL outron, while 3’SS A-1 forms a non-Watson-Crick pair with the branchpoint adenosine, mirroring interactions observed in yeast and humans^25–32^ (Fig. 2b, and Supplementary Fig. 13). In the trans-C* complex, before the SL exon is ligated to the pre-mRNA, the terminal nucleotide of the SL exon (G39) adopts a flipped-out conformation, positioned away from the 5’ UTR and lacking stabilizing interactions with U17 of the U5 snRNA or coordination with the M1 catalytic metal ion. In contrast to the human C* complex where the equivalent exon nucleotide is stabilized by FAM32 and U5 snRNA in a reaction-ready orientation, trypanosomatids lack a FAM32 ortholog. Based on this comparative structural analysis, we propose that, in the absence of these stabilizing interactions, G39 samples alternative conformations in the trans-C* complex until productive alignment for the second trans-esterification reaction is achieved. In the trans-P complex G39 adopts an inserted conformation and is ligated to the 5′ UTR of the pre-mRNA (Fig. 2c and Supplementary Fig. 12).

Interestingly, nucleotides immediately surrounding the branchpoint adenosine remain flexible and are not stabilized by base pairing with U2 snRNA (Supplementary Figs. 10, 13). The lack of EM density at the branchpoint likely reflects the absence of a conserved branchpoint recognition motif in both the U2 snRNA and the pre-mRNA involved in trypanosomatid SL trans-splicing^11,35^, which may hinder formation of stable interactions. Consistently, the branch helix upstream of the branchpoint, normally formed by base pairing between the pre-mRNA and U2 snRNA, is not resolved in our structures. This apparent flexibility and lack of defined density could be further influenced by the heterogeneity of endogenous pre-mRNA substrates in our sample. By contrast, in human spliceosomes, even highly variable pre-mRNA branch sequences can still engage with the conserved U2 branchpoint recognition motif, producing a defined branch helix despite sequence degeneracy^23,28^. Together, these observations are consistent with the view that branchpoint selection in trypanosomatids is primarily guided by the downstream polypyrimidine tract during early stages of trans-spliceosome assembly, as also observed in metazoans. Still, the underlying molecular mechanism remains incompletely understood in trypanosomatids, as the precise roles of U2AF35, U2AF65, and SF1 in branchpoint recognition and U2 snRNP recruitment are still not fully defined^36,37^.

### Adaptations associated with SL trans-splicing

While the catalytic chemistry is conserved between SL trans-splicing and intron-removal cis-splicing, several protein adaptations associated with the intronless nature of SL trans-splicing are evident at the structural level. Proteins that typically stabilize introns in cis-spliceosomes, such as RBM22 (Cwc2/Ecm2 in yeast) and the AQR helicase^27,30,32^, are absent. In this context, the Sm ring of the SL snRNP occupies the position normally taken by RBM22, protruding outward atop BUD31, and provides an alternative scaffold that stabilizes the U6–SL outron helix (Supplementary Fig. 14a). The absence of these intron-stabilizing factors is also accompanied by extensive rewiring of the surrounding protein network. For example, PPIL1 in trypanosomatids has evolved an extended C-terminal tail that engages the WD40 domain of PRP19 atop the PRP19 tetramer, compensating for the simplified interface caused by the loss of RBM22. The N-terminal tails of PRP17 and SNW1 adopt new contacts with PPIL1, creating a distinct interface that differs markedly from human and yeast spliceosomes. At the NTC core, interactions between SNW1, CDC5L, and PRP17 are reorganized: in humans, RBM22 stabilizes an extensive network between these proteins, whereas in trypanosomatids, alternative contacts form directly among SNW1, CDC5L, and PRP17 to maintain stability of the catalytic site and the U6–SL outron helix (Supplementary Fig. 14b). Together, these examples illustrate how trypanosomatids have reconfigured protein–protein interactions to stabilize RNA duplexes forming the catalytic core in the absence of canonical intron-associated factors, highlighting a structural architecture adapted for SL trans-splicing while preserving the fundamental chemistry of exon ligation.

### Recognition of the SL exon by the Exon Junction Complex

**Fig. 3:**
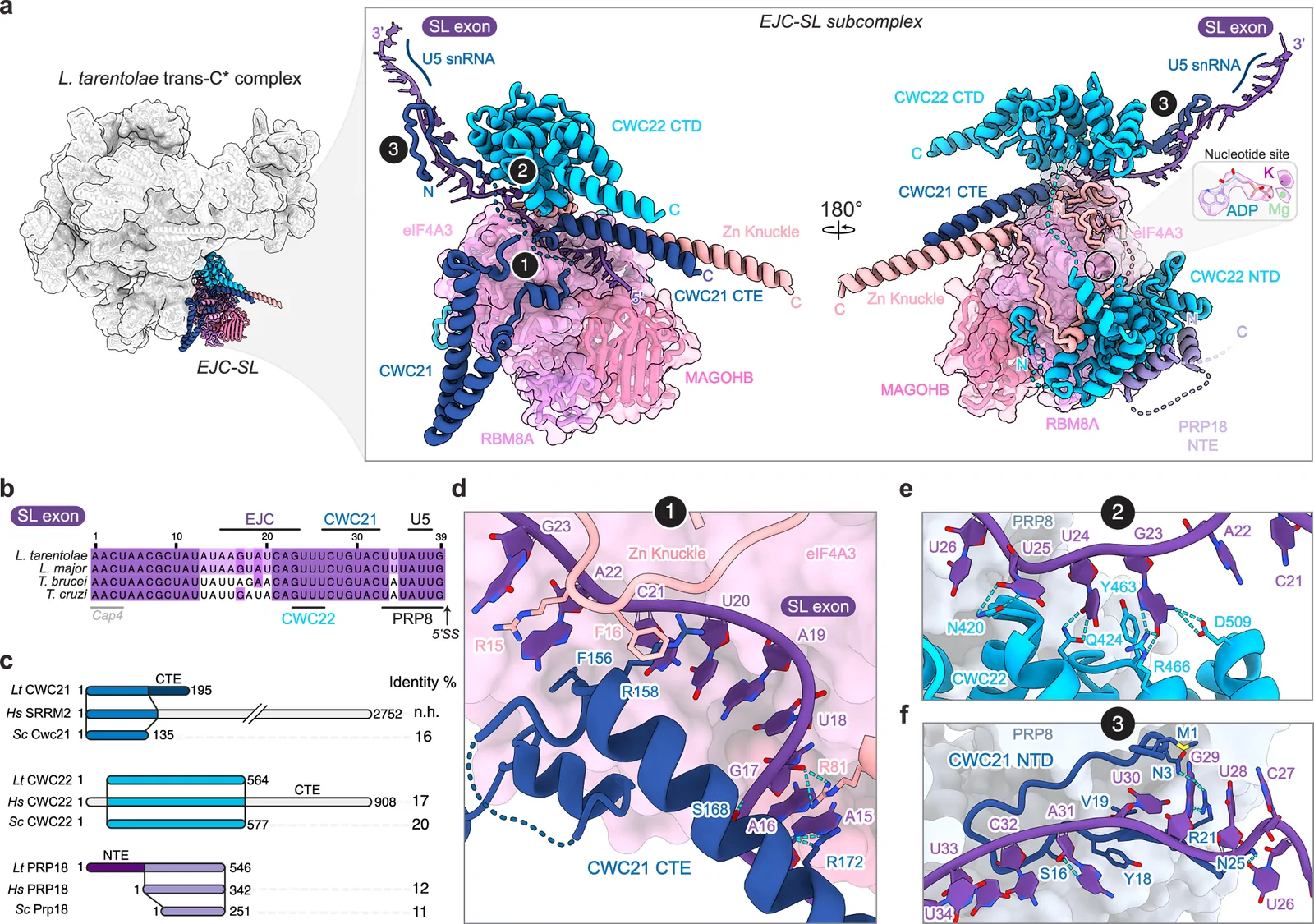
Recognition of the SL exon by an expanded Exon Junction Complex. **a** Structure of the *L. tarentolae* trans-C* complex highlighting the exon junction complex–spliced leader RNA (EJC–SL) subcomplex (colored) within the full trans-spliceosome (gray). Inset, two views of the EJC–SL subcomplex showing SL exon recognition by U5 snRNA and the protein components CWC22 (NTD and CTD), CWC21 (CTE), Zn Knuckle, MAGOHB, RBM8A, eIF4A3, and PRP18 (NTE). The nucleotide-binding site of eIF4A3 is shown with ADP and Mg²⁺. PRP8 is not shown for clarity. **b** Sequence alignment of the SL exon across trypanosomatids. **c** Domain organization of *L. tarentolae* CWC21, CWC22 and PRP18 compared with human (*Hs*) and yeast (*Sc*) orthologs, with percentage identities indicated. **d** Close-up view of the CWC21 CTE and Zinc knuckle stabilizing nucleotides A15–G23 of the SL exon through non-specific phosphate backbone and ribose contacts, as well as pi-stacking interactions. **e** Close-up view of CWC22 CTD interactions with nucleotides G23–U25 of the SL exon. **f** Close-up view of CWC21 NTD and PRP8 interacting with nucleotides C27–U33 of the SL exon. CTD C-terminal domain, CTE C-terminal extension, NTD N-terminal domain, NTE N-terminal extension.

Although previous studies were inconclusive regarding the presence of a functional EJC in trypanosomatids and its involvement in trans-splicing^38,39^, our structural data reveal a complete and well-ordered EJC assembled on the SL exon in the trans-C* complex (Fig. 3). This complex contains the three conserved core components, eIF4A3, MAGOH, and RBM8A, together with an additional Zn-knuckle factor, which appears to bind at the same position as the metazoan EJC factor CASC3, a protein that promotes transcriptome-wide activation of nonsense-mediated decay (NMD)^40^ (Fig. 3a and Supplementary Fig. 15). Beyond this canonical core, trypanosomatids display an expanded interaction network where the CWC21 CTE and the Zn-knuckle C-terminal helix further stabilize the SL exon within the EJC, acting as additional anchoring elements (Fig. 3a, c, d). We also identified the extended N-terminus of PRP18 forming direct contacts with the CWC22 NTD, thereby coupling SL exon binding by the EJC with SL exon ligation sensing through the PRP18 insertion loop into the catalytic site (Fig. 3a and Supplementary Figs. 8g and 15d, e). Destabilization of PRP18 following SL exon ligation (Supplementary Fig. 9) may therefore disrupt this interface and contribute to the release of the EJC before PRP22 acts to release the spliced SL-capped pre-mRNA.

A key distinction from metazoans is the conservation of the SL exon sequence, in contrast to the highly variable 5’ exons typically engaged by the metazoan EJC (Fig. 3b and Supplementary Fig. 11c). This conservation, extending across the full SL RNA (Supplementary Fig. 11c), is presumably required for its biogenesis and proper folding into the mature SL snRNP before recruitment to the trans-spliceosome^41–43^. Many interactions between the SL exon and CWC21 and CWC22, involve specific amino-acids and RNA bases (Fig. 3e, f), whereas the EJC binds a slightly variable region on the SL exon between residues 15-23, stabilized by phosphate backbone and ribose contacts, and pi-stacking interactions with RNA bases that allow sequence flexibility without loss of recognition (Fig. 3b, d).

Strikingly, as opposed to cryo-EM structures of cis-C/C*/P complexes where ATP-bound eIF4A3 is modeled, we unambiguously identified ADP bound to eIF4A3 (Fig. 3a). Structural and biochemical studies have shown that eIF4A3 clamps RNA in a closed conformation upon ATP binding, a state further stabilized by the MAGOH–RBM8A heterodimer, which prevents nucleotide exchange and reopening of the helicase core after ATP hydrolysis^44–46^. Consistent with this mechanism of post-hydrolysis inhibition, our data suggest that ATP hydrolysis occurs within the assembled EJC, generating an ADP-bound eIF4A3 that remains in a closed conformation, clamping the EJC onto the SL exon in the trans-C* complex. Because ATP hydrolysis reduces RNA affinity in DEAD-box helicases^47,48^, this post-hydrolysis state may also prime the complex for dissociation from the SL RNA once EJC and spliceosomal protein-protein interactions are destabilized upon step II factors release. In agreement with this model, density corresponding to the EJC is absent in the post-catalytic trans-P complex, indicating that the EJC is no longer associated with the trans-spliceosome following SL exon ligation. Whether this ADP-bound state reflects a general feature of EJC dynamics during splicing or instead represents a species-specific adaptation associated with trans-splicing in trypanosomatids or a trans-C* substate after ATP hydrolysis remains unclear, particularly given the limited high-resolution structural information available for EJCs in spliceosomes from other eukaryotes^23,25,31,49^.

### Structural innovations of trypanosomatid spliceosomal proteins

**Fig. 4:**
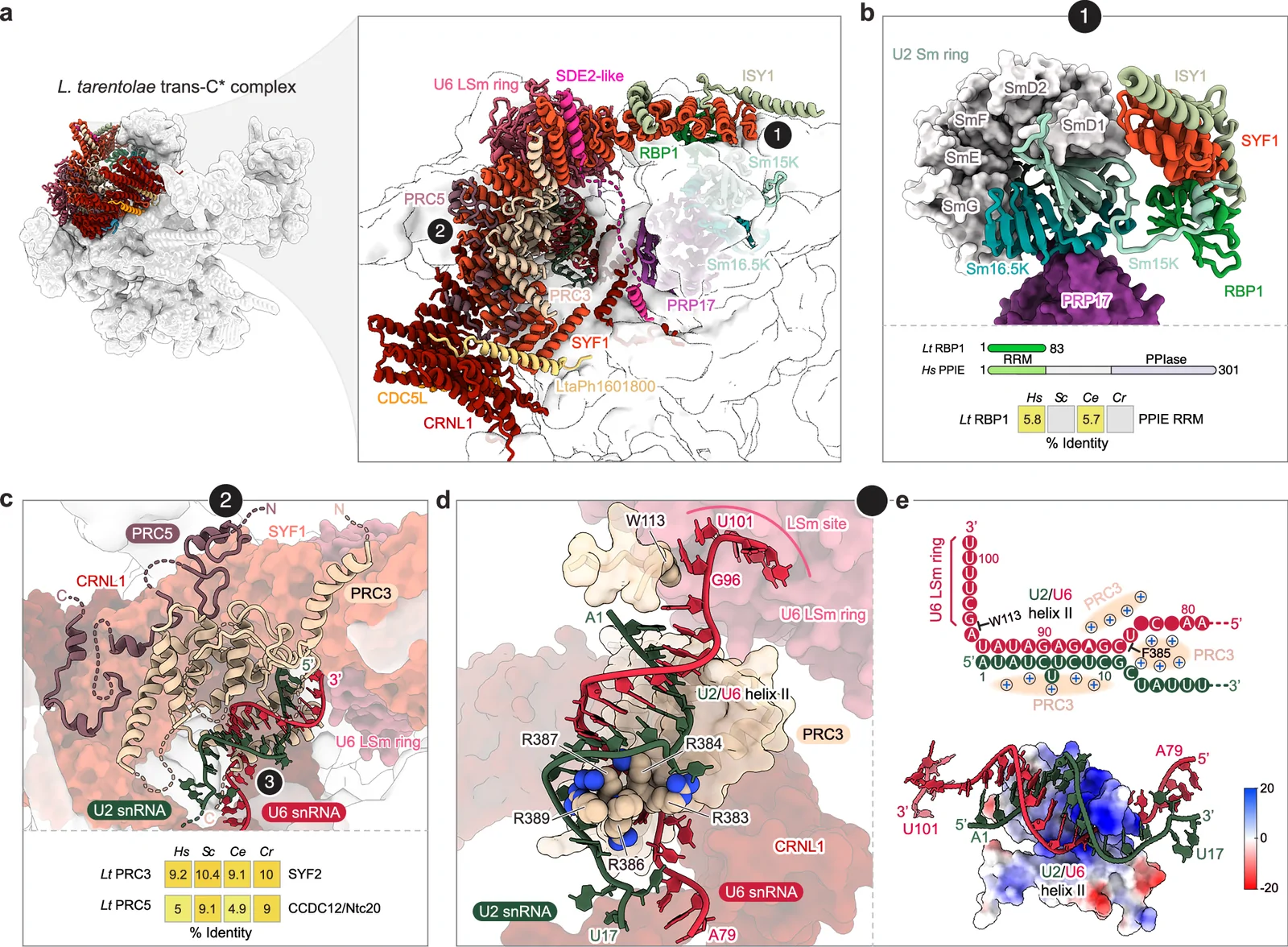
Trypanosomatid-specific adaptations for SL trans-splicing. **a** Structure of the *L. tarentolae* trans-C* complex showing the region containing U2/U6 RNAs and associated proteins (colored) within the overall complex (gray). The inset highlights RBP1, ISY1, Sm15 K/Sm16.5 K, PRP17, PRC3, PRC5, SYF1, SDE2-like, CDC5L, CRNL1, LtaPh1601860, and the U6 LSm ring. **b** Close-up view of the U2 Sm ring, containing the trypanosomatid-specific variants Sm15K and Sm16.5K interacting with RBP1 and SYF1. Domain organization of RBP1 compared with human PPIE is shown, including sequence conservation. **c** Close-up view of interactions of PRC3 and PRC5 with CRNL1, SYF1, and U2/U6 helix II. Sequence conservation of PRC3 and PRC5 with functional analogs in human (*Hs*), yeast (*Sc*), and *C. reinhardtii* (*Cr*) is indicated below. **d** Close-up view of PRC3 interactions stabilizing U2/U6 helix II mediated by key residues R383, R384, F385, R386, R389 and W113. **e** Model of U2/U6 helix II interactions. Top, schematic of RNA secondary structure showing PRC3 and U2/U6 contacts. Bottom, electrostatic surface potential of PRC3 highlighting RNA-binding grooves.

A defining feature of the step II trans-spliceosome is the post-activation retention of the U6 LSm ring and its stable docking onto the NTR scaffold formed by SYF1 and CRNL1, in a position normally occupied by the intron-binding helicase AQR in metazoans step II spliceosomes^27,30^ (Figs. 1a and 4, Supplementary Fig. 14a). This retention is structurally enabled by two features: the absence of AQR in trypanosomatids, leaving its binding site on SYF1 vacant, and the shortened 3′ end of U6 snRNA, which maintains the LSm ring in close proximity to the NTR scaffold. The LSm ring establishes tight contacts with SYF1 and PPIL1 through extensions of its LSm subunits, effectively bridging the NTC core and the NTR and reinforcing the integrity of the NTC–NTR assembly. As a consequence of this interaction network, U2/U6 helix II adopts and maintains a distinctive bent conformation (Supplementary Fig. 16b). Together, these interactions position the LSm ring as an integral structural component of the activated trans-spliceosome rather than a transient pre-activation factor, representing a major divergence from canonical splicing systems. Indeed, in yeast and human spliceosomes, the U6 LSm ring is released during activation between the pre-B^act-I^ and pre-B^act-II^ stages^50^, and its dissociation is considered a hallmark of catalytic activation^51,52^. The persistence of the LSm ring raises mechanistic questions. In opisthokonts, release of the LSm ring exposes the 3′ end of U6 for engagement by DHX15 during spliceosome disassembly^23^. In trypanosomatids, by contrast, the 3′ end remains shielded by the LSm ring in step II complexes. Whether the LSm ring is displaced at a later stage or whether disassembly proceeds via a distinct mechanism remains unknown. Because the molecular choreography of activation and disassembly has not yet been structurally characterized in trypanosomatids, it is unclear whether LSm retention represents a compensatory adaptation to the absence of AQR or reflects a broader divergence in spliceosome dynamics.

Beyond U6 LSm ring retention, the trypanosomatid NTR provides a platform for several lineage-specific proteins, including PRC3, PRC5, SDE2-like, LtaPh1601800, and RBP1 (Fig. 4a). PRC3 and PRC5, essential for cell viability and pre-mRNA cis- and trans-splicing in trypanosomes^14^, establish an extensive network of interactions with SYF1 and CRNL1 that further stabilizes the distinctive U2/U6 helix II conformation induced by the retained LSm ring (Fig. 4c). PRC3 directly engages the RNA duplex through a highly positively charged groove formed by five nearly consecutive arginine residues within its C-terminal helix, while W113 stabilizes the duplex tip by stacking against U6 G96 and U2 A1 (Fig. 4d, e). Although PRC3 and PRC5 occupy positions analogous to SYF2/Syf2 and CCD12/Ntc20 in human and yeast spliceosomes, their interaction patterns differ substantially, reflecting lineage-specific adaptations (Supplementary Fig. 16a, b). Notably, the essential roles of PRC3 and PRC5 in both cis- and trans-splicing indicate that stabilization of the retained U6 LSm ring is a lineage-wide feature of trypanosomatid spliceosomes rather than a trans-splicing–specific adaptation. This architectural reorganization propagates to surrounding canonical proteins, including CDC5L, SNW1, and CWC15, which adopt altered interaction modes to accommodate the modified U2/U6 helix II configuration (Supplementary Fig. 16b–f).

The U2 snRNP contains two trypanosomatid-specific Sm proteins, Sm15 K and Sm16.5K^12,13,19^. Sm15K features an extended C-terminal tail that interacts with the small RRM protein RBP1, functionally replacing the RRM domain of human PPIE (Fig. 4b). In addition, we identified an unexpected SDE2-like protein that undergoes proteolytic processing via cleavage of its N-terminal ubiquitin-like domain, a mechanism previously described in opisthokonts^53,54^ (Supplementary Fig. 17a, b). This processing event generates the mature SDE2-C fragment, which we observe incorporated into the trans-spliceosome catalytic core near the U2/U6 helix I. In this position, SDE2-C contributes to stabilization of the active site, while its C-terminal helix docks onto SYF1 above the U2/U6 helix II in a manner analogous to the human C* complex^28^ (Supplementary Fig. 17c). Although no direct functional inference can be drawn from our data alone, its conserved positioning suggests a potential role in modulating 3’SS selection, similar to SDE2 in metazoans^53^ (Fig. 4a and Supplementary Fig. 17c). Similarly, LtaPh1601800 associates with CRNL1 at a position corresponding to that occupied by FAM192A in humans and Fyv6 in yeast C* complexes (Supplementary Fig. 18), both implicated in 3′ splice-site selection^29,31^. While this structural convergence does not establish a direct functional role, the conserved spatial organization suggests that LtaPh1601800 may fulfill an analogous function in trypanosomatids. Further biochemical and genetic analyses will be required to test this hypothesis. Together, these observations reveal that trypanosomatid spliceosomes combine lineage-specific innovations with unexpected structural analogs of opisthokont factors, pointing to convergent evolution of protein architectures that support catalytic competence.

### Trypanosomatid-specific network of the DEAH-box helicase PRP22/DHX8

**Fig. 5:**
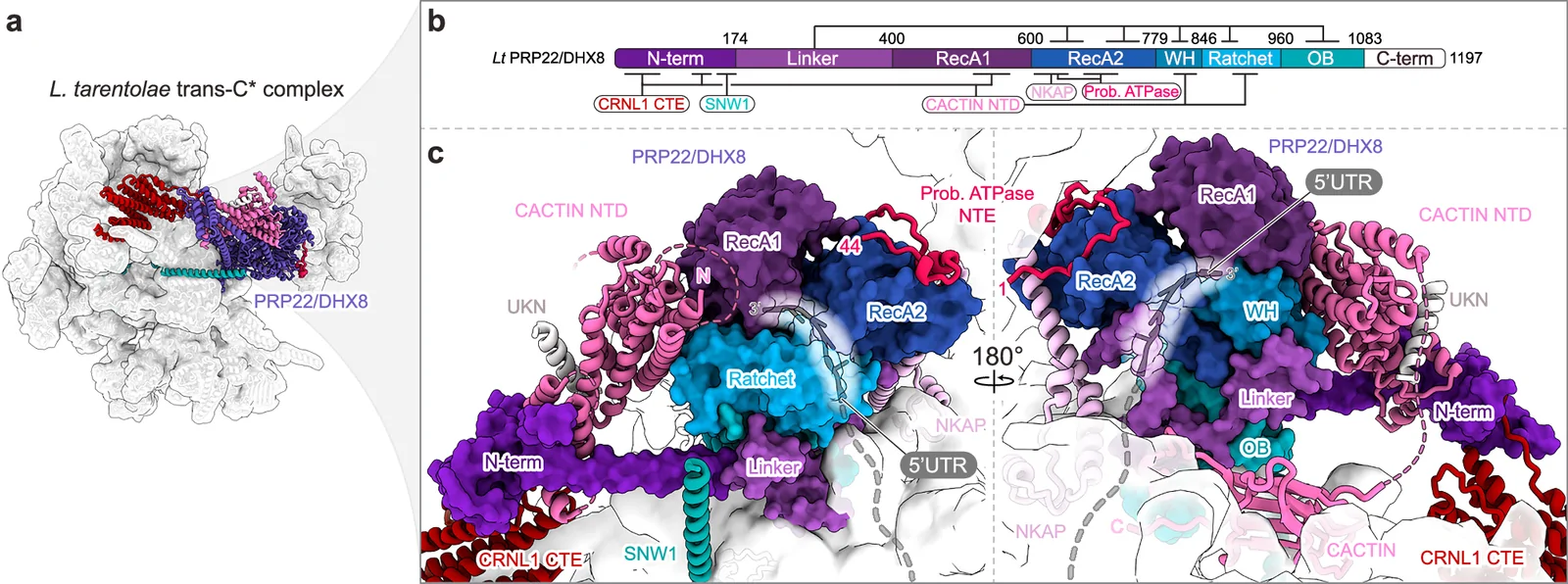
Trypanosomatid protein network locks PRP22 to prevent premature mRNP release. **a** Structure of the *L. tarentolae* trans-C* complex, highlighting PRP22/DHX8 and its interactors (colored) within the overall complex (gray). **b** Domain architecture of PRP22, showing conserved DEAH-box helicase modules (RecA1, RecA2, WH, Ratchet, OB), flanking regions (N-term, linker, C-term), and binding partners (CRNL1 CTE, SNW1, CACTIN NTD, NKAP, and a probable helicase NTE). **c** Two orientations of RNA-bound PRP22 and its regulators in the trans-C* complex. PRP22/DHX8 is locked in a pre-activated conformation on the trans-spliceosome through a network of domains, including CRNL1 CTE, PRP22 NTD, SNW1, CACTIN NTD, and NKAP. NTE N-terminal extension, OB Oligonucleotide/Oligosaccharide-Binding domain, UKN unknown protein, WH winged-helix domain.

In both trans-C*/P complexes, we captured PRP22/DHX8 in two conformations distinguished by the position of its RecA2 lobe, either open or closed onto the pre-mRNA, showing that PRP22 can exist in an equilibrium of conformations sensing the pre-mRNA while being docked on the trans-spliceosome. Yet, in both conformations, the helicase adopts a tightly bent conformation without bound nucleotide, locked by an extended network of trypanosomatid-specific interactions (Fig. 5 and Supplementary Figs. 4, 19a, c). The modified CRNL1 CTE engages PRP22 N-terminal helical bundle, which, together with the CACTIN NTD, is sandwiched against the Ratchet, RecA1, and WH domains. On the opposite side, NKAP braces the labile RecA2 lobe, while the C-terminal helix of SNW1 further stabilizes the PRP22 NTD. We also observed an additional unidentified factor (UNK) bound to CACTIN NTD, suggesting additional layers of protein-protein interactions. Moreover, we were able to trace the PRP22 linker, which makes extensive contacts with its WH, OB, and Ratchet domains. Strikingly, these interactions overlap with the binding sites of G-patch proteins that regulate DHX15 activity and RNA affinity in opisthokonts^55^ (Supplementary Fig. 19d). Consistently, the NTD linker in the yeast and human homologs binds to the same region as visualized in recent cryo-EM structures^23,29^, highlighting a conserved mode of interaction between the linker and the helicase core in PRP22/DHX8 (Supplementary Fig. 19e). Based on these observations, we hypothesized that the linker region may modulate PRP22 activity, analogous to how G-patch proteins regulate other DExD/H-box helicases. To test this, we attempted to overexpress in *E. coli* two constructs of the helicase, a full-length protein (PRP22^FL^) and a truncated variant lacking the NTD linker (PRP22^ΔNTD^). However, both variants proved difficult to purify in a soluble form. To overcome this limitation, we generated *L. tarentol*ae cell lines constitutively expressing tagged versions of each construct. This enabled their purification in soluble and pure form, although yields remained low (~10 µg per 6 L of culture) (Supplementary Fig. 20a–i). We then compared their ATPase activity using a continuous coupled assay. PRP22^ΔNTD^ displayed a ~ 3-fold increased ATP turnover rate (*K*_cat_ = 43.29 ± 0.79 min⁻¹) compared to the full-length enzyme (*K*_cat_ = 16.4 ± 0.25 min⁻¹), showing that the NTD-linker exert an inhibitory effect on ATPase activity (Supplementary Fig. 20j, k). This observation is consistent with reported ATPase activity of the human homolog DHX8, in which the ΔNTD variant exhibits a ~ 4-fold increase in ATP turnover rate^56^, further supporting the idea that the NTD-linker may function as a conserved autoregulatory module. Further mutagenesis targeting residues involved in linker-helicase interactions will be required to dissect this regulatory mechanism at the residue level.

Finally, we identified density corresponding to the N-terminal extension of an uncharacterized protein (LtaP16.0490) decorating the RecA2 lobe of PRP22 and stabilizing NKAP (Fig. 5e and Supplementary Fig. 21a–c). Structural similarity searches with FoldSeek revealed that this protein adopts the fold of an SF2 DExD/H helicase/ATPase, characterized by two RecA domains. Interestingly, multiple sequence alignments show that it contains numerous insertion loops and harbors degenerate Walker A (GIGKR instead of the canonical GxGKT) and Walker B (ANLE instead of the canonical DExD/H) motifs (Supplementary Fig. 21d–f). Given these deviations from canonical SF2 motifs, we sought to experimentally assess whether this protein retains ATPase activity. The purified protein revealed an intrinsic ATP turnover rate (*K*_cat_ = 0.15 ± 0.04 min^−1^) comparable to the human DHX15 (*K*_cat_ = 0.168 min^−1^)^55^, supporting the classification of LtaP16.0490 as a bona fide ATPase despite degenerate ATP-binding motifs (Supplementary Fig. 21g, h). Together, these data establish that a second non-canonical ATPase associates with PRP22/DHX8, although its precise functional role in SL trans-splicing remains to be determined (Supplementary Fig. 21i). Interestingly, in humans, PRP22/DHX8 has also been reported to associate with an additional SF2 RNA helicase, DDX41^28^, suggesting that docking of a second ATPase onto PRP22 may represent a conserved architectural feature across eukaryotes.

## Discussion

**Fig. 6:**
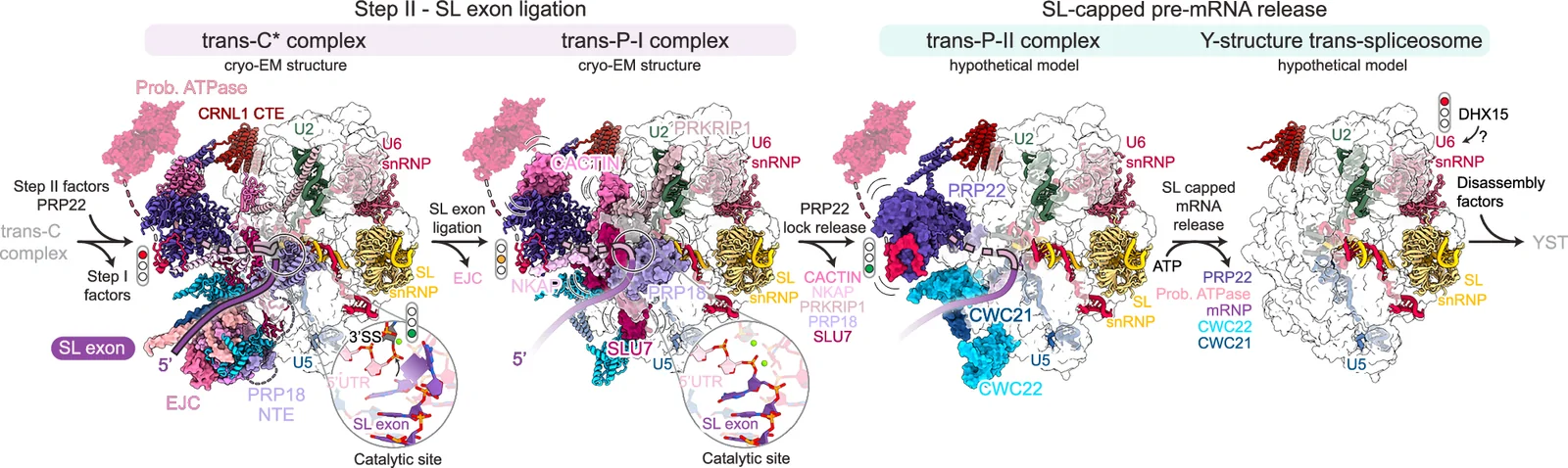
Mechanistic model of step II SL trans-splicing and SL-capped mRNA release in trypanosomatid parasites. Step II factors, including CACTIN, SLU7, NKAP, PRKRIP1, and PRP18, position and stabilize the SL exon and 3’SS in the trans-C* complex to catalyze exon ligation. In the absence of FAM32A, the last base of the SL exon alternates between retracted and engaged conformations, suggesting a trypanosomatid-specific mechanism for controlling the timing of SL exon ligation. In the trans-P complex, a first set of step II factors becomes destabilized and begins to dissociate after ligation, including PRP18, which transmits the signal of exon ligation to the EJC via its NTE, leading to EJC detachment. Following the complete dissociation of step II factors, the lock enforced by CRNL1 CTE, CACTIN NTD, PRP22 NTD and NKAP restraining PRP22 is released. Fully activated PRP22 then releases the ligated SL pre-mRNA together with a second set of step II factors, leaving the trans-spliceosome poised for disassembly. Factors represented in surfaces dissociates in the next state. 3’SS 3’ splice site, CTD C-terminal domain, CTE C-terminal extension, EJC exon junction complex, NTD N-terminal domain, NTE N-terminal extension, SL spliced leader.

We present structural snapshots of SL trans-splicing machineries from trypanosomatid parasites, providing a near-atomic inventory of their divergent RNA and protein components and revealing the molecular basis of the step II reaction, thereby establishing a structural framework for decades of biochemical studies^6,7,14,17,18,20^. An emerging model for the late stages of SL trans-splicing highlights how trypanosomatid-specific protein adaptations coordinate the conserved chemistry of the second catalytic step and orchestrate orderly mRNA release (Fig. 6). First, in the trans-C* complex, the SL exon and 3’SS are precisely positioned and stabilized for the second catalytic step by step II factors, including CACTIN, SLU7, NKAP, PRKRIP1, and PRP18. Second, in the trans-P complex, a first set of step II factors is destabilized and begins to dissociate following SL exon ligation. Through its NTE, PRP18 transmits these events to the EJC, which dissociates from the SL exon and trans-spliceosome following SL exon ligation. Third, during step II, PRP22 remains stably docked onto the trans-spliceosome in a pre-activated, nucleotide-free state, sampling the pre-mRNA substrate. Fourth, after complete dissociation of step II catalytic factors, the lock formed by CRNL1 CTE, CACTIN NTD, PRP22 NTD, and NKAP, which had restrained PRP22 from translocating RNA, is released. Finally, the ligated SL pre-mRNA is released by fully activated PRP22, which dissociates along with a second set of step II factors, leaving the trans-spliceosome poised for disassembly. In the subsequent steps, DHX15 presumably unwinds the 3’ end of U6 snRNA to initiate Y-structure disassembly and trans-spliceosome recycling, but how it gains access to its substrate while the LSm ring remains bound is still unclear.

Despite their deep evolutionary divergence from opisthokonts, trypanosomatids retain a highly conserved spliceosomal catalytic core, which preserves essential features such as 3’SS recognition, metal-ion coordination, and precise pre-mRNA alignment with the SL RNA. In this arrangement, the ACAGA helix of U6 snRNA stabilizes the SL outron, while U5 snRNA positions the invariant SL exon within the catalytic core for ligation, a process further reinforced by step II factors that secure the 3’SS. While the catalytic chemistry of exon ligation is conserved, the overall architecture of the trans-spliceosome has been extensively remodeled by lineage-specific innovations. Multiple canonical proteins, including intron-stabilizing factors, are replaced by trypanosomatid-specific components, while extensions on proteins create new binding platforms that stabilize RNA, compensate for altered RNA conformations, and substitute for missing canonical RNA motifs. Altogether, these adaptations establish multilayered regulatory networks that restrain helicase activity, stabilize transient intermediates, participate in signal transduction, and ensure precise exon ligation before mRNA release.

Our structures further reveal that a complete exon junction complex (EJC) assembles on the conserved SL exon during trans-splicing. Unlike metazoans, where EJCs are deposited on multiple spliced exons and serve as critical markers for NMD, trypanosomatids are largely intronless, suggesting that the EJC is unlikely to participate in NMD^39^. Instead, the conserved SL exon provides a single EJC-binding platform that assembles transiently during trans-splicing. The release of the EJC following SL exon ligation suggests that, unlike in metazoans where EJCs remain associated with mRNAs until the pioneer round of translation and act as persistent markers for mRNP transport and downstream regulation^57^, EJC binding in trypanosomatids is likely transient. Rather than serving as a stable mRNA landmark, the EJC may instead stabilize the SL exon prior to ligation and signal completion of the splicing reaction upon its dissociation. This behavior therefore appears distinct from the canonical metazoan paradigm and may reflect species-specific adaptations associated with the unique organization of trypanosomatid gene expression. In this context, transient EJC assembly could provide a checkpoint that ensures proper assembly and catalysis of the trans-spliceosome before release of the mature SL-capped mRNA. Because the mechanisms governing mRNA export in trypanosomatids remain incompletely understood, it is currently unclear whether the EJC contributes to downstream mRNP maturation in these organisms^58,59^.

Altogether, this study establishes a structural blueprint for SL trans-splicing, laying the groundwork for mechanistic studies of other stages in this essential pathway in trypanosomatid parasites. The structures show how highly divergent organisms have evolved distinct protein solutions to perform conserved spliceosomal function, underscoring both the universality of the principles underlying spliceosomal step II catalysis and the evolutionary flexibility inherited from the ancestral eukaryotic spliceosome. Because SL trans-splicing is indispensable for the generation of every mature mRNA in trypanosomatids, its machinery constitutes a major vulnerability. Many of the divergent proteins and regulatory networks uncovered, lacking clear opisthokont counterparts, represent promising targets for anti-parasitic drugs, offering new therapeutic opportunities against sleeping sickness, Chagas disease, and leishmaniasis.

## Methods

### Identification of CDC5L homolog in *L. tarentolae*

The annotated CDC5L protein sequence and AlphaFold prediction from *T. brucei* (Uniprot: Q57ZS5_TRYB2; TriTryp: Tb927.5.2060) were both used as inputs for blastp and Foldseek^60^ searches, respectively, to identify the gene coding for *L. tarentolae* CDC5L (Uniprot: A0A640KDH5; TriTryp: LtaPh_1708300).

### Generation of *L. tarentolae* cell line for ectopic expression of CDC5L-GFP

To constitutively express CDC5L-GFP in *L. tarentolae* from a genome-integrated position, the gene coding for *L. tarentolae* CDC5L was codon optimized for expression in *Leishmania* and cloned into a pLEXSY-hyg2.1 vector (Jena Bioscience) with a C-terminal dual affinity tag (TEV-cleavable HA, 3C-cleavable GFP) (Supplementary Fig. 1a). For transfection, the density of *L. tarentolae* P10 cells (Jena Bioscience) was adjusted to 10⁸ cells/mL in the P3 Primary Cell buffer (Lonza) and incubated on ice for 10 min. Two 100 µL aliquots of this culture were each combined with 1 µg of linearized plasmid (digested with SwaI and gel-purified). Each mixture was transferred to a pre-chilled 100 µl Single Nucleocuvette (Lonza) and subjected to electroporation using a 4D-Nucleofector (Lonza) with the FI-115 program. Afterward, the cuvettes were returned to ice for 10 min. The cells from both cuvettes were combined and then allowed to recover overnight in fresh BHI-hemin medium under static suspension conditions. Following recovery, the cells were pelleted by centrifugation at 2000 × *g* for 3 min, resuspended in 200 µL of medium, and plated onto BHI agar containing 100 µg/mL hygromycin-B. Plates were incubated for 4–5 days until colonies appeared. Individual colonies were transferred into 150 µL of BHI-hemin medium in a 96-well plate and subsequently genotyped to confirm the integration of CDC5L-GFP into the genome (Supplementary Fig. 1b). Extraction of *L. tarentolae* genomic DNA was carried out using the QuickExtract DNA Extraction Solution (Lucigen, QE09050) following the standard protocol. The following PCR primers were used to detect the integration of CDC5L into an rDNA locus: PCR1 (F3001, A1715), PCR2 (P1442, A264) (Supplementary Table 3). Finally, nuclear localization of CDC5L was confirmed by monitoring the GPF signal using an epi-fluorescence microscope (Supplementary Fig. 1c).

### Purification of endogenous trans-spliceosomes from *L. tarentolae* cells

The purification of *L. tarentolae* trans-splicing intermediates was performed through sequential fractionation (Supplementary Fig. 1d). Five liters of CDC5L-GFP^+^ *L. tarentolae* cells were cultured in suspension in Yeast Extract medium (24 g/L) supplemented with 1% heat-inactivated FBS (ThermoFisher Scientific), 0.0005% hemin (Sigma), and PenStrep (50 U penicillin, 50 µg/mL streptomycin) (Sigma). Cells were collected at an OD of 2.5, representing the mid-logarithmic growth phase, washed twice with ice-cold freezing buffer (50 mM Hepes-NaOH, pH 7.8, 150 mM NaCl, 5% v/v glycerol, 0.5 mM PMSF, 1 mM pepstatin, 1 mM E-64 protease inhibitor, 1 mM DTT), and frozen in liquid nitrogen as droplets for storage at −80 °C. Frozen cell pellets were resuspended in Buffer A (50 mM Hepes, pH 7.6, 130 mM KCl, 3 mM MgCl_2_, 1 mM EDTA, 10% v/v glycerol, 0.2% Triton-X, 1 mM DTT, 0.5 mM PMSF, 1 mM pepstatin, 1 mM E-64). The suspension was homogenized with 10 strokes of a dounce homogenizer (pestle B) on ice. The lysate was centrifuged, and the supernatant was discarded. The resulting pellet was washed twice with Buffer A, and intact nuclei were resuspended in Buffer B (25 mM Hepes, pH 7.6, 10 mM KCl, 5 mM MgCl_2_, 1 mM CaCl_2_, 0.25 mM EDTA, 100 mM arginine, 1 mM spermidine, 5% v/v glycerol, 0.5% Triton, 1 mM DTT, 0.5 mM PMSF, 1 mM pepstatin, 1 mM E-64 protease inhibitor, 800 U RNase-free DNase I). The suspension was incubated on a nutator at 4 °C for 30 min. The insoluble fraction was then removed via centrifugation at 25,000 × *g* for 20 min at 4 °C. The supernatant was incubated with anti-GFP nanobody beads (Chromotek) for 4 h at 4 °C. The beads were then washed twice with Wash Buffer 1 (25 mM Hepes, pH 7.6, 150 mM KCl, 3 mM MgCl_2_, 0.25 mM EDTA, 100 mM arginine, 5% v/v glycerol, 0.1% Triton, 1 mM DTT, 0.5 mM PMSF, 1 mM pepstatin, 1 mM E-64) and once with Wash Buffer 2 (20 mM Hepes, pH 7.6, 100 mM KCl, 2.5 mM MgCl_2_, 0.05% C12E8 (Anatrace, O330)). Elution was achieved by 3C-protease cleavage. The eluted complexes were centrifuged at 25,000 ×  *g* for 30 min to remove aggregates and were immediately used for cryo-EM grid preparation or analyzed by SDS-PAGE, northern blot and mass spectrometry (LC-MS/MS) (Supplementary Fig. 1e–g, and Supplementary Data 1).

### RNA extraction and Northern Blot analysis

The RNA was extracted using Trizol/chloroform and isopropanol precipitation following standard procedures. The purified RNA was loaded multiple times (100 ng in each well) and resolved on a 10% TBE-Urea-PAGE. After complete migration, the RNA in one well was stained with Sybr Green II and the rest was transferred to a positively charged nylon membrane and fixed by UV-crosslinking. 5’-biotin-labeled oligonucleotide probes were used for Northern blot analysis using the Chemiluminescent Nucleic Acid Detection Module Kit (Thermo Fischer Scientific 89880) based on chemiluminescence detection (Supplementary Fig. 1f and Supplementary Table 3). The Century RNA marker (Thermo Fischer Scientific) was used as a molecular weight ladder.

### In silico interactome

A set of 64 proteins was selected based on the most detected proteins (area peak) in the mass spectrometry (MS) analysis of immunoprecipitated endogenously tagged trans-spliceosomal complexes. This selection includes 52 proteins that are identified and built in our cryo-EM reconstruction. The remaining 12 proteins were selected based on their potential role in pre-mRNA splicing or RNA binding. In silico modeling of protein-protein interactions was performed using AlphaFold-multimer^24^ to predict the structural interactome of the 64 identified proteins. The sequences of the selected proteins were retrieved from Uniprot or TriTryp databases^61,62^. All-against-all pairwise combinations of the 64 proteins were submitted to AlphaFold Multimer to predict potential interaction interfaces. Each protein pair was modeled with five independent runs to account for stochastic variations in structure prediction. Predicted protein-protein interactions were ranked based on the AlphaFold-multimer iPTM+pTM score (0.8·ipTM + 0.2·pTM), averaged across five runs. This composite score reflects both the confidence in individual domain structures and the reliability of the predicted interaction interface. All AlphaFold-multimer calculations were run on a high-performance computing cluster equipped with NVIDIA A100 GPUs. UniProt identifiers are provided in Supplementary Data 1.

### Pairwise alignments

The sequences of all proteins identified in our cryo-EM reconstruction were retrieved from the UniProt and TriTryp databases, along with their homologs from human (*Homo sapiens*), yeast (*Saccharomyces cerevisiae*), worm (*C. elegans*), and algae (*C. reinhardtii*). Protein pairs were globally aligned using EMBOSS-Needle^63,64^ in an all-against-*Leishmania* approach. Identity percentages were then used to construct an identity matrix. Source data are provided as a Source Data file.

### Cryo-EM grid preparation

Cryo-EM grids were prepared using a Vitrobot Mark IV (FEI Company) under controlled conditions of 10 °C and 95% humidity. A 3.5 µL sample was applied twice to glow-discharged Quantifoil R3.5/1 grids coated with a 2 nm ultrathin carbon layer (Au 400 2 nm C, Electron Microscopy Sciences). Each application was followed by a 30-s incubation, with the first application manually blotted. After the second incubation, the grid was blotted by the Vitrobot for 15 s with a blot force of 8 before being plunged into liquid ethane.

### Cryo-EM data collection

The grids were imaged using a 300 keV Titan Krios (FEI) equipped with an energy filter (slit width 20 eV) and a K3 Summit direct electron detector (Gatan) at a super-resolution pixel size of 0.54 Å/px. Using SerialEM^65^, two datasets, totaling 31,524 movies with 40 subframes, were collected with a total electron dose of 50 e⁻/Å² at a rate of 30 e⁻/px/sec in super-resolution mode, with defocus values ranging from −0.5 to −2.5 µm. A multi-shot 3 × 3 beam-tilt imaging strategy was used to acquire 99 micrographs per stage position (11 shots per hole) and 72 micrographs per stage position (8 shots per hole) for dataset 1 and dataset 2, respectively.

### Cryo-EM data processing

All data processing was carried out in cryoSPARC v4.6.0^66,67^, except for 3D classification without alignment which was carried out with RELION5^68,69^ (Supplementary Figs. 2–4). Raw movies were on-the-fly gain-corrected, dose-weighted, aligned and binned to a pixel size of 1.08 Å using cryoSPARC Live. During import, movies were pooled in nine different optic groups for each dataset. The defocus value of each micrograph was estimated using Patch CTF Estimation. A total of 4,721,671 particles were picked from two datasets using the Blob Picker. Particles from each dataset were extracted at a pixel size of 4.32 Å and initially cleaned using 2D classification. For dataset 1, ab-initio reconstruction was performed with two classes and standard parameters, except that the maximum resolution was set to 10 Å. The resulting ab-initio models included one “good” class resembling a trans-spliceosome and one “bad” class containing “junk” or low quality particles. These models were then used as initial references for both datasets (1x good class and 5x bad class) for further cleaning through heterogeneous refinement (Data cleanup 1). For dataset 1, an additional round of 3D classification without alignment (T-value = 4) was performed to remove particles contributing to anisotropic reconstructions (Data cleanup 2). For each dataset, after eliminating as many “junk” particles as possible, the particles contributing to the reconstruction of a defined trans-spliceosome were selected, re-extracted at a pixel size of 1.08 Å and merged into a stack of 571,611 particles. These particles were subjected to one round of Global and Local CTF refinements and Reference Based Motion Correction. The resulting polished particles stack was 3D refined, yielding a consensus trans-spliceosomal complex at a global resolution of 2.67 Å. To resolve distinct structural states within the consensus reconstruction, a global 3D classification without alignment was performed (T-value = 4). This analysis identified two major trans-splicing intermediates: a trans*-*C* complex and a trans*-*P complex (Supplementary Fig. 2). In the trans-P complex, the absence of the EJC correlated with changes in the catalytic sites where the SL exon is ligated to the pre-mRNA. After calibration, the pixel size was adjusted to 1.065 Å/pix and the resolution of the reconstructions was recalculated. The global resolution for the two states is 2.69 Å and 2.83 Å at FSC-0.143, respectively.

To mitigate the flexibility observed in each state and search for sub-states, 3D classification, 3D variability, particle signal subtraction, and focused local refinements were performed in cryoSPARC using various masks targeting relevant modules (Supplementary Fig. 3 and Supplementary Table 1). During this analysis, we identified two conformations for PRP22/DHX8, either bound to RNA or open, which are found in both trans*-*C* and trans*-*P complexes. Each focused map, along with its respective half-maps, was rescaled to 1.065 Å/px and postprocessed in cryoSPARC. To enhance model building and overall visualization, the focused maps, as well as lowpass-filtered regions corresponding to the highly flexible SL Sm ring and BRR2 helicase, were combined into a composite map using the ‘vop max’ command in ChimeraX^70^ (Supplementary Fig. 5). Local resolution estimation for both the overall and focused maps was performed in cryoSPARC at FSC-0.143. Additionally, directional FSC curves were calculated using the Orientation Diagnostic tool in cryoSPARC, based on the 3DFSC method^71,72^ (Supplementary Fig. 4).

### Model building and refinement

A combination of AlphaFold structure predictions^24,73,74^ and de novo model building was used to build the different trans-splicing intermediates (Supplementary Table 2). All proteins were built and manually adjusted in COOT^75^ using an AlphaFold structure prediction as a starting model. The snRNAs and the pre-mRNA were manually built de novo in COOT and then rebuilt with optimized geometry using ERRASER^76^. BRR2 helicase and the SL Sm ring were rigid-body fitted into their corresponding lowpass–filtered densities using the fit_search.py script from the ChimeraX Recipes collection within ChimeraX^70^ (https://rbvi.github.io/chimerax-recipes/fit_search/fit_search.html). The search was performed with the following parameters: resolution 25, search 50, and cutoff 0.9. For BRR2, among the resulting placements with a cross-correlation above 0.9, we selected the fit (0.912) most consistent with the previously established position and orientation of BRR2 in the human P complex^23^. For the SL Sm ring, among the placements with a cross-correlation above 0.9, we selected the fit (0.953) that best satisfied the expected SL RNA connectivity.

The final models for the two states were real-space refined in their respective composite maps using phenix.real_space_refine in PHENIX 1.19.1^77^ with secondary structure restraints for proteins and RNAs. The half-map cross-validation procedure^78,79^ was used to assess potential overfitting during refinement. Briefly, the final initial models were refined against one half-map using the same parameters as for the full map, and FSC curves were then calculated separately against half-map A (FSC_work) and half-map B (FSC_test). The near-complete overlap of FSC_work and FSC_test indicates that no detectable overfitting occurred (Supplementary Fig. 4c). Atomic models for the two conformations of PRP22/DHX8 were also built in their respective focused maps. The complete list of proteins built in the structures can be found in Supplementary Fig. 8 and Supplementary Table 2. The model refinement statistics can be found in Supplementary Table 5. The maps and models were analyzed and visualized in ChimeraX^70^ or PyMol (Schrödinger, LLC). Figures were generated using ChimeraX.

### Generation of *L. tarentolae* cell line expressing GFP-PRP22^FL^ or GFP-PRP22^∆NTD^

To constitutively express GFP-PRP22^FL^ and GFP-PRP22^∆NTD^ in *L. tarentolae* from a genome-integrated position, the genes coding for *L. tarentolae* PRP22^FL^ (1-1087) and for PRP22^∆NTD^ (408-1087) were amplified by PCR (see used primers in Supplementary Table 4) and separately cloned into an in-house modified pLEXSY-hyg2.1 vector (Jena Bioscience) harboring a N-terminal dual affinity tag (TEV-cleavable GFP, 3C-cleavable HA) (Supplementary Fig. 20a). For transfection, the density of *L. tarentolae* P10 cells (Jena Bioscience) was adjusted to 10^8^ cells/mL in the P3 Primary Cell buffer (Lonza) and incubated on ice for 10 min. Two 450 µL aliquots of this culture were separately combined with 1 µg of each linearized plasmid (digested with SwaI and gel-purified). The mixtures were individually transferred to a pre-chilled 500 µL 4-mm single cuvette (Biorad) and subjected to electroporation using a Gene Pulser Xcell Total System (Biorad) with a high voltage program (1500 V, 25 µF, 2 pulses at 10 s interval). Afterward, the cuvettes were returned to ice for 10 min. The cells were allowed to recover overnight in fresh BHI-hemin medium under static suspension conditions. Following recovery, the cells for each construct were pelleted by centrifugation at 2000 × *g* for 3 min, resuspended in 200 µL of medium, and plated onto BHI agar containing 100 µg/mL hygromycin-B. Plates were incubated for 4–5 days until colonies appeared. Individual colonies were transferred into 150 µL of BHI-hemin medium in a 96-well plate and subsequently genotyped to confirm the integration of PRP22^FL^ or PRP22^∆NTD^ into the genome (Supplementary Fig. 20b). Extraction of *L. tarentolae* genomic DNA was carried out using the QuickExtract DNA Extraction Solution (Lucigen, QE09050) following the standard protocol. The following PCR primers were used to detect the integration of GFP-PRP22^FL^ or GFP-PRP22^∆NTD^ into an rDNA locus: PCR1 (F3001, A1715), PCR2 (P1442, A264) (Supplementary Table 3). Finally, nuclear localization of GFP-PRP22^FL^ or GFP-PRP22^∆NTD^ was confirmed by monitoring the GPF signal using an epi-fluorescence microscope (Supplementary Fig. 20c).

### Two-step purification of PRP22^FL^ or PRP22^∆NTD^

Purification of PRP22^FL^ and PRP22^ΔNTD^ was performed using an identical workflow. Six liters of GFP-PRP22^FL+^ or GFP-PRP22^∆NTD+^ *L. tarentolae* cells were cultured in suspension in Yeast Extract medium (24 g/L) supplemented with 1% heat-inactivated FBS (ThermoFisher Scientific), 0.0005% w/v hemin (Sigma), and PenStrep (50 U penicillin, 50 µg/mL streptomycin) (Sigma). Cells were collected at an OD of 2.5, representing the mid-logarithmic growth phase, washed twice with ice-cold freezing buffer (50 mM Hepes-NaOH, pH 7.8, 150 mM NaCl, 5% v/v glycerol, 0.5 mM PMSF, 1 mM pepstatin, 1 mM E-64 protease inhibitor, 1 mM DTT), and frozen in liquid nitrogen as droplets for storage at −80 °C. Frozen cell pellets were resuspended in Lysis buffer (50 mM Hepes, pH 7.8, 130 mM KCl, 370 mM NaCl, 2 mM MgCl_2_, 1 mM EDTA, 10% v/v glycerol, 0.1% Triton-X, 1 mM DTT, 0.5 mM PMSF, 1 mM pepstatin, 1 mM E-64, 200 U of benzonaze) and lysed with three cycles of high-pressure disruption using an EmulsiFlex-C3 at 1500 bars. Insoluble material was removed via centrifugation at 25,000 × *g* for 30 min at 4 °C. The clarified lysate was incubated with anti-GFP nanobody beads (Chromotek) for 4 h at 4 °C. The beads were washed twice with Wash Buffer A (50 mM Hepes, pH 7.8, 130 mM KCl, 1 M NaCl, 1 mM EDTA, 10% v/v glycerol, 1 mM DTT, 0.5 mM PMSF, 1 mM pepstatin, 1 mM E-64) and twice with Wash Buffer B (25 mM Hepes, pH 7.8, 130 mM KCl, 370 mM NaCl, 10% v/v glycerol, 0.05% C12E8 (Anatrace, O330)). Bound proteins were eluted by TEV protease cleavage for 2 h. The eluates were clarified by centrifugation at 25,000 × *g* for 15 min to remove potential aggregates and were immediately analyzed by SDS-PAGE (Supplementary Fig. 20f). Yields of 0.3 mg (PRP22^FL^) and 1 mg (PRP22^ΔNTD^) were obtained from 12 L of culture. Integrity of the purified proteins was assessed by Western blot (Supplementary Fig. 20g). As both constructs were found to co-purify with HSP70 (identified by in-gel mass spectrometry), a second purification step was performed using the N-terminal HA tag. The TEV eluates were incubated with anti-HA magnetic beads (Pierce) for 2 h at 4 °C. Beads were washed five times with Wash Buffer A supplemented with 25 mM ATP and 2 mM MgCl_2_, followed by two washes with Wash Buffer A containing 1 M NaCl to remove ATP-dependent chaperones. Proteins were eluted by 3 C protease cleavage for 2 h. The eluates were centrifuged at 25,000 × *g* for 15 min and analyzed by SDS-PAGE (Supplementary Fig. 20i). Exact protein concentrations were determined by densitometric comparison with a known amount of purified 3 C protease. Final yields of 7 µg (PRP22^FL^) and 4 µg (PRP22^ΔNTD^) were obtained.

### Probable ATPase (LtaP16.0490) expression and purification

The sequence coding for the probable helicase LtaP16.0490 (Genome LtarentolaeParrotTarII) harbors many insertion loops in RecA1 domain. To increase the expression and solubility of the protein, these insertions were trimmed between residues 44-79, 165-224, 318-350 and 427-817. The resulting coding sequence was codon-optimized for *E. coli* and inserted in a pET28a between the NcoI/NotI restrictions site with a N-terminal 10His-SUMO tag. Overexpression was performed in *E*. *coli* BL21 (DE3) in LB medium containing 50 µg/mL kanamycin. Cells were induced with 1 mM IPTG after reaching an OD_600_ of 1 and protein was expressed at 18 °C for 16 h. Cells were harvested and resuspended in lysis buffer (50 mM Hepes pH 7.8, 500 mM NaCl, 2 mM MgCl_2_, 15 mM imidazole, 10% v/v glycerol, 0.1% Triton X-100, 1 mM DTT, 0.5 mM PMSF, 1 mM pepstatin, 1 mM E-64, 200U of benzonaze) and lysed with three cycles of high-pressure disruption using EmulsiFlex-C3 at 1500 bars. The insoluble fraction was then removed via centrifugation at 25,000 × *g* for 30 min at 4 °C. The supernatant was incubated with Ni Sepharose 6 Fast Flow (Cytiva) for 2 h at 4 °C on a nutator. The Sepharose beads were then washed three times with Lysis Buffer, three times with Wash Buffer A (50 mM Hepes, pH 7.8, 1 M NaCl, 15 mM imidazole, 10% v/v glycerol, 1 mM DTT) and three times with Wash Buffer B (30 mM Hepes, pH 7.8, 500 mM NaCl, 10% v/v glycerol, 1 mM DTT). Elution was achieved by incubating the Sepharose beads with 500 µg of Ulp1 protease in Wash Buffer B for 2 h at 4 °C under agitation. The elution procedure was then repeated twice. The three elutions were pooled, concentrated to 4 mL final volume and loaded on a Superdex S200 16/60 size-exclusion chromatography column (Cytiva) equilibrated with Gel filtration buffer (30 mM Hepes, pH 7.8, 500 mM NaCl, 10% v/v glycerol, 1 mM DTT). The peak with the least contaminant was selected for further characterization. Seven mg of LtaP16.0490 were obtained from 4 L of cultures (Supplementary Fig. 21g). Protein concentration was subsequently corrected for the presence of contaminating proteins by gel densitometry.

### Western blotting

Equal amounts of PRP22 purified constructs (200 ng) and equivalent amount of GFP control sample (200 ng of total protein) were separated by SDS–PAGE and transferred to a PVDF membrane (cat. #10600023, Amersham). The membrane was blocked in TBS-T supplemented with 4% BSA for 1 h at room temperature before incubating with mouse anti-HA primary antibody at 1:5000 dilution (cat. #H3663, Sigma) in TBS-T with 4% BSA for 1 h at room temperature. The membrane was washed three times in TBS-T and then incubated for 1 h at room temperature with the HRP-conjugated secondary antibody rabbit anti-mouse IgG H&L (cat. #ab6728, Abcam) diluted at 1:10000 in TBS-T with 4% BSA. The membrane was washed twice in TBS-T and once in TBS before imaging on a ImageQuant LAS4000 imager (GE) using Clarity Western ECL Substrate (cat. #1705060, Biorad). The Precision Plus Protein Dual Color Standards (cat. #1610374, Biorad) was used as a molecular weight ladder.

### ATPase activity assay

ATP hydrolysis was measured by monitoring the oxidation of NADH mediated by pyruvate kinase (PK) and lactate dehydrogenase (LDH). The absorbance was monitored at 340 nm over 900–3600 s at 26 °C with a Microplate Reader (Tecan infinite 200Pro). Reactions were recorded in technical triplicates with 125 nM of both PRP22^FL^ and PRP22^∆NTD^ in 100 µL of a buffer containing 20 mM Hepes pH 7.8, 250 mM NaCl, 20 mM MgCl_2_, 1 mM TCEP, 100 µg/mL of BSA, 4U/6U of PK/LDH mixture, 2 mM PEP, 0.5 mM NADH and saturating concentration of ATP. Absorbance values were normalized to a 1-cm path length by the instrument and converted to concentrations using the Beer-Lambert law, applying an extinction coefficient of 6,220 M^−1^ cm^−1^ at 340 nm. Initial velocities were determined by linear regression of the concentration vs. time plot. The rates were corrected by subtracting the background signal measured in the absence of ATP. Under saturating ATP concentrations (2 mM), turnover values (*K*_cat_) were calculated by normalizing the corrected initial rates to the enzyme concentration. Data analysis was performed using GraphPad Prism 11.0.0.

### Reporting summary

Further information on research design is available in the Nature Portfolio Reporting Summary linked to this article.

## Supplementary information

Supplementary Information

Supplementary Data 1

Supplementary Data 2

Supplementary Data 3

Supplementary Movie 1

Supplementary Movie 2

Reporting Summary

Transparent Peer Review file

## Source data

Source File

## Data Availability

Raw unaligned multi-frame movies and consensus polished particles stack have been deposited in the Electron Microscopy Public Image Archive (EMPIAR-13613). The composite cryo-EM maps and atomic models have been deposited in the Electron Microscopy Data Bank (EMDB) and the Protein Data Bank (PDB), respectively: trans-C* complex (EMD-52843 and PDB 9IF7), trans-P complex (EMD-52844 and PDB 9IF8), RNA-bound PRP22 in closed conformation (EMD-52808 and 9IBC) and PRP22 in open conformation (EMD-52809 and 9IBD). All overall and focused refinement maps have also been deposited in the EMDB (Supplementary Table 1). The following atomic models were used for structural comparison and figure generation: 6SH7, 7W5A, 8C6J, 9DTR and 9FMD. Source data are provided in this paper. Source data are provided with this paper.
