# Supplementary material for: Structural basis of step II spliced leader RNA trans-splicing in trypanosomatid parasites
Source: Nat Commun. 2026 Sep 23;17:9450. doi: 10.1038/s41467-026-77480-6 (PMC13601561; doi:10.1038/s41467-026-77480-6)
Supplement: Supplementary file 1 [file 41467_2026_77480_MOESM1_ESM.pdf]

Supplementary Information

Structural basis of step II Spliced Leader RNA trans-splicing in trypanosomatid parasites

Théo Nadenoen, Franco Agustin Biglione, Marylène Vandevenne, Arnaud Vanden Broeck*

Corresponding author: avbroeck@uliege.be

Supplementary Figures 1 to 21

Supplementary Tables 1 to 5

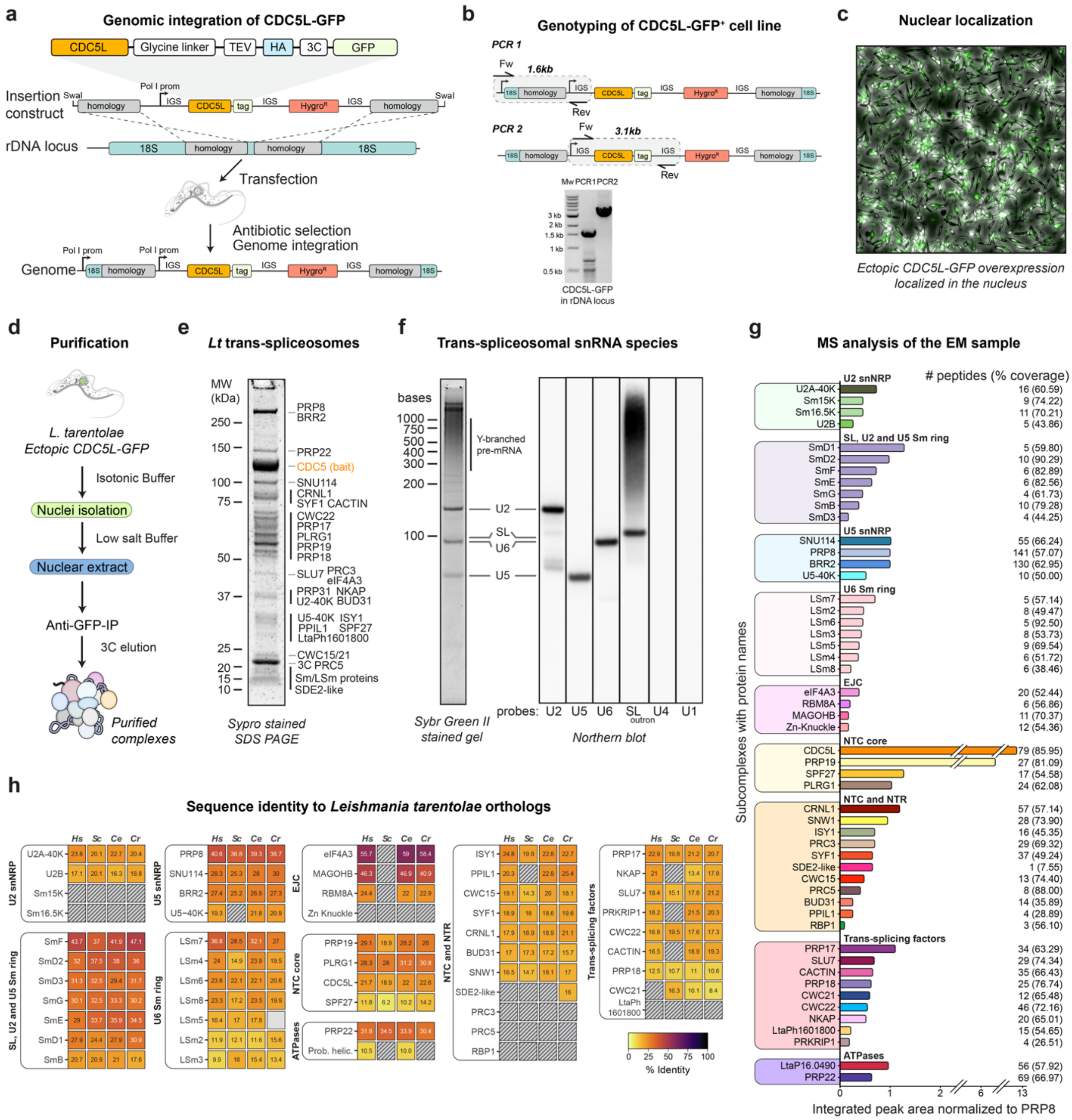

Supplementary Fig. 1. Tagging and purification of *Leishmania tarentolae* step II trans-spliceosomes.

a. Strategy for integrating an ectopic copy of affinity-tagged CDC5L into one of the rDNA loci in *Leishmania tarentolae* (*Lt*). **b.** Isolated single clones are genotyped to confirm the genomic integration of tagged CDC5L. In this case, two separate PCR reactions confirm the integration of the cassette in one of the rDNA loci. **c.** Fluorescence microscopy image of *Lt* CDC5L-GFP$^+$ cells showing a nuclear localization of the protein CDC5L. **d.** Workflow of the fractional lysis and affinity purification used to isolate CDC5L-containing trans-spliceosomal complexes. **e.** SYPRO Ruby stained SDS-PAGE analysis of the purified *Lt* trans-spliceosomes. Uncropped gel in Source Data. **f.** RNA staining and Northern blot analysis of snRNA species present in the purified *Lt* trans-

spliceosomes. Probes hybridizing within U2, U5, U6, SL_{outron}, U4 and U1 were used. Uncropped gel and blot in Source Data. **g.** Identification and abundance of proteins in the affinity-purified sample measured by mass spectrometry. The integrated peak areas were normalized to PRP8. Labels next to each bar show the number of peptides detected for each protein and the corresponding sequence coverage percentage. **h.** Sequence conservation analysis of spliceosomal proteins between *Leishmania tarentolae* (*Lt*) and human (*Hs*), *Saccharomyces cerevisiae* (*Sc*), *Caenorhabditis elegans* (*Ce*), and *Chlamydomonas reinhardtii* (*Cr*) reveals a markedly divergent protein composition in *Leishmania* compared to other model eukaryotes. Source data are provided as a Source Data file.

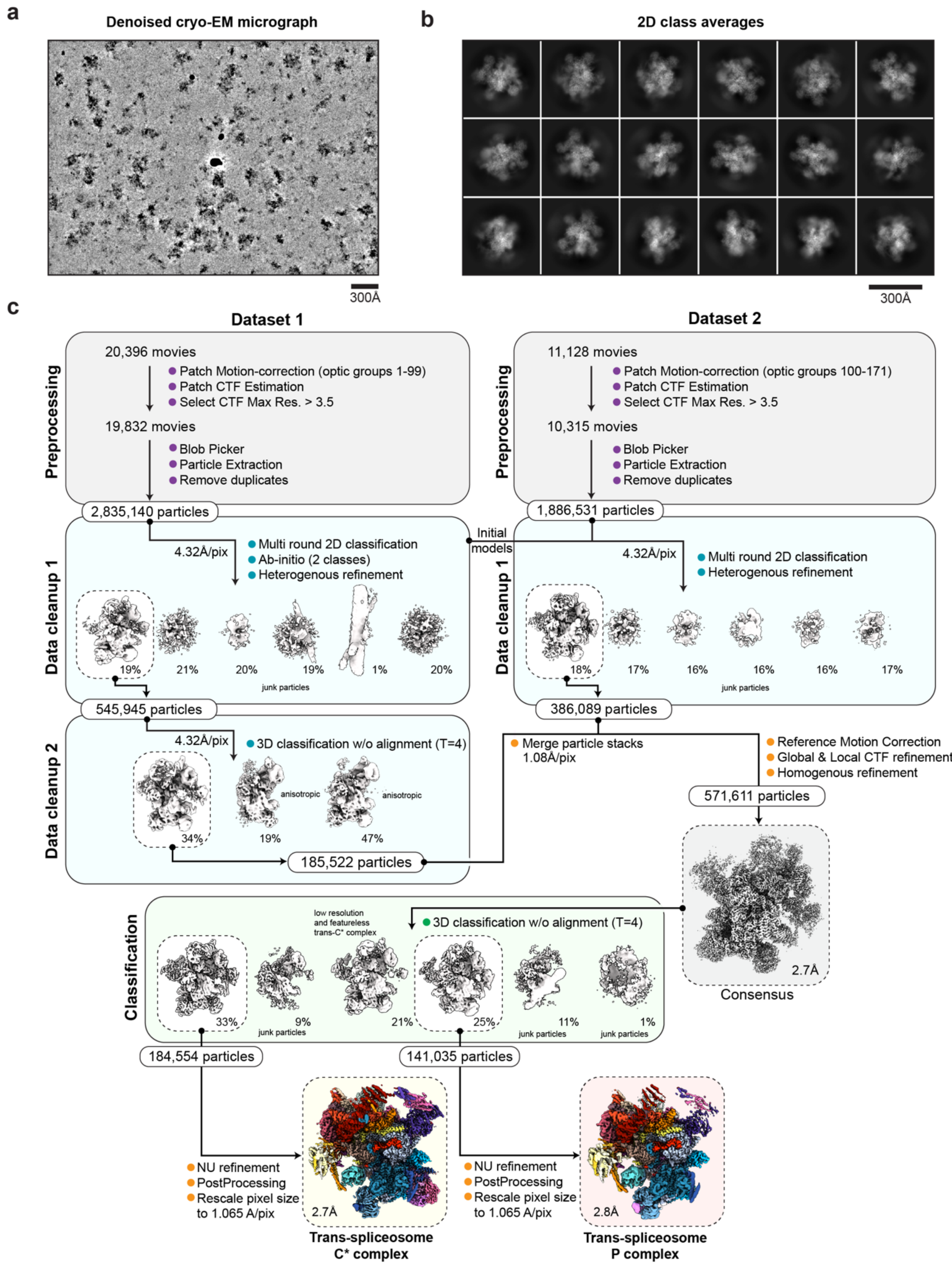

Supplementary Fig. 2. Representative cryo-EM data and image processing workflow.

a. Representative denoised cryo-EM micrograph. **b.** Cryo-EM 2D class averages. **c.** Cryo-EM data processing workflow for the determination of two step II trans-spliceosomal complexes (trans-C* and trans-P). Type of jobs are indicated. Map resolutions were determined at FSC-0.143.

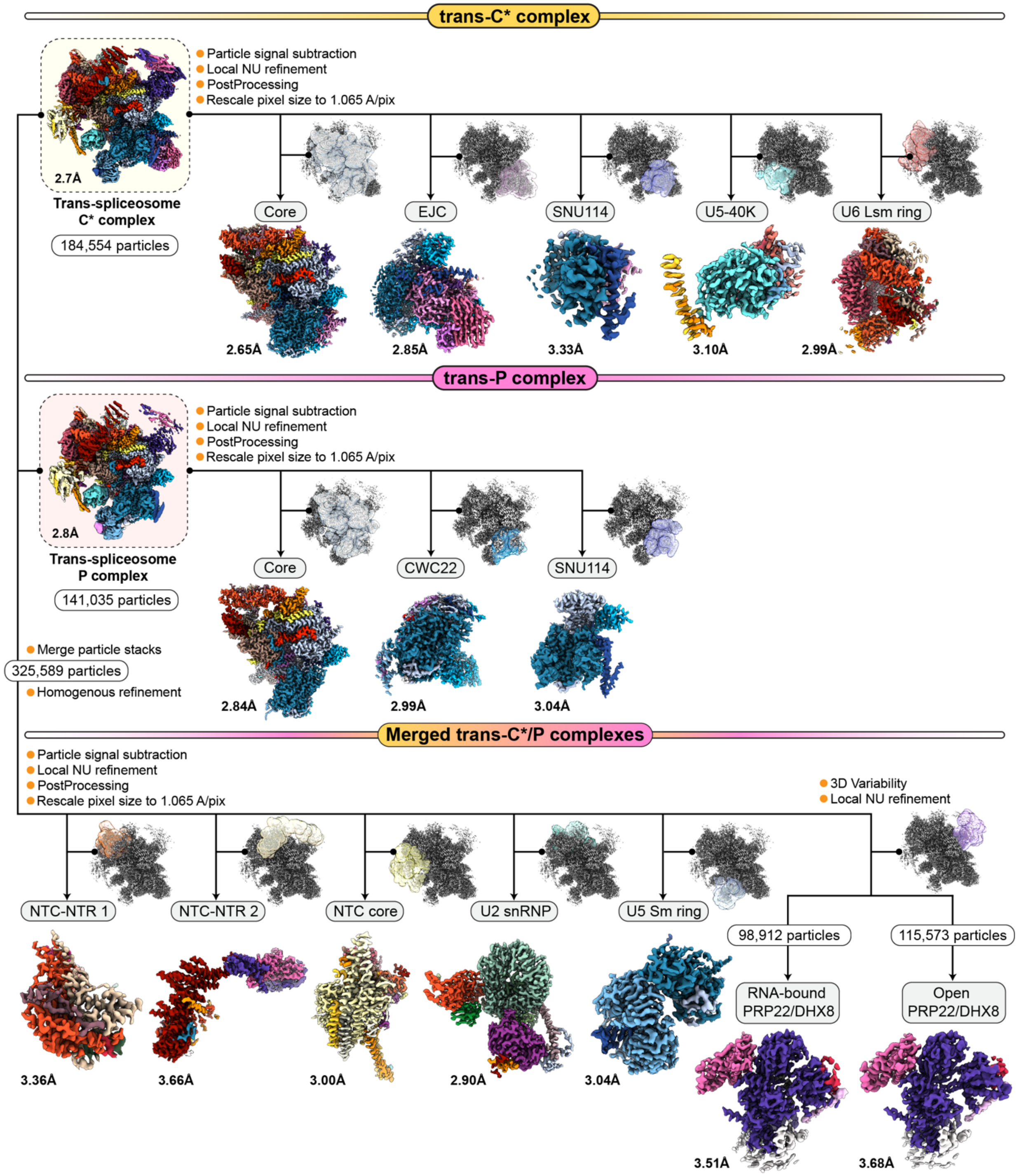

Supplementary Fig. 3. Focused refinements of the trans-C* and trans-P complexes.
Extensive focused refinements of each structural state yielded a total of 15 cryo-EM maps, providing detail of the trans-C* and trans-P complexes and enabling the construction of high-quality atomic models. Identical regions between the two states were combined during refinement to improve resolution.

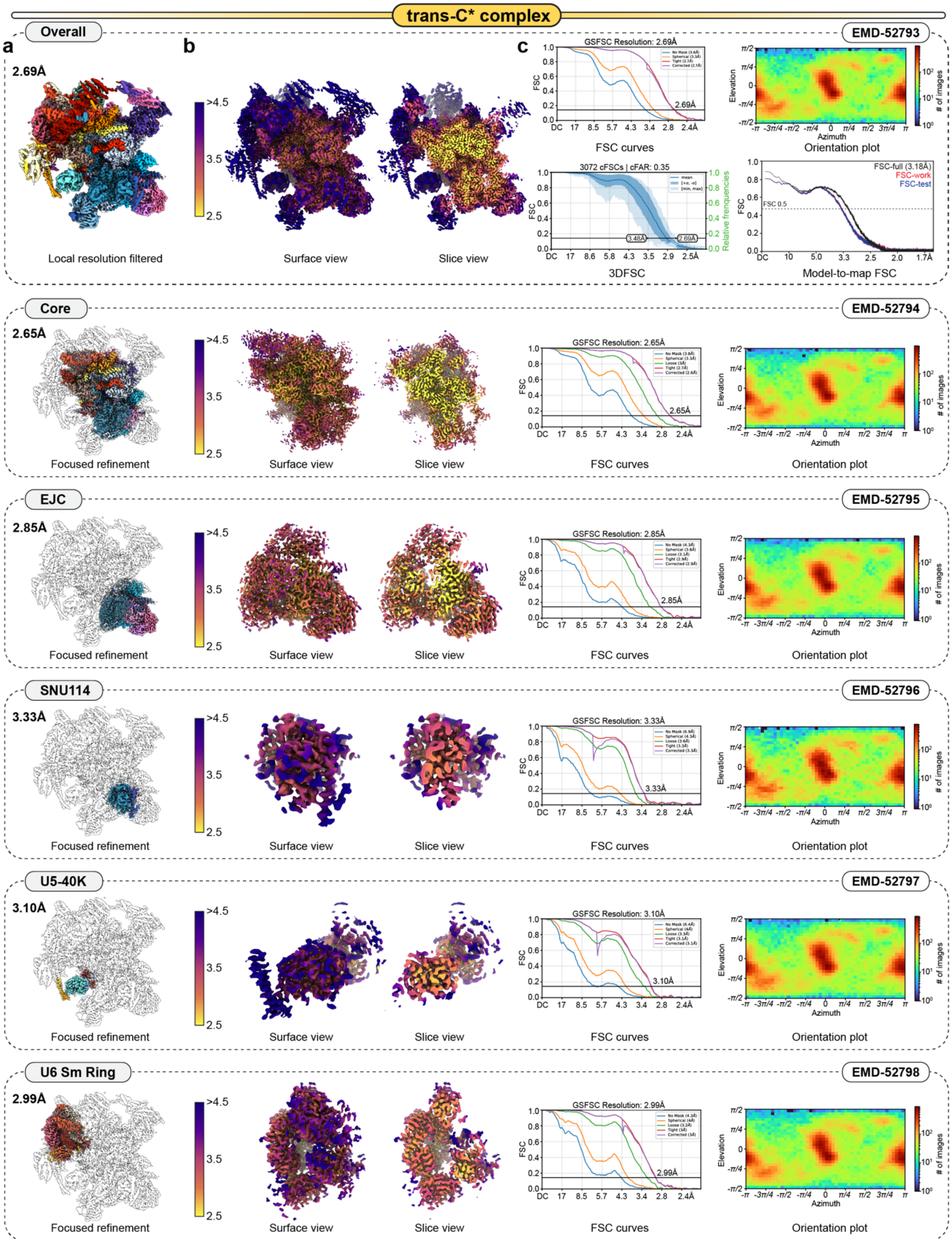

Figure continues on next page

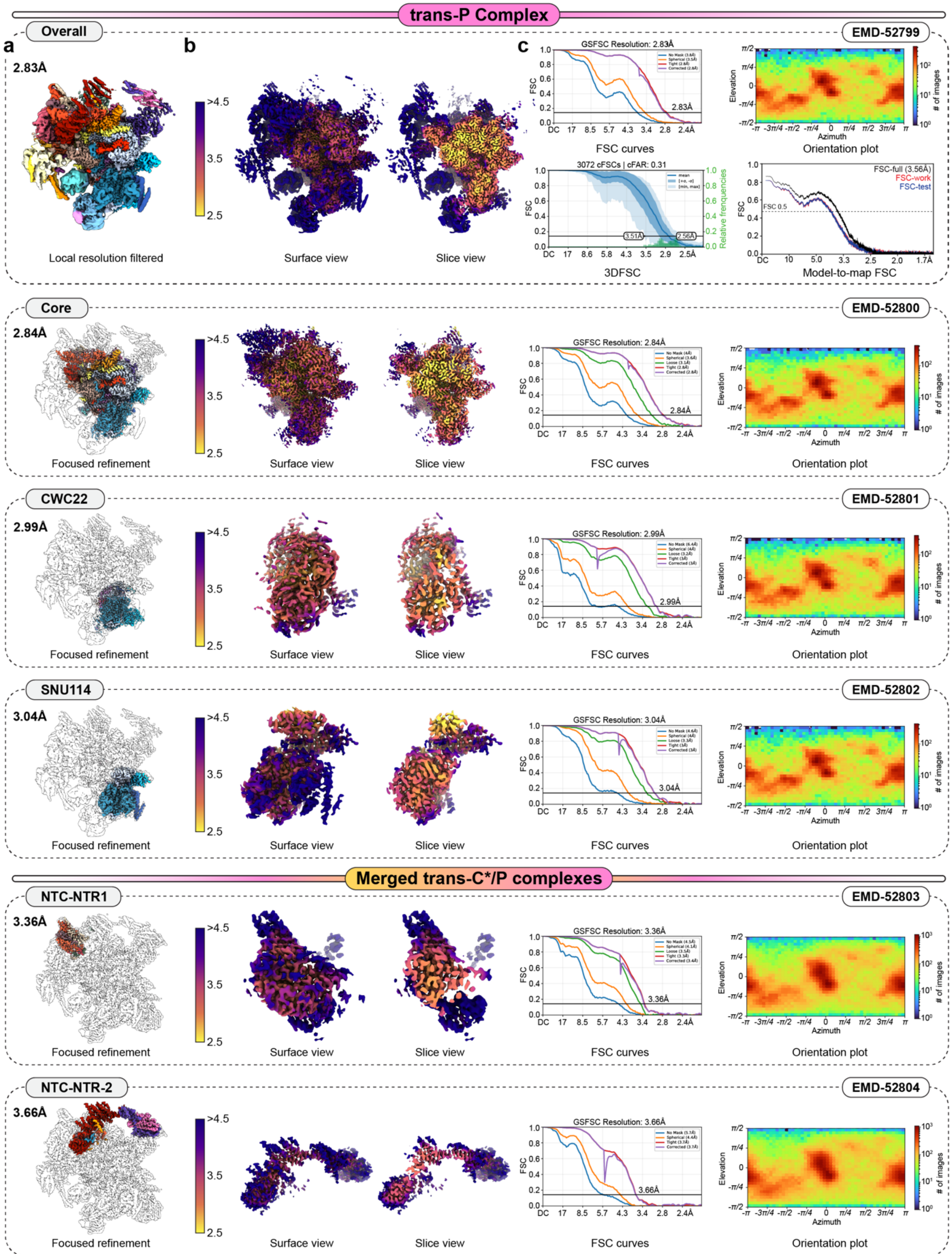

Figure continues on next page

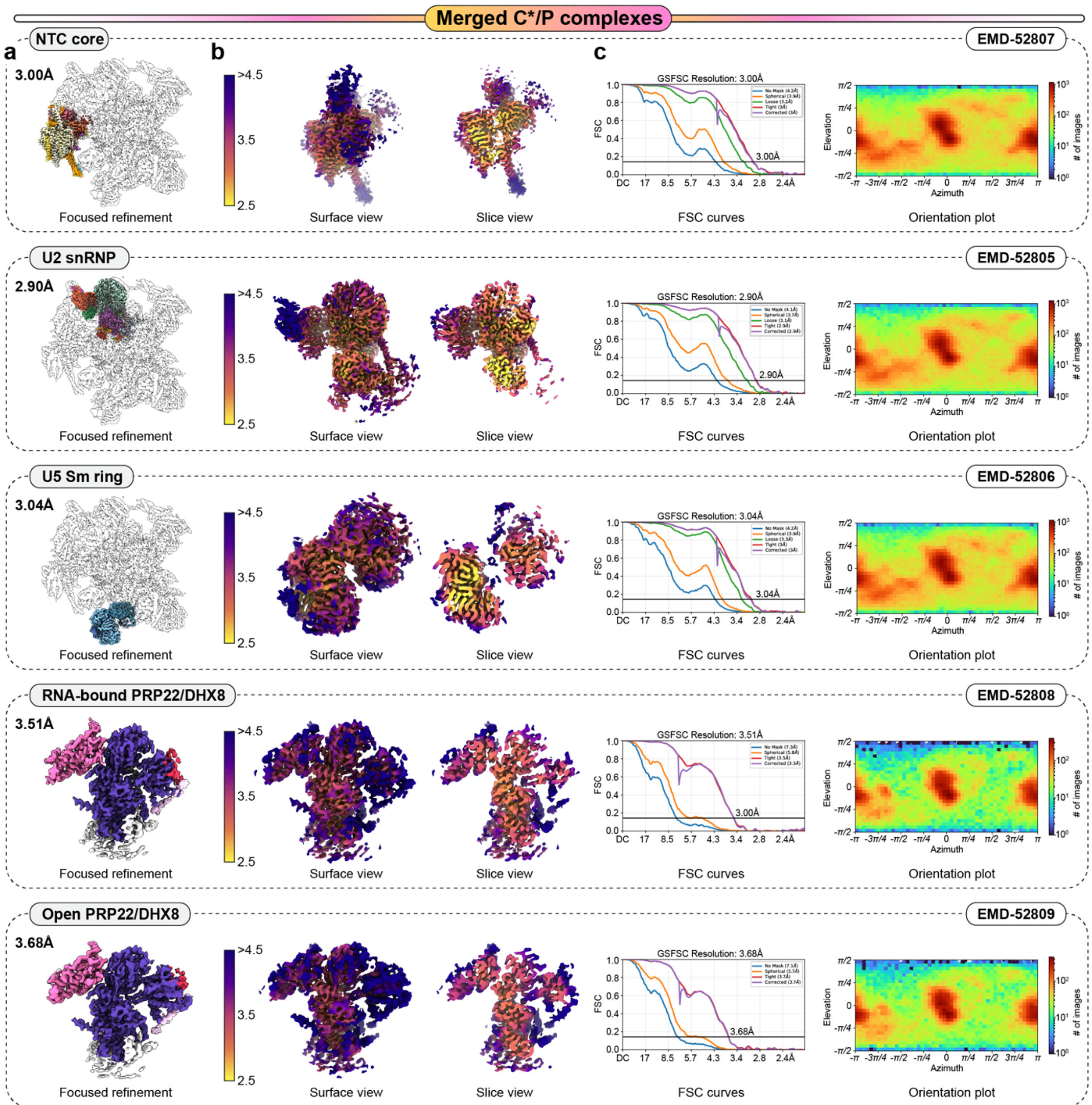

Supplementary Fig. 4. Global and local resolution estimation and particle distribution for overall complexes and focused refinements.

a. Overall or focused reconstruction. **b.** Local resolution estimation. **c.** Gold-standard FSC plots and 2D histograms depicting the Euler angles of particles. For the overall reconstructions, directional and model-to-map FSC plots are also provided in (**c**). The half-map cross-validation procedure [78,79] indicates no overfitting of the refined model, as FSC-work and FSC-test overlap. The resolution indicated for all FSC curves was determined at FSC-0.143, except for the map-to-model FSC which was determined at FSC-0.5. The Electron Microscopy Data Bank (EMDB) accession number is indicated for each map.

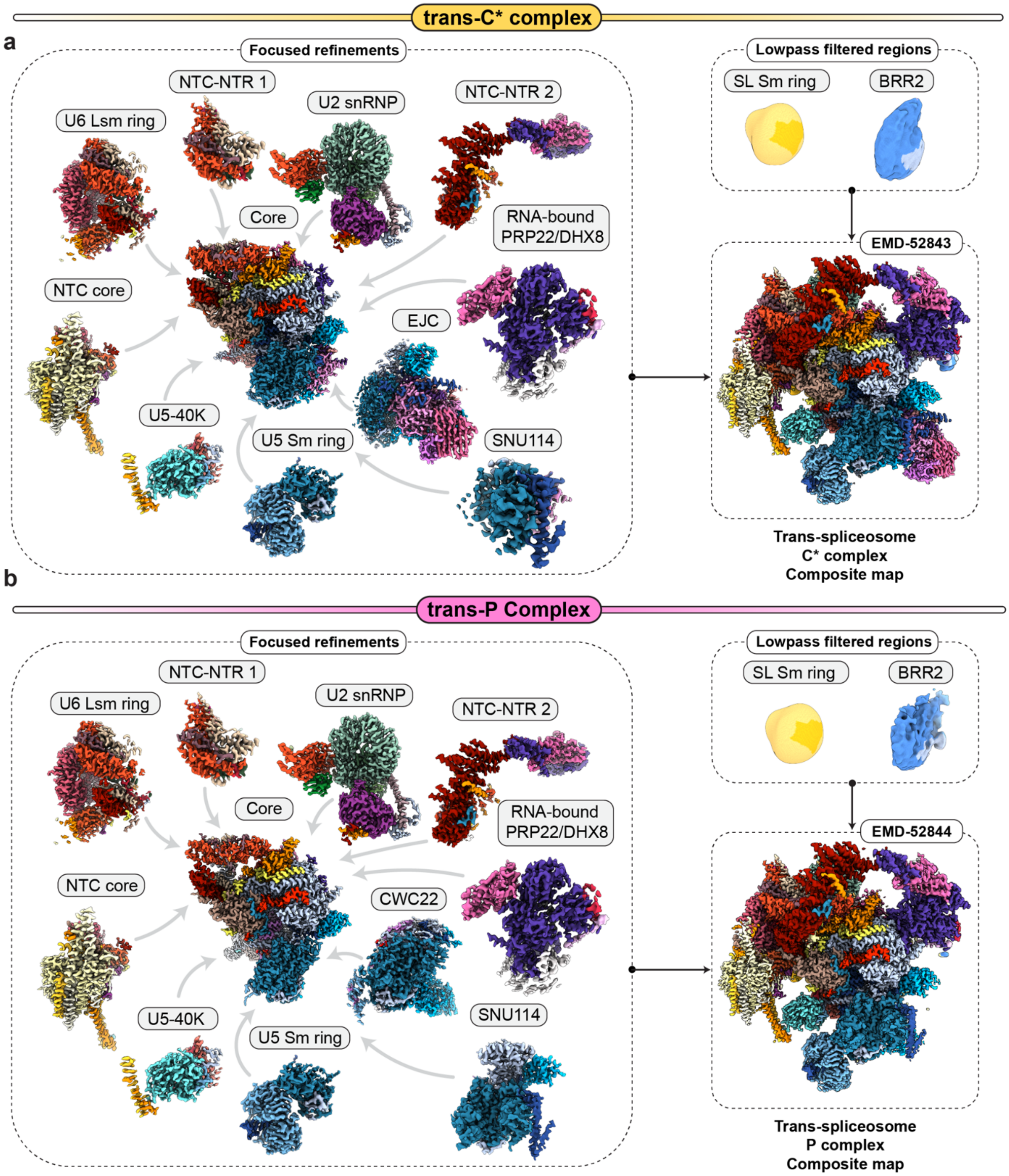

Supplementary Fig. 5. Composite cryo-EM reconstruction of trans-C* and trans-P complexes.

a. Focused refinement maps and low-pass filtered maps were assembled to generate a composite reconstruction of the trans-spliceosomal C* complex. **b.** Same as in (**a**), for the trans-spliceosomal P complex.

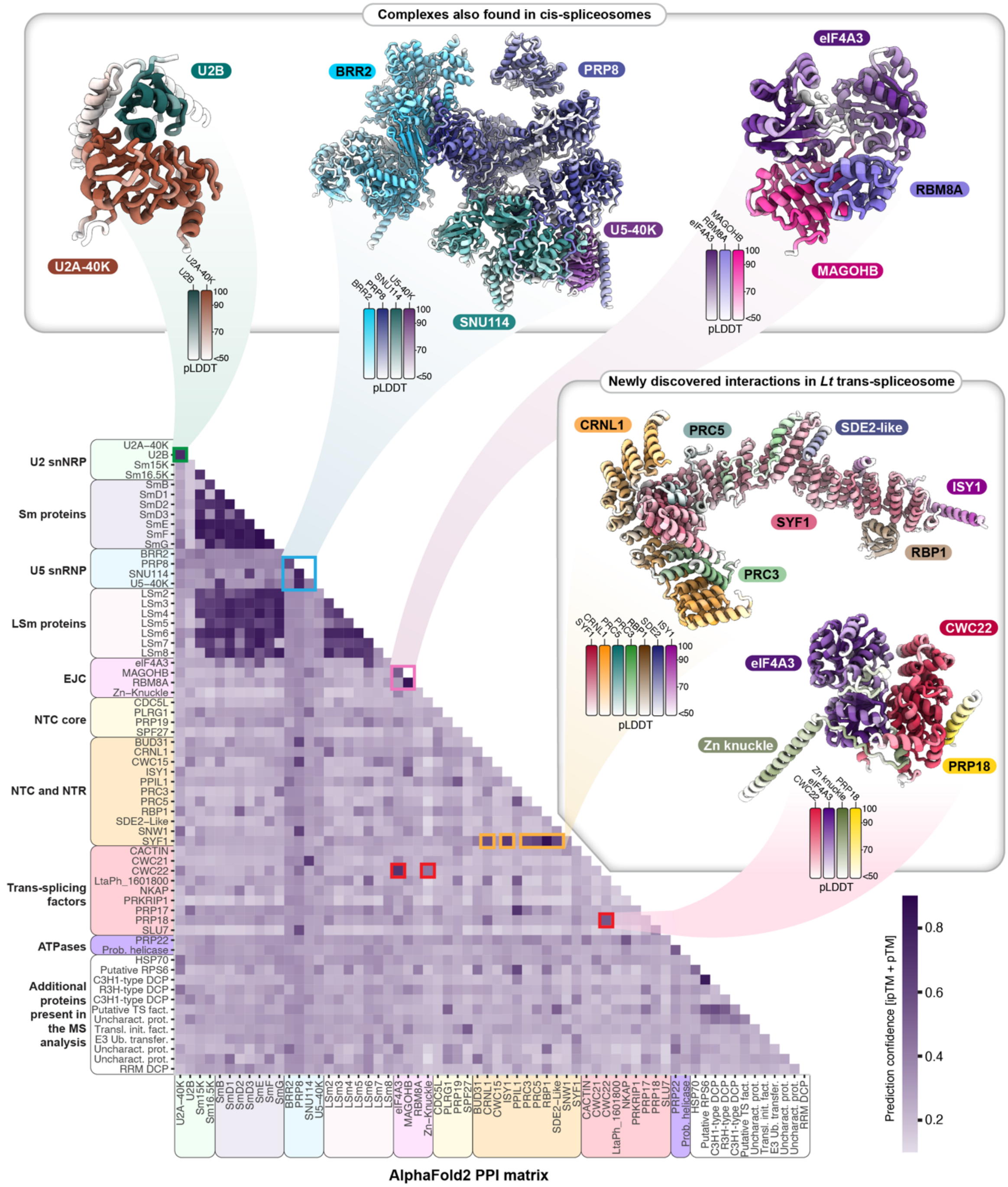

Supplementary Fig. 6. *In silico* interactome of *Leishmania tarentolae* SL trans-splicing machineries.

Heatmap of pairwise protein–protein interactions (PPIs) predicted using AlphaFold2-Multimer, colored by the ipTM+pTM interface confidence score. Representative structural models for five predicted complexes are shown, with proteins color-coded and confidence indicated by per-residue pLDDT scores. Models were trimmed to exclude regions with pLDDT < 50. The top three models correspond to interactions previously observed in canonical cis-spliceosomes [25-32], while the bottom two represent novel interactions specific to the *Lt* trans-spliceosome. Uniprot ID are

available in the Supplementary Data 1 (mass spectrometry analysis). The Source data are provided as a Source Data file.

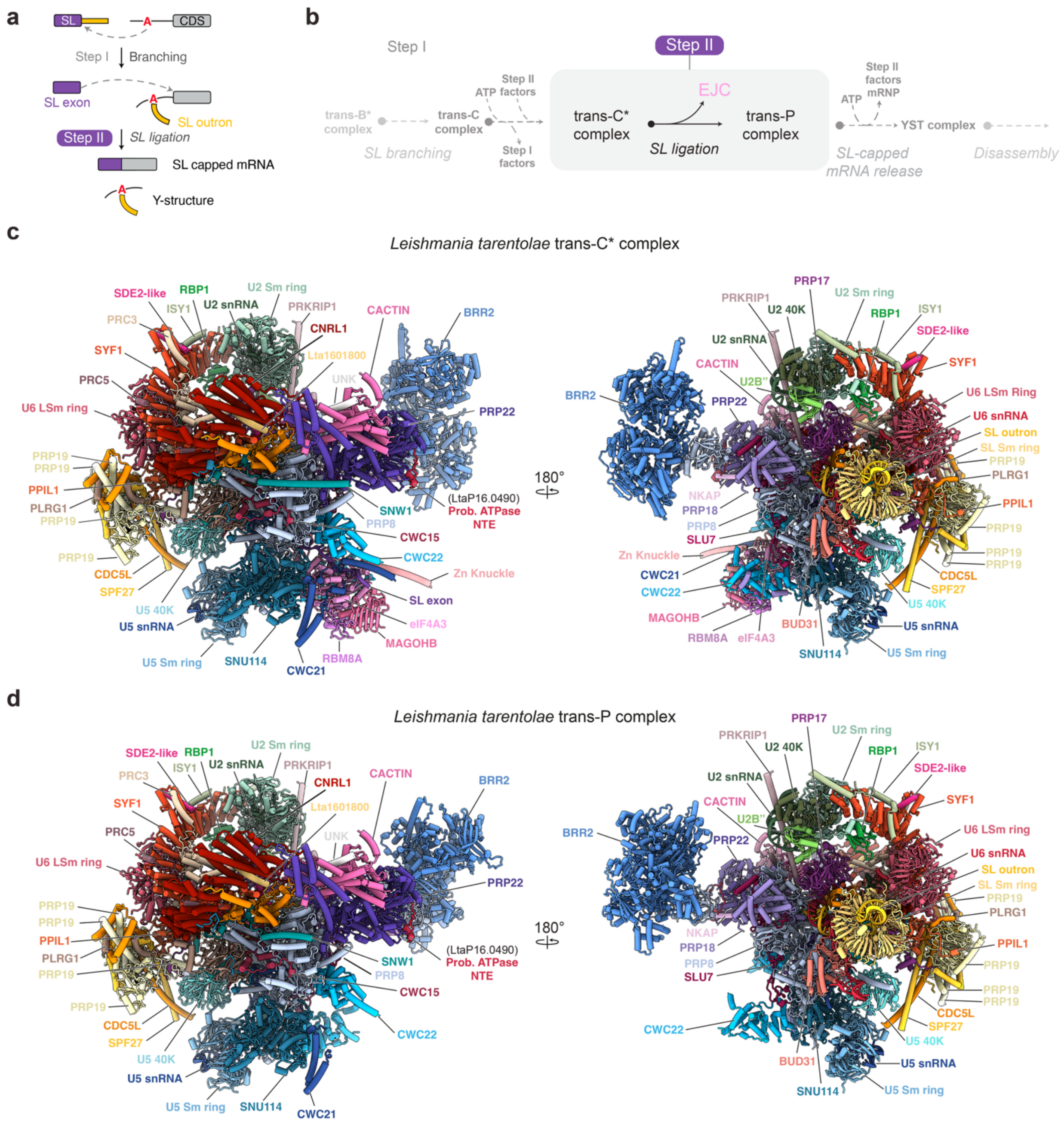

Supplementary Fig. 7. Cryo-EM structures of trypanosomatid step II trans-spliceosomes.

a. Schematic representation of the two-step intermolecular SL trans-splicing reaction. **b.** Detailed view of step I (SL branching) and step II (SL exon ligation) of SL trans-splicing. The trans-C* and trans-P complexes described in this study are indicated. **c-d.** Trypanosomatid trans-spliceosomal trans-C* and trans-P complexes flanking the second step of SL trans-splicing shown in 180° rotation with labeled protein and RNA components.

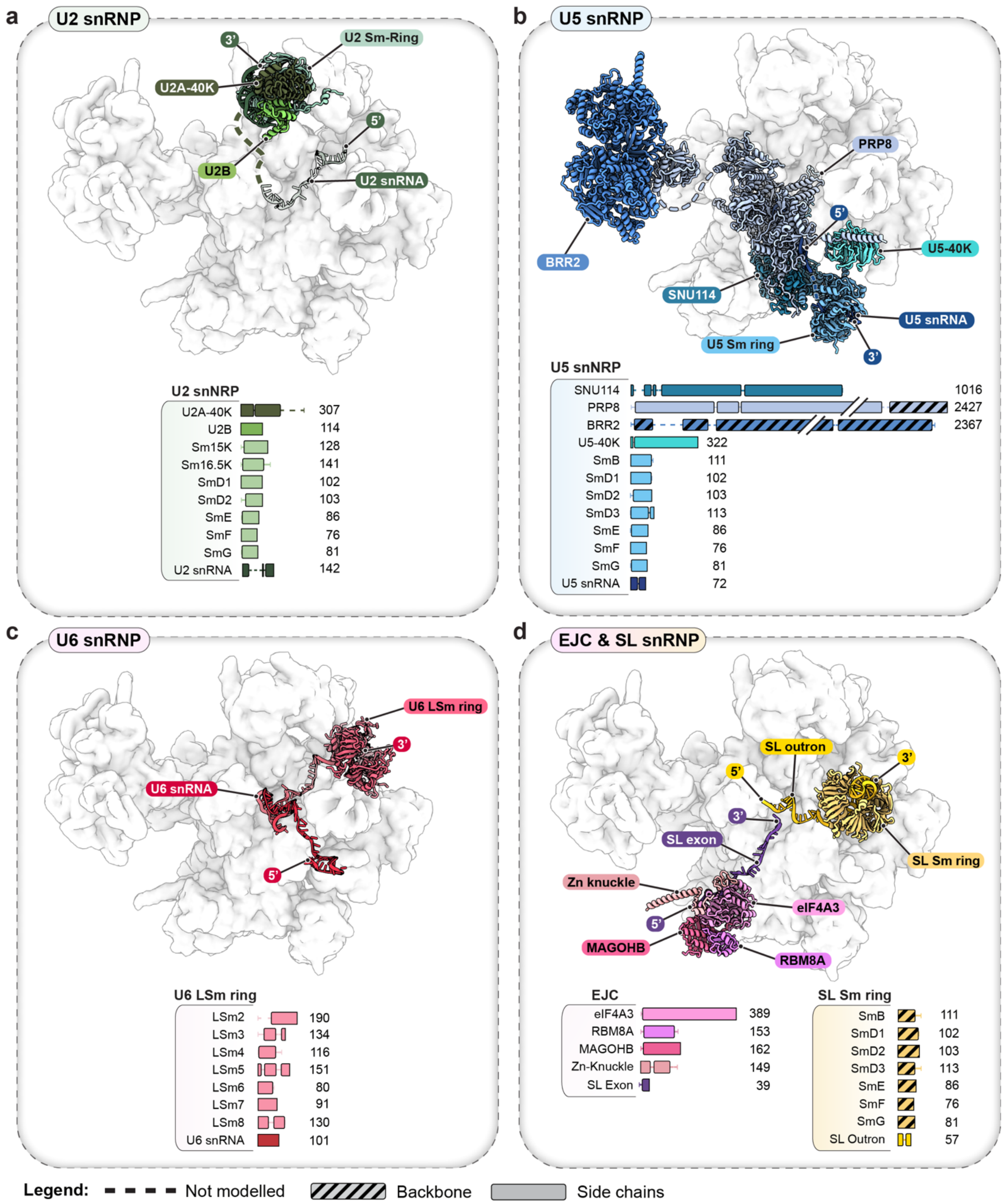

Figure continues on next page

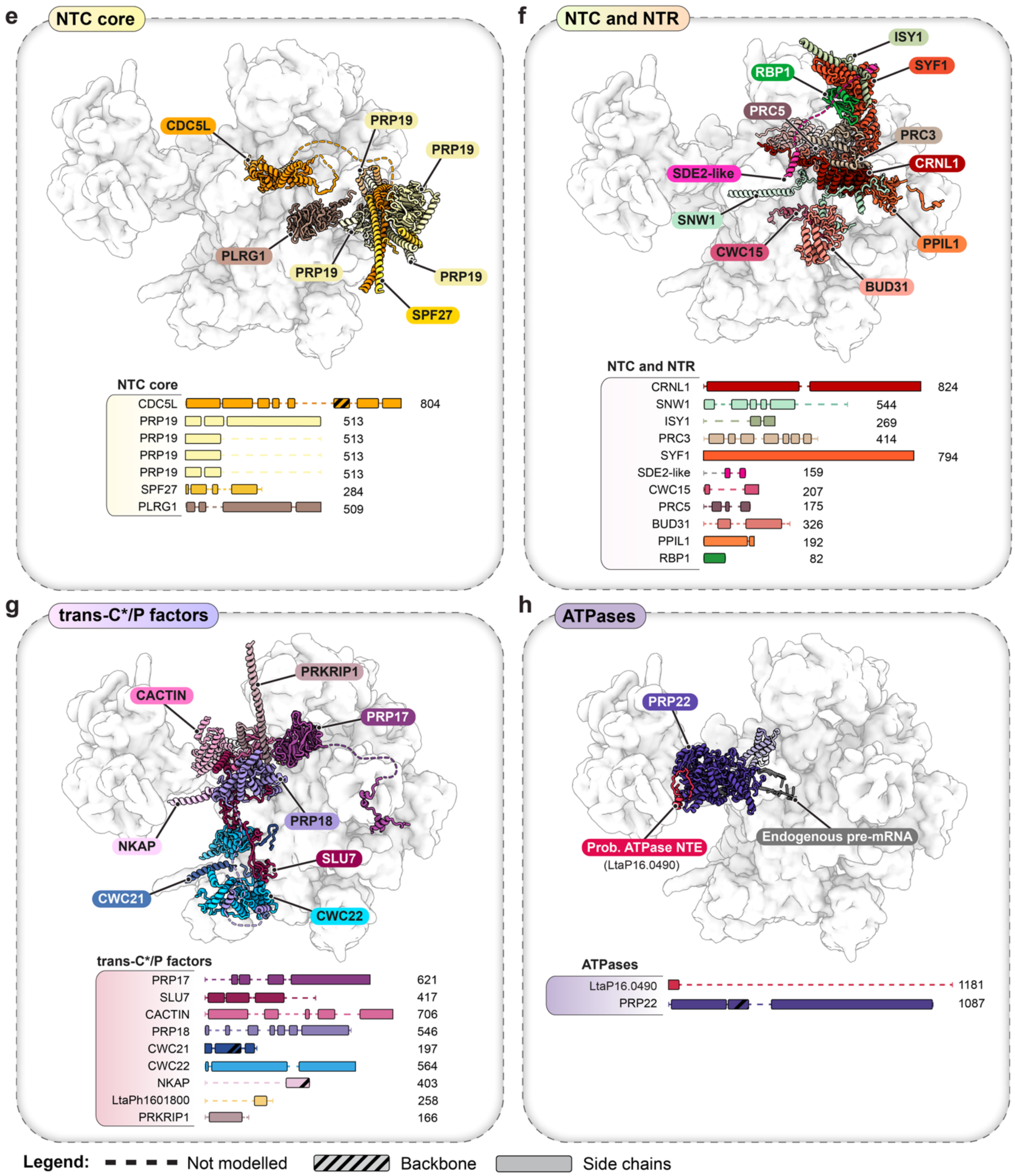

Supplementary Fig. 8. Organization of the trypanosomatid step II trans-spliceosome into distinct sub-complexes.

a. The U2 snRNP comprises U2 snRNA, the proteins U2A-40K and U2B, and a heptameric Sm ring formed by five conserved Sm proteins (SmD1, SmD2, SmE, SmF, SmG) and two trypanosomatid-specific Sm proteins (Sm15K and Sm16.5K). **b.** The U5 snRNP, forming a central

architectural scaffold, includes PRP8, BRR2, SNU114, U5-40K, and seven canonical Sm proteins (SmB, SmD1–3, SmE–G). **c.** The U6 snRNP is composed of U6 snRNA and a heptameric LSm ring (LSm2–8), which is clearly resolved and docked onto the Nineteen Complex (NTC), occupying the site typically held by the intron-stabilizing AQR helicase in human spliceosomes. **d.** The exon junction complex (EJC) includes the three conserved core components, eIF4A3, MAGOH, RBM8A, and an additional Zn-knuckle factor. The SL snRNP is composed of the SL snRNA, cleaved at the 5' splice site (5'SS), and seven canonical Sm proteins (SmB, SmD1–3, SmE–G). It protrudes outward, positioned atop BUD31, occupying the site normally held by intron-bound RBM22 in human spliceosomes or Cwc2/Ecm2 in yeast spliceosomes. **e-f.** The Nineteen Complex (NTC) core comprises CDC5L, four copies of PRP19, SPF27, and PLRG1, and is extended by NTC-related (NTR) factors including SYF1, CRNL1, BUD31, CWC15, ISY1, SNW1, and PPIL1. Trypanosomatid-specific components such as RBP1, SDE2-like, PRC3, and PRC5 further expand this module. **g.** Nine auxiliary factors are associated with the step II catalytic (trans-C*) and post-catalytic (trans-P) stages, including CACTIN, CWC21, CWC22, NKAP, PRKRIP1, PRP17, PRP18, SLU7, and the trypanosomatid-specific LtaPh1601800. **h.** The ATPase DEAH-box helicase PRP22/DHX8, whose role is to release the SL capped pre-mRNA from the trans-spliceosome, is positioned near the RT domain of PRP8 and contributes to pre-mRNA stabilization. The N-terminus of a second putative ATPase (LtaP16.0490) is bound to the RecA2 lobe of PRP22/DHX8.

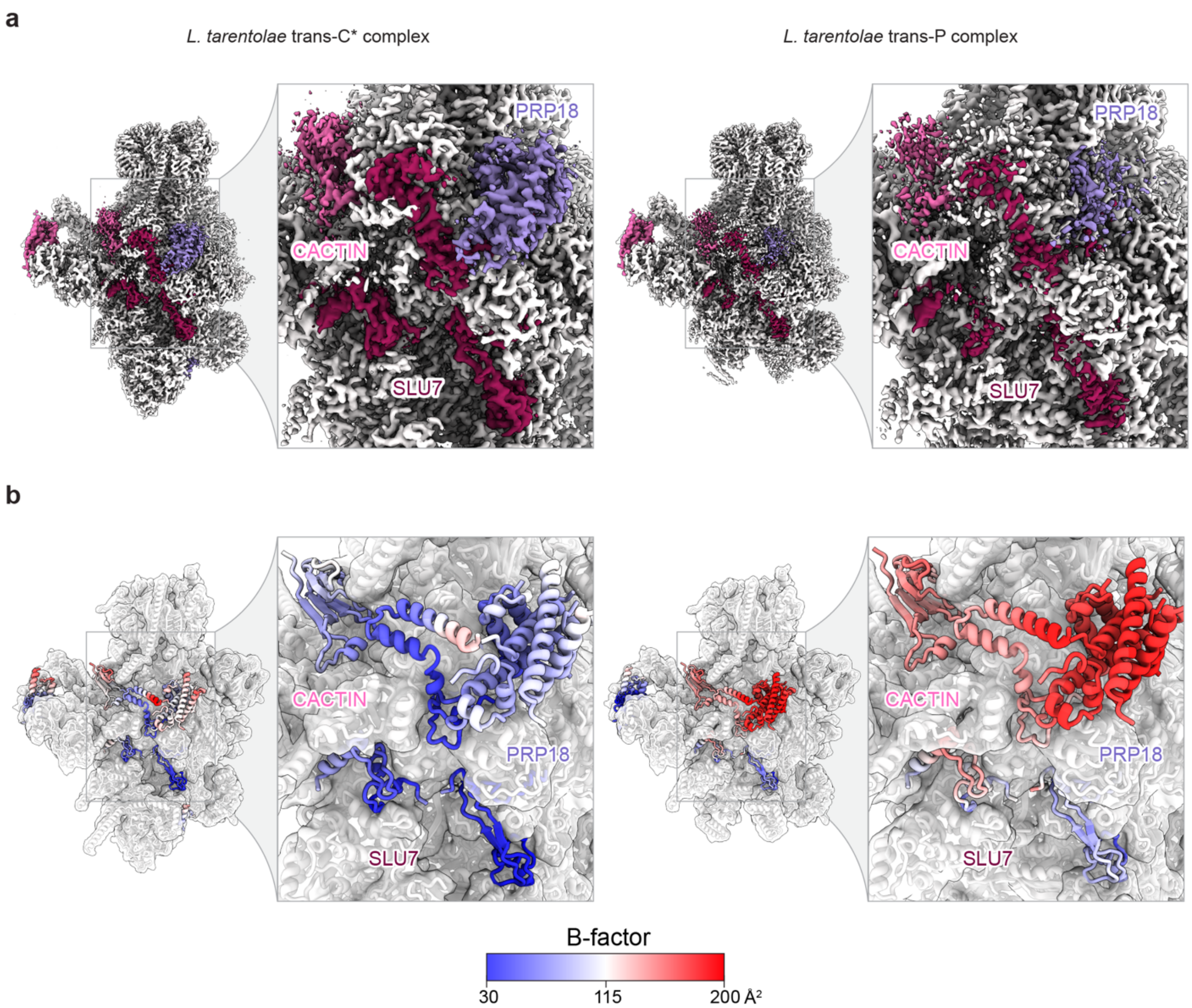

Supplementary Fig. 9. Destabilization of step II factors CACTIN, SLU7, and PRP18 in the trans-P complex.

a. Cryo-EM density of the *L. tarentolae* trans-C* (left) and trans-P (right) complexes highlighting step II factors CACTIN, SLU7, and PRP18. **b.** Corresponding B-factor mapping of CACTIN, SLU7, and PRP18, indicating increased flexibility upon transition from the trans-C* to the trans-P complex.

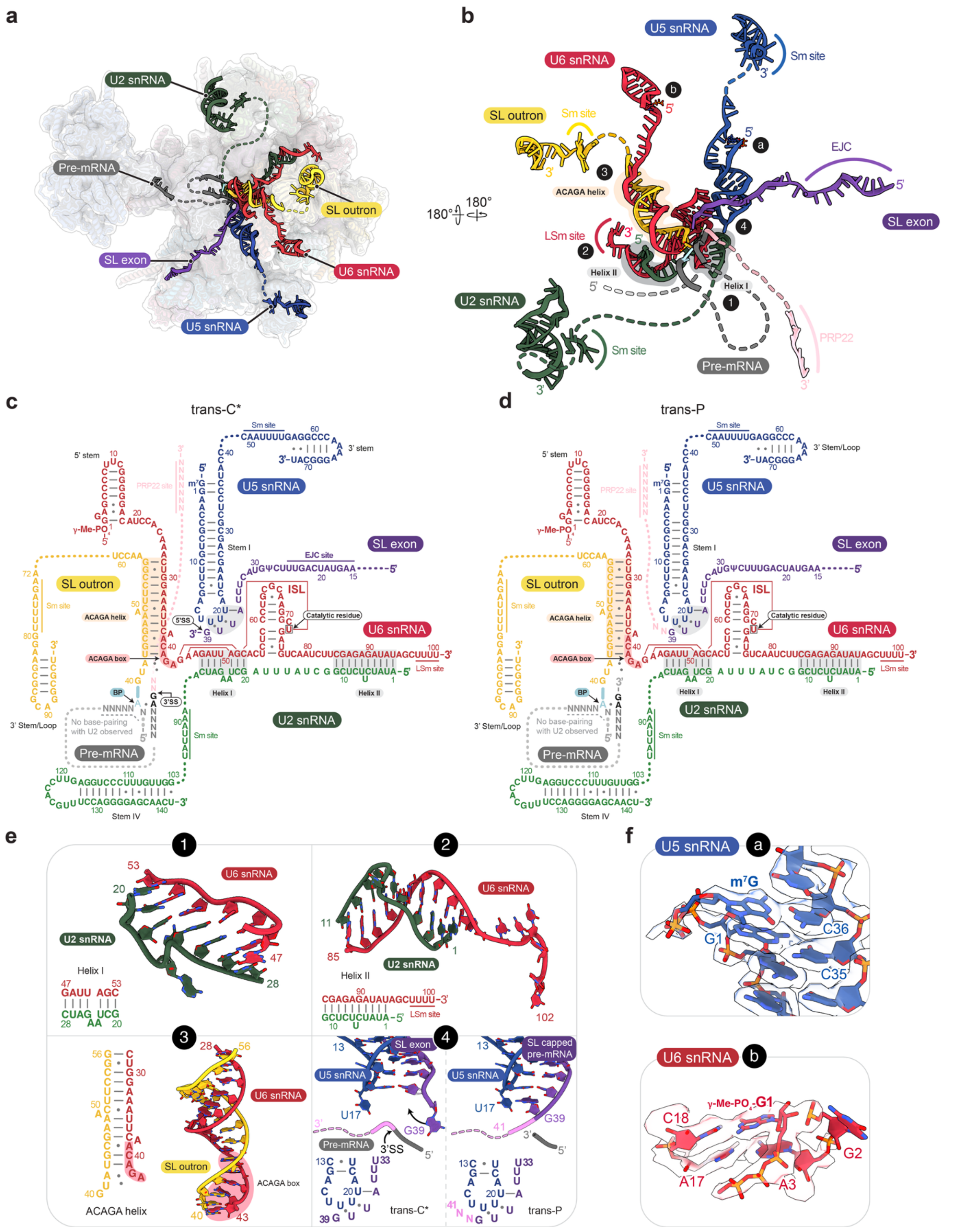

Supplementary Fig. 10. Structural network of the RNA elements in the step II trypanosomatid trans-spliceosomes.

a. Overall architecture of the SL exon, SL outron, pre-mRNA, and snRNAs within the trans-C* complex. **b.** Zoom into the trans-C* RNA interaction network, highlighting Sm, LSm,

PRP22/DHX8, and EJC binding sites as well as key helices. **c.** Base-pairing interactions among RNA elements in the trans-C* complex. **d.** Base-pairing interactions among RNA elements in the trans-P complex; for c and d, canonical Watson–Crick and non-canonical interactions are indicated by solid lines and dots. **e.** Base-pairing interactions of U2/U6 helix I, helix II, SL outron/U6 ACAGA helix, and SL exon/U5 loop I in trans-C* (left) and trans-P (right) complexes, with secondary structure schematics indicated. **f.** Chemical modifications of the 5' ends of U5 (top) and U6 (bottom) snRNAs shown in their respective cryo-EM density.

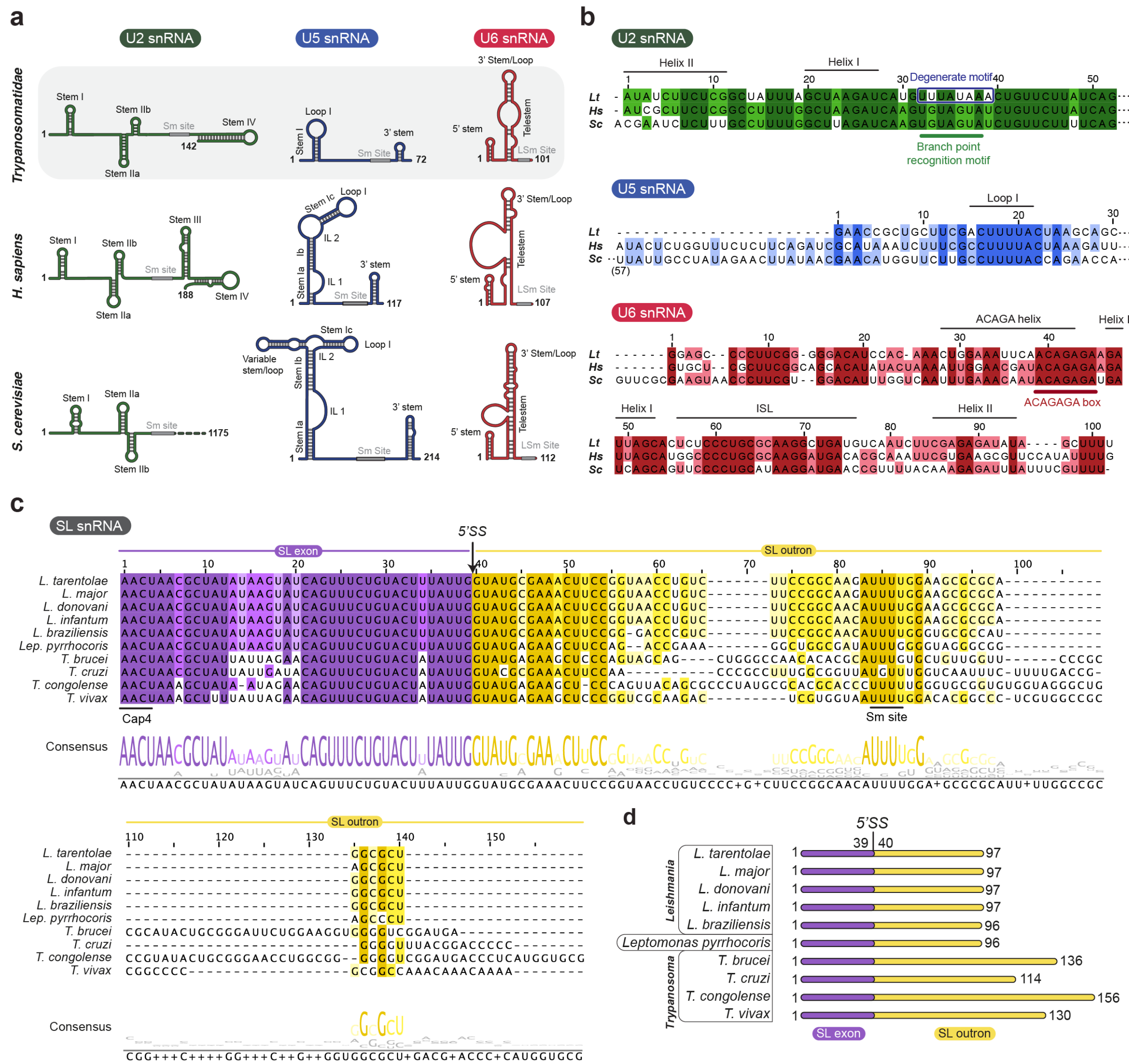

Supplementary Fig. 11. Structure and sequence of trypanosomatid SL RNA and snRNAs.

a. Comparison of trypanosomatid, human, and yeast U2, U5, and U6 snRNAs, highlighting the reduced size of trypanosomatid snRNAs. **b.** Sequence alignments of *L. tarentolae*, human, and yeast U2, U5, and U6 snRNAs, showing conserved and degenerate motifs. Notably, *L. tarentolae* U2 contains a degenerate branch point recognition motif. **c.** Sequence alignment of SL snRNAs from 10 representative trypanosomatid species, highlighting the strong conservation of both the SL exon and the SL outron segment (40–56) that base-pairs with the U6 ACAGAGA box. A slight variation in the SL exon between nucleotides 13–20 corresponds to the site where the EJC binds through nonspecific phosphate interactions. **d.** Size comparison of SL snRNAs across the same 10 trypanosomatid species, showing that *Trypanosoma* SL snRNAs are longer.

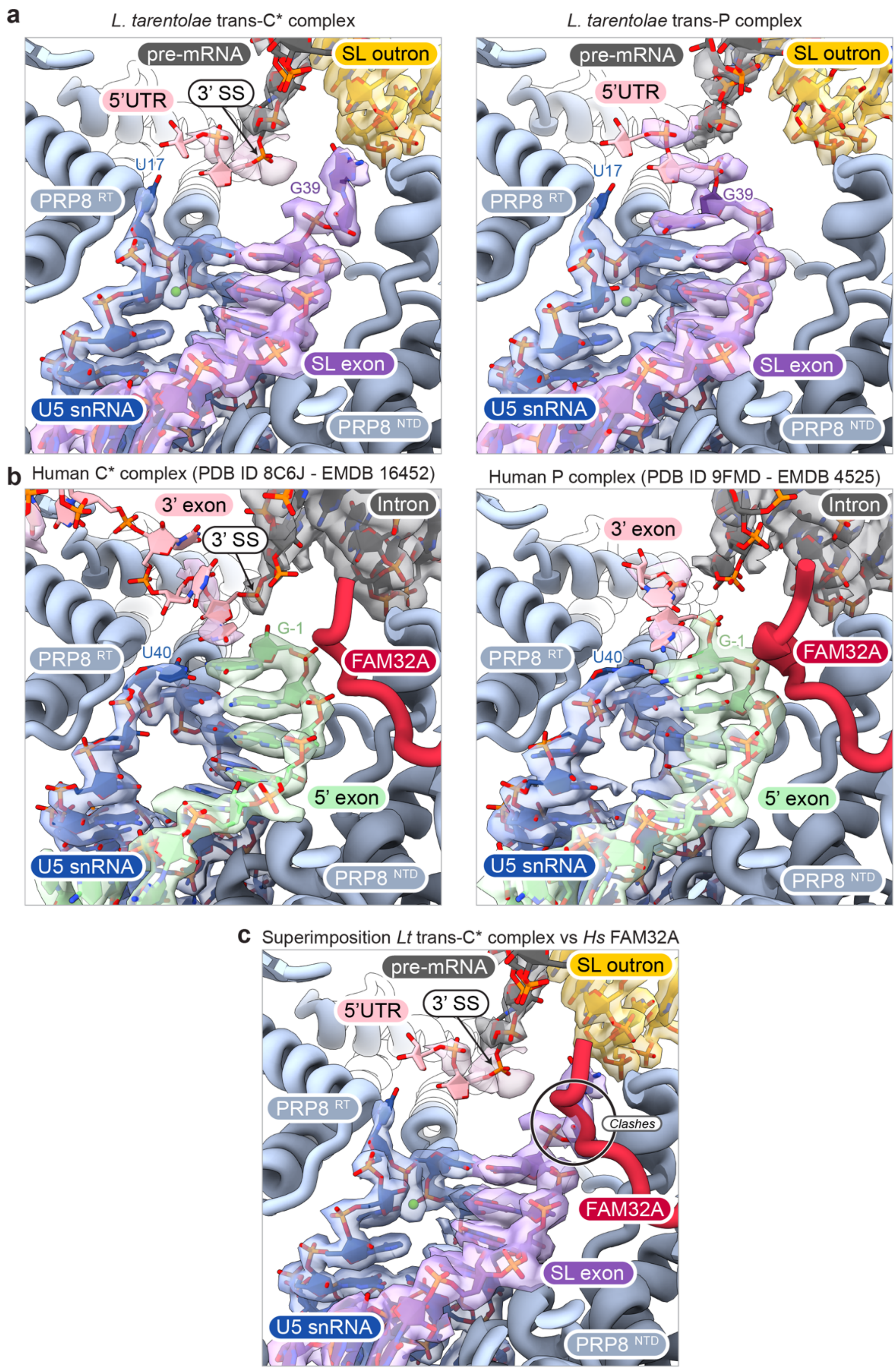

Supplementary Fig. 12. Catalytic center of trypanosomatid and human spliceosomes.

a. In the *L. tarentolae* trans-C* complex (left), before exon ligation, the last base of the SL exon (G39) is flipped out, positioned away from the 5′ UTR, and not stabilized by U17 of U5 snRNA.

G39 is likely stochastically flipping in and out until the second trans-esterification reaction occurs. This dynamic conformation is enabled by the absence of a FAM32 ortholog in trypanosomatids. In the trans-P complex, G39 is captured in the flipped-in state and ligated to the 5′ UTR of the pre-mRNA. **b.** In the human C* (left) and P (right) complexes, the 5’ exon is stabilized for ligation by FAM32A, which positions it within the catalytic core. **c.** Superimposition of the *L. tarentolae* trans-C* complex with human FAM32A, showing steric clashes between FAM32A and G39 of the SL outron. Through the panels, U2 and U6 snRNAs are omitted for clarity.

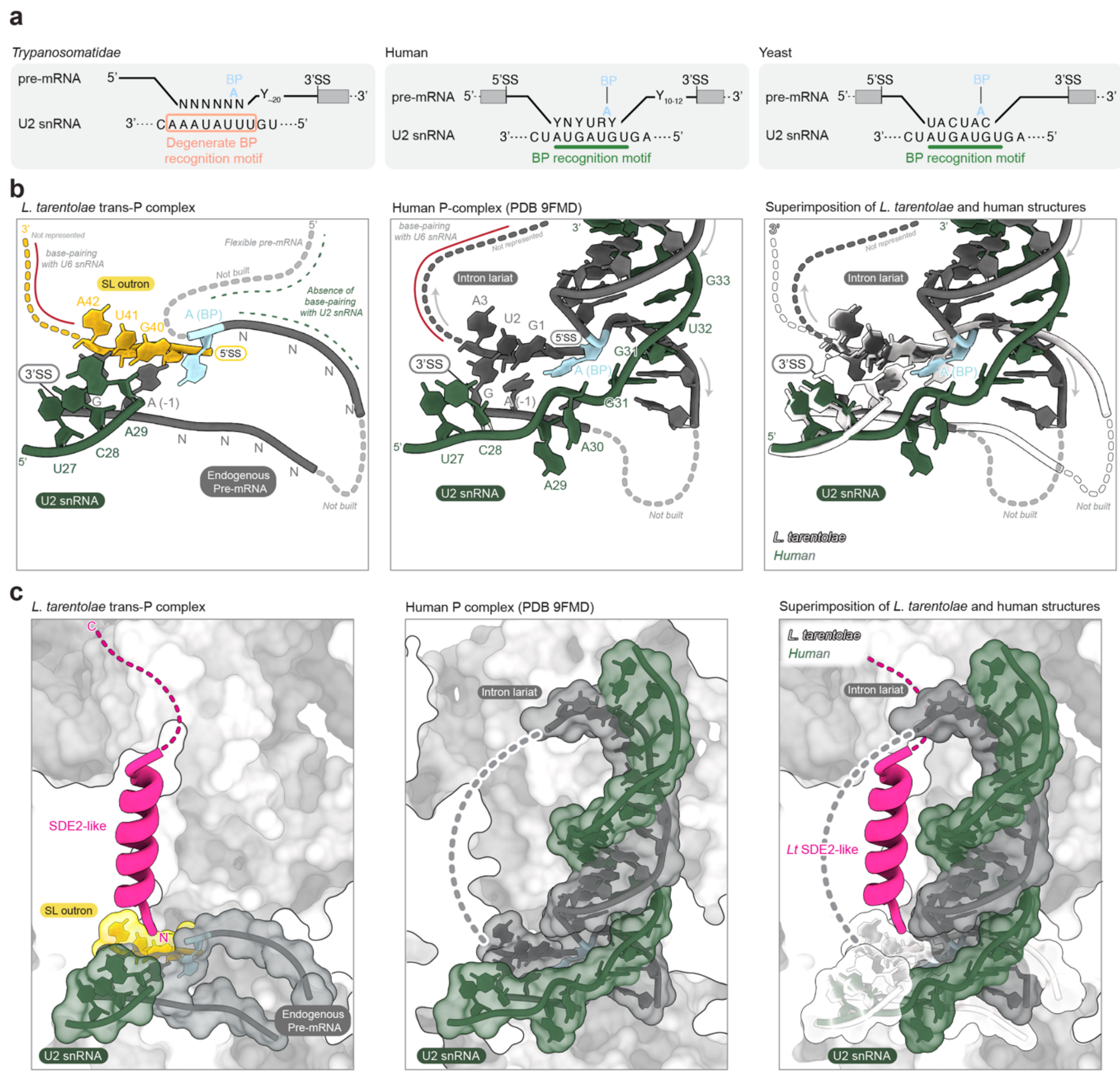

Supplementary Fig. 13. Divergent branchpoint recognition and RNA network in trypanosomatids.

a. Base-pairing interactions between U2 snRNA and pre-mRNA around the branchpoint (BP). In trypanosomatids, U2 snRNA harbors a degenerate BP recognition motif, and no consensus motif is present on the pre-mRNA for branchpoint selection. In humans, the conserved BP recognition motif on U2 snRNA pairs with a slightly permissive motif on the intron. In yeast, the conserved BP recognition motif on U2 pairs with a strongly conserved intron motif. **b.** Structural comparison of the RNA network surrounding the BP in trypanosomatids and humans. In *L. tarentolae*, the absence of conserved motifs prevents unambiguous identification of nucleotides around the BP on the endogenous pre-mRNA. No density is visible for U2 snRNA, which in humans base pairs with the intron to form the branch helix. For clarity, base-pairing of the SL outron or intron lariat with the U6 ACAGA helix is not shown. **c.** Structural comparison of the branch helix. Although the branch helix is not resolved in *L. tarentolae*, the cavity observed in the trans-P complex could accommodate it, even in the presence of the SDE2-like N-terminal helix, as demonstrated by superimposing the human branch helix.

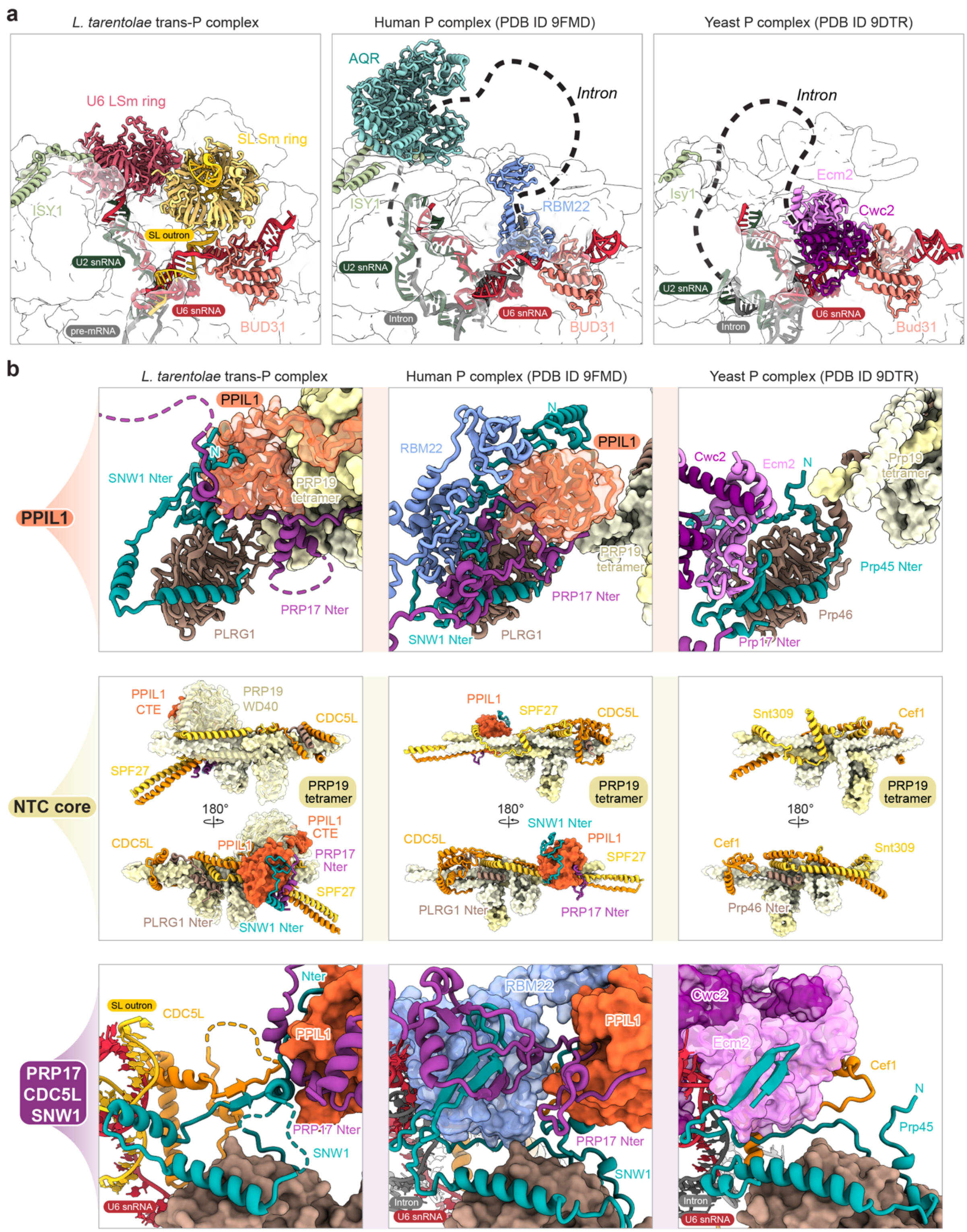

Supplementary Fig. 14. Rewiring of protein–protein interactions in the trypanosomatid trans-P complex compared with human and yeast P complexes.

a. Overview of the *L. tarentolae* trans-P complex compared with human (PDB 9FMD) and yeast (PDB 9DTR) P complexes. Proteins typically associated with intron stabilization, such as RBM22

(Cwc2/Ecm2 in yeast) and the AQR helicase, are absent in trypanosomatids, reflecting structural adaptations to SL trans-splicing. RBM22 is replaced by the Sm ring of the SL snRNP, while the U6 LSm ring occupies the position normally held by the AQR helicase on SYF1. **b.** Detailed architecture of the NTC–NTR across species. Top row: Interactions of PPIL1 with the N-terminal tails of PRP17 and SNW1 vary markedly across species. In trypanosomatids, the absence of RBM22/Cwc2/Ecm2 generates a distinct and simplified interface with PPIL1, which has evolved an extended C-terminal tail that engages the WD40 domain of PRP19, positioned atop the PRP19 tetramer. In humans, the presence of RBM22 remodels the interface among the three proteins, promoting an extended interaction network. In yeast, the PRP17 N-terminal tail is not stabilized, likely due to the absence of PPIL1. Middle row: isolated views of the NTC core, highlighting conserved and divergent contacts. Bottom row: species-specific differences at the interface between SNW1, CDC5L, and PRP17 in trypanosomatids compared to human and yeast, where RBM22 and Cwc2/Ecm2 are present. In humans, an extensive interaction network between PRP17 and SNW1 N-terminal tails and RMB22 is observed.

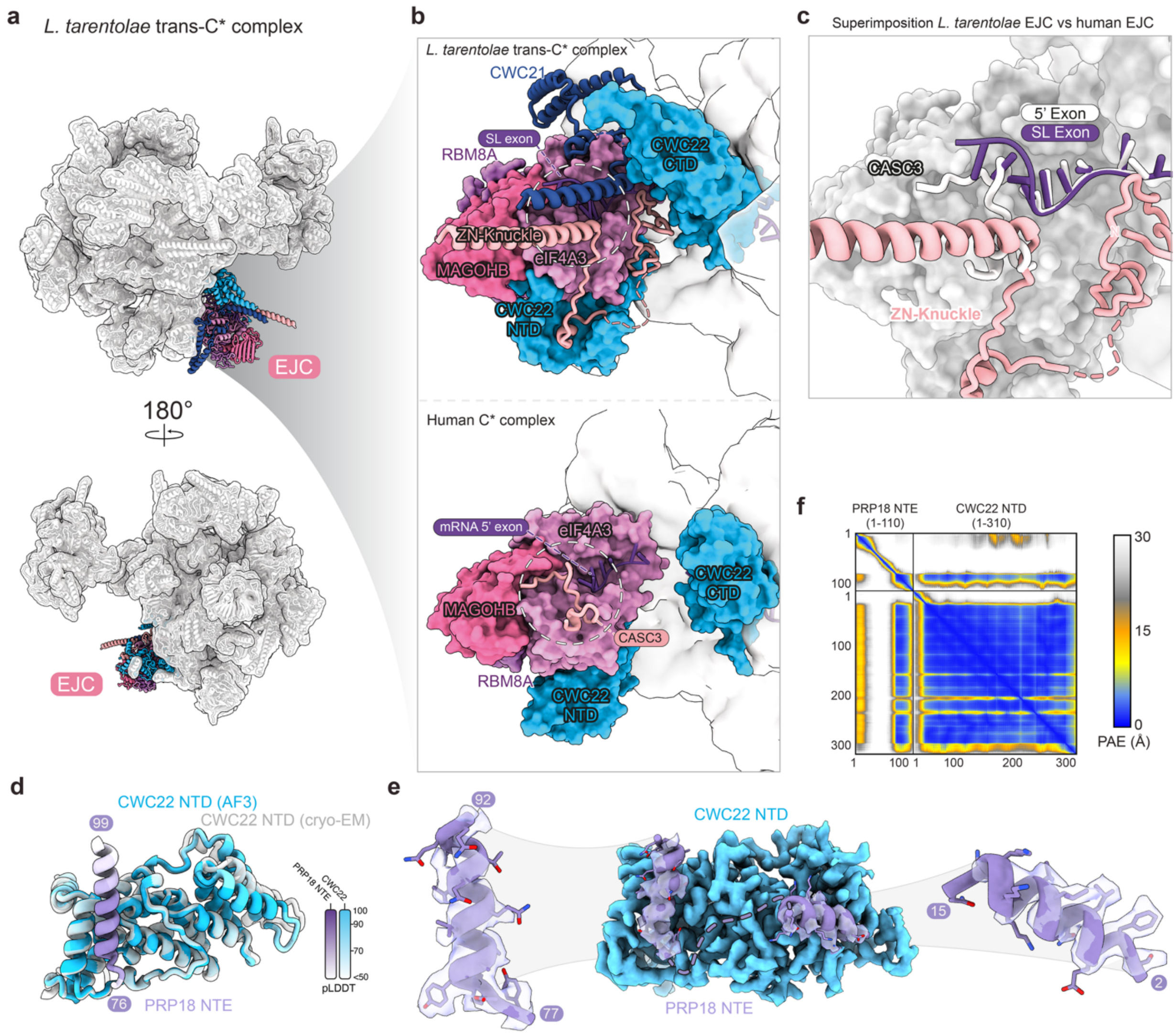

Supplementary Fig. 15. Comparison of the trypanosomatid and human EJC.

a. EJC in the context of the *L. tarentolae* trans-C* complex. **b.** Top: *L. tarentolae* EJC showing core proteins eIF4A3, MAGOH, and RBM8A, stabilized by CWC22, in surface representation, with the additional Zn-knuckle factor and CWC21 CTE depicted in cartoon representation. Bottom: human EJC showing core proteins eIF4A3, MAGOH, and RBM8A, stabilized by CWC22, in surface representation, with CASC3 depicted in cartoon representation (PDB 7W5A). **c.** Superimposition of *L. tarentolae* and human EJCs reveals an overlapping binding site for CASC3 and the C-terminal helix of the Zn-knuckle. The Zn-knuckle in *L. tarentolae* may play a role analogous to CASC3 in mRNA regulation. **d.** AlphaFold3 prediction of the interaction between the PRP18 N-terminal extension (NTE) and the CWC22 N-terminal domain (NTD). Confidence levels (pLDDT) are color-coded for each protein. Notably, AF3 does not predict an interaction between the distal PRP18 NTE (residues 1–15) and the CWC22 NTD. This may be explained by the closed conformation of the CWC22 NTD in the prediction, in contrast to the more open conformation observed in the cryo-EM structure, which can accommodate the distal PRP18 NTE. **e.** Cryo-EM density of the PRP18 NTE in complex with CWC22, with insets highlighting the well-resolved hydrophobic residues at the protein-protein interface. **f.** AlphaFold3 PAE plot.

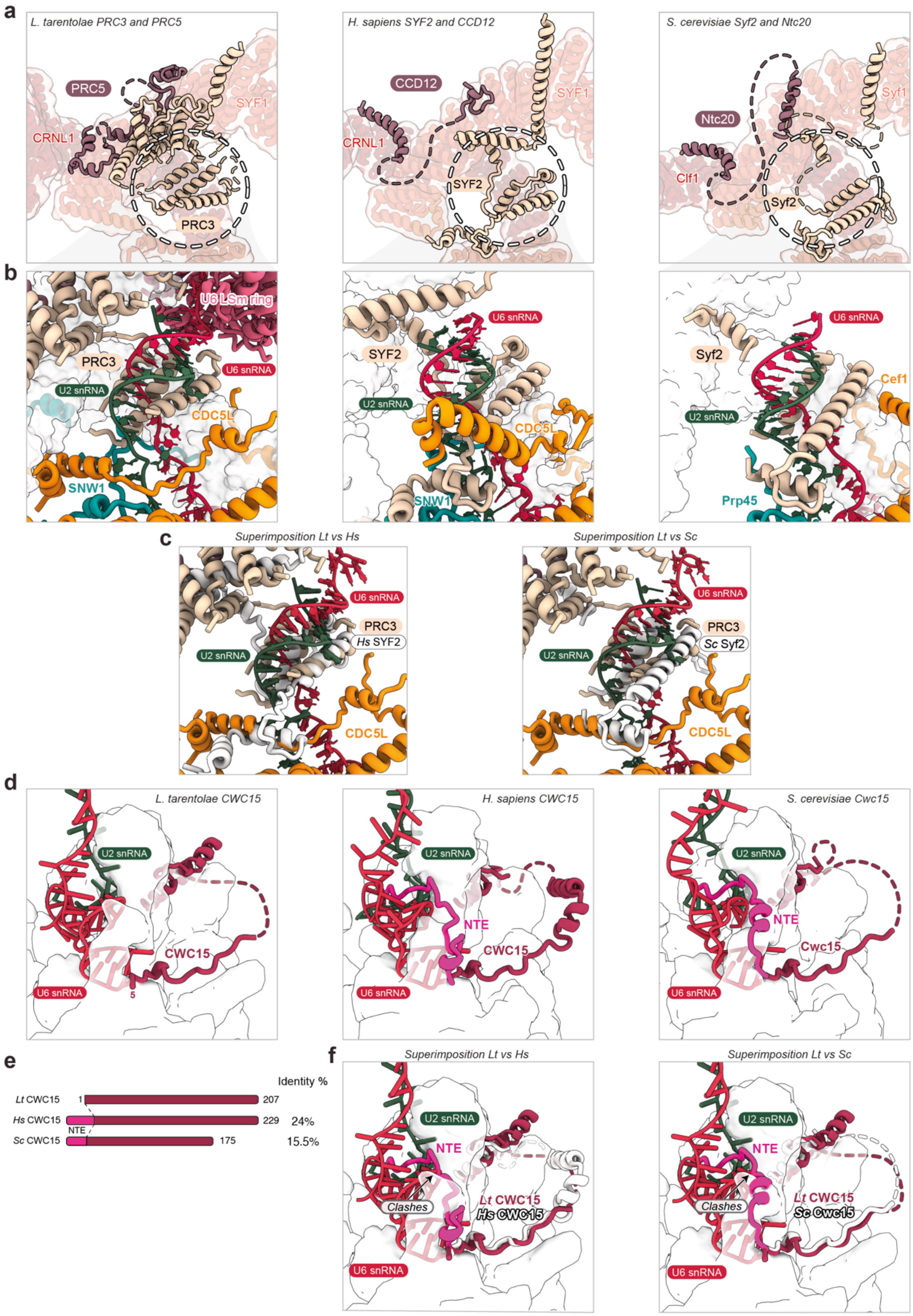

Supplementary Fig. 16. Structural adaptations of PRC3 and PRC5 stabilizing U2/U6 helix II.

a. Comparison of the binding modes of PRC3 and PRC5 in trypanosomatids, SYF2 and CCDC12 in humans (PDB 9FMD), and Syf2 and Ntc20 in yeast (PDB 9DTR). PRC3 and PRC5 form an

extensive interaction network with CRNL1 and SYF1 compared to their functional analogs. **b.** Close-up view of U2/U6 helix II stabilized by the PRC3 C-terminal triple helices. Comparison with human and yeast structures highlights a bent conformation of helix II, likely induced by the presence of the LSm ring. CDC5L/Cef1 also adopts distinct paths depending on the species. **c.** Superimposition of human and yeast SYF2/Syf2 onto the *L. tarentolae* structure shows that the absence of a CTD tail in PRC5 is compensated by CDC5L, which stabilizes the base of helix II. **d.** Close-up view of the helix II base in *L. tarentolae*, human, and yeast reveals that in humans and yeast, CWC15 stabilizes the helix base by inserting its N-terminal extension (NTE) into an unpaired bubble between U2 and U6 atop helix Ib. The trypanosomatid CWC15 lacks this NTE which is compensated by distinct U2/U6 helices I and II architectures. **e.** Domain organization of *L. tarentolae* (*Lt*) CWC15 compared with human (*Hs*) and yeast (*Sc*) orthologs, with percentage sequence identities indicated. **f.** Superimposition of human and yeast CWC15 onto the *L. tarentolae* structure highlights steric clashes of the CWC15 NTE with U6 snRNA, likely caused by a slightly more open U2/U6 bubble in trypanosomatids.

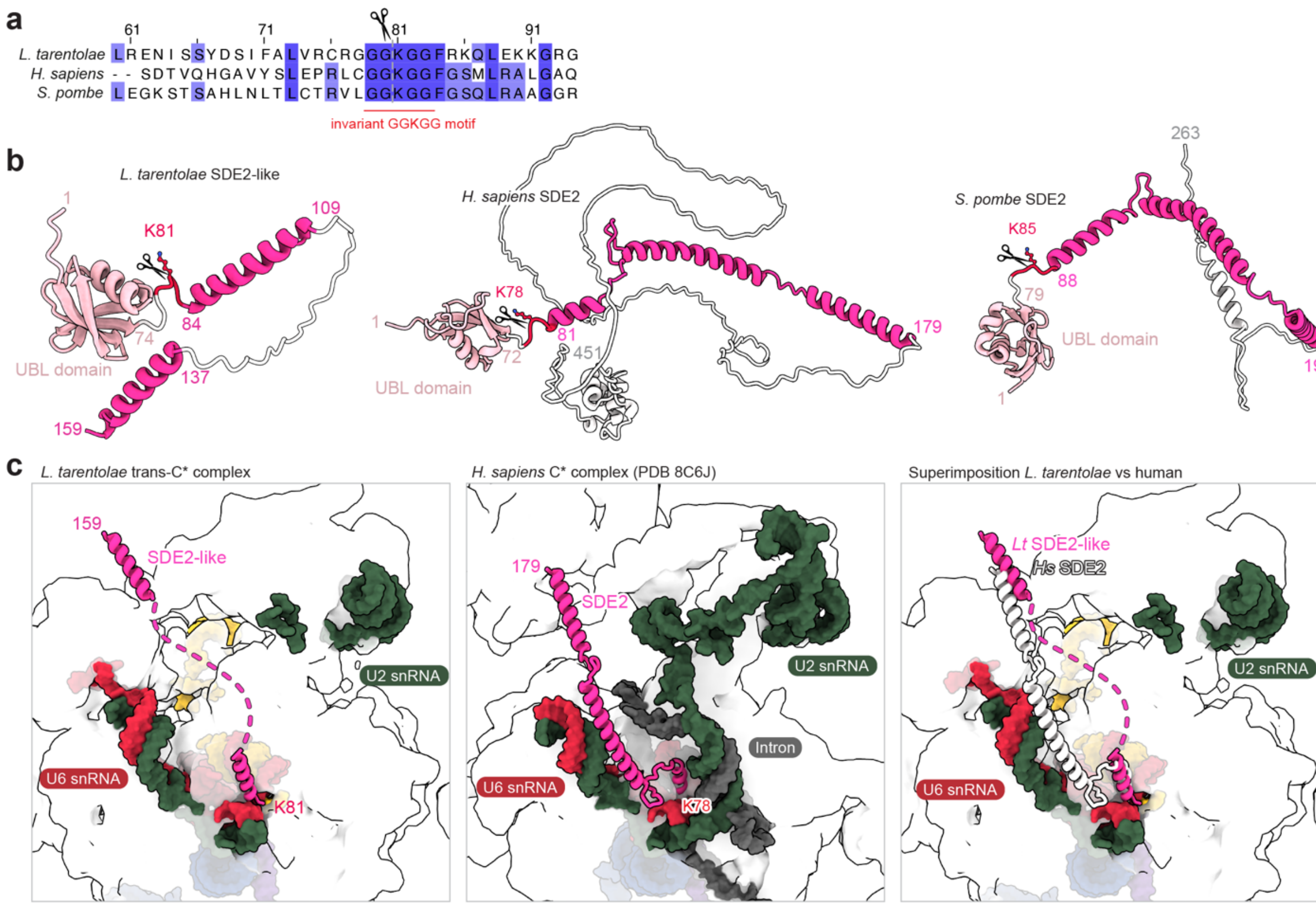

Supplementary Fig. 17. Structural analogy of *Leishmania tarentolae* SDE2-like with SDE2 in opisthokonts.

a. Sequence alignment of SDE2 orthologs highlighting the conserved proteolytic motif required for maturation. The invariant glycine residues motif (GGKGG) and cleavage site are indicated. **b.** Domain organization and structural representation of SDE2 proteins from different species (AlphaFold ID: Human, AF-Q6IQ49-F1*;* *S. pombe*, AF-O14113-F1). The UBL domain (light pink) is removed by proteolytic cleavage, generating the activated SDE2-C fragment (magenta). Residue numbering and key structural elements are indicated. The *L. tarentolae* SD2-like protein structure was predicted using AlphaFold3 from the protein sequence provided in Supplementary Table 4. **c.** Localization of the activated SDE2 fragment (magenta) within the (trans-)spliceosome across species. In the *L. tarentolae* trans-spliceosome, the N-terminus of SDE2 is positioned near U2/U6 helix I, while its C-terminal helix docks onto SYF1 above U2/U6 helix II. Comparison with the human C* complex reveals a conserved spatial arrangement. Superposition highlights the structural analogy despite evolutionary divergence.

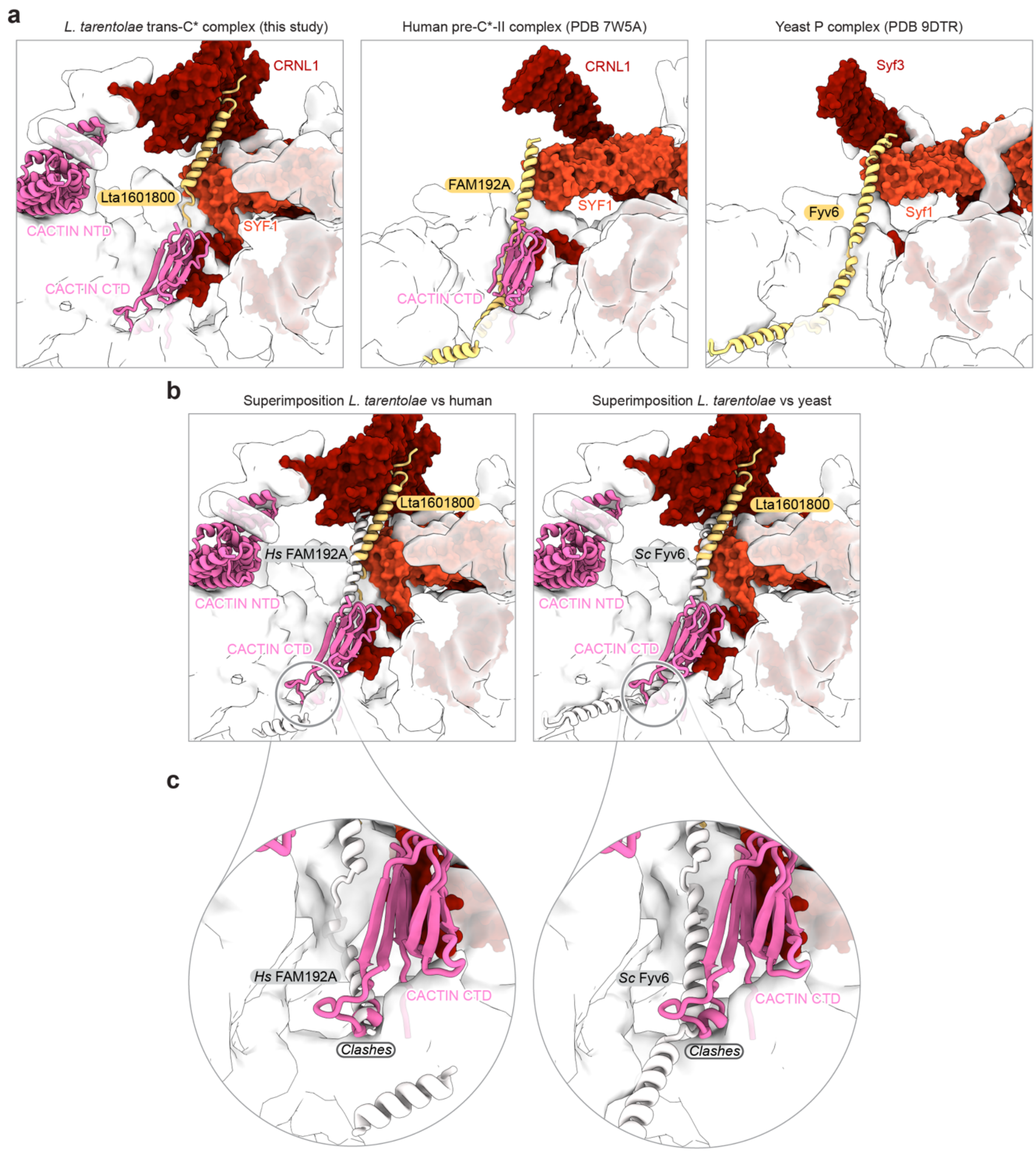

Supplementary Fig. 18. Functional analogy of LtaPh1601800 with FAM192A and Fyv6.

a. Structural comparison of the LtaPh1601800 binding site in *L. tarentolae* with its counterparts in humans (PDB 7W5A) and yeast (PDB 9DTR). In *L. tarentolae*, LtaPh1601800 associates with SYF1 and the extended CRNL1 CTD at the position typically occupied by FAM192A and Fyv6 in C* complexes. Since both FAM192A and Fyv6 are implicated in 3′ splice site selection, this suggests a similar role for LtaPh1601800. **b.** Superimposition of human FAM192A (left) and yeast Fyv6 (right) onto the *L. tarentolae* structure highlights the overlapping binding site on SYF1/Syf1. **c.** Close-up view of *L. tarentolae* CACTIN CTD with superimposed human FAM192A and yeast Fyv6 reveals steric clashes between these proteins and the CACTIN CTD, which may explain why the remainder of LtaPh1601800 is not resolved in the structure.

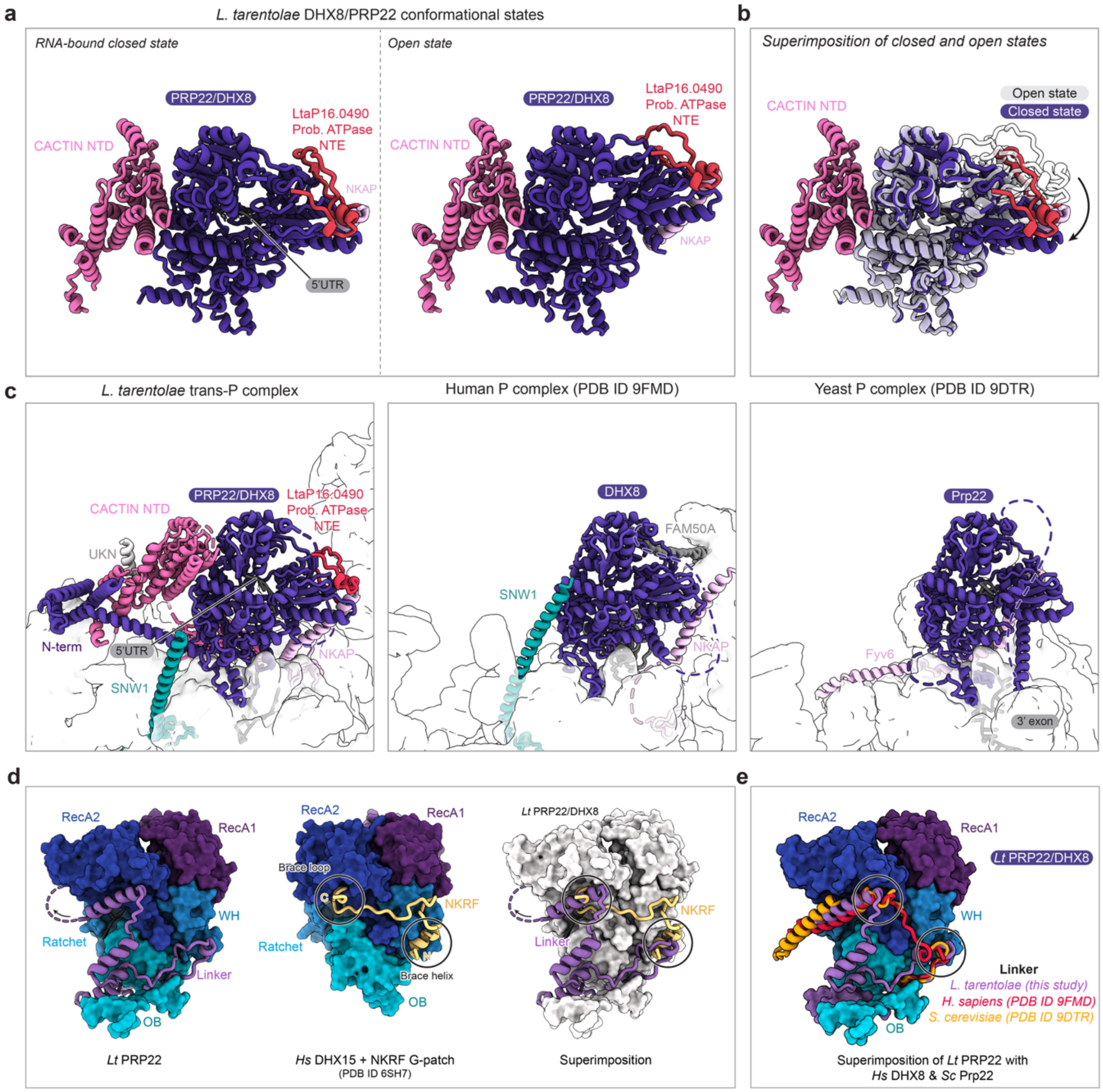

Supplementary Fig. 19. Conformational equilibrium and protein network of *L. tarentolae* PRP22/DHX8.

a. Structures of *L. tarentolae* PRP22/DHX8 in RNA-bound closed (left) and open (right) states. **b.** Superimposition of the RNA-bound closed (colored) and open (gray) states highlights conformational changes of the RecA2 lobe and the associated helicase NTE. **c.** Comparison of *L. tarentolae* PRP22 within the trans-P complex with human DHX8 (PDB 9FMD) and yeast Prp22 (PDB 9DTR) within the P complex, showing the distinct protein network in trypanosomatids that locks PRP22 in a pre-activated state, preventing translocation activity and avoiding premature pre-mRNA release. **d.** Comparison of *L. tarentolae* PRP22 with human DHX15 bound to the NKRF G-patch (PDB 6SH7) [55]. Superposition shows that the PRP22 linker occupies a position analogous to the NKRF G-patch, suggesting a conserved regulatory mechanism that likely stabilizes the helicase in an activated RNA-binding conformation. **e.** Superimposition of the PRP22/DHX8 linkers from *L. tarentolae*, human and yeast showing conserved binding sites with NKRF G-patch.

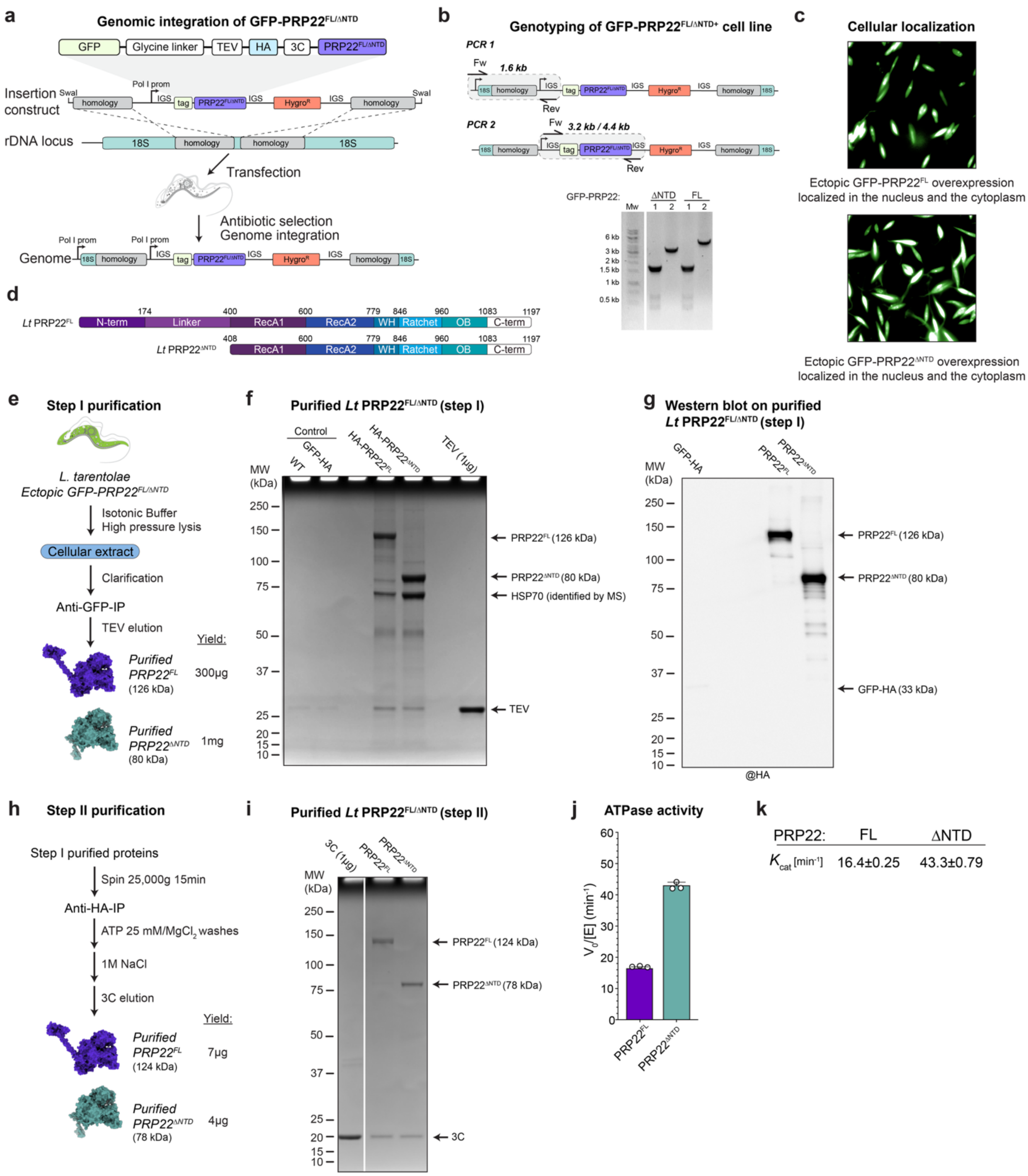

Supplementary Fig. 20. Expression, purification, and ATPase characterization of PRP22 variants in *Leishmania tarentolae.*

a. Schematic of the strategy used to integrate an ectopic copy of GFP-tagged PRP22 (full-length or ΔNTD) into one rDNA locus in *L. tarentolae*. The construct includes an N-terminal GFP tag followed by a TEV-cleavable glycine linker and a 3C-cleavable HA epitope. **b.** Genotyping of GFP–PRP22 cell lines confirming correct genomic integration. Two independent PCR reactions validate insertion of the construct at the rDNA locus for both full-length (FL) and ΔNTD variants.

Representative agarose gel analysis is shown. **c.** Fluorescence microscopy images of cells expressing GFP–PRP22^{FL} and GFP–PRP22$^{\Delta NTD}$, showing localization in both the nucleus and cytoplasm. **d.** Domain organization of *L. tarentolae* PRP22 highlighting the N-terminal region, linker, and conserved helicase domains (RecA1, RecA2, WH, ratchet, OB, and C-terminal domains), and the truncation used for the ΔNTD construct. **e.** Workflow for the GFP-based purification (step I) of PRP22 variants from *L. tarentolae* cells. **f.** SDS–PAGE analysis of purified PRP22^{FL} and PRP22$^{\Delta NTD}$ proteins obtained from step I, stained with Coomassie. The positions of HA-PRP22^{FL} (~126 kDa), HA-PRP22$^{\Delta NTD}$ (~80 kDa), HSP70 (~70 kDa) and TEV protease are indicated. **g.** Western blot analysis of step I-purified PRP22^{FL} and PRP22$^{\Delta NTD}$ using anti-HA antibody. The analysis was only performed once. **h.** Workflow for the HA-based purification (step II) of PRP22 variants. **i.** SDS–PAGE analysis of purified PRP22^{FL} and PRP22$^{\Delta NTD}$ proteins obtained from step II, stained with Coomassie. The positions of PRP22^{FL} (~124 kDa), PRP22$^{\Delta NTD}$ (~78 kDa) and 3C protease are indicated. **j.** Comparison of ATP turnover rates (v_0/[E]) for PRP22^{FL} and PRP22$^{\Delta NTD}$, measured as initial ATP hydrolysis rates at 2 mM ATP and normalized for enzyme concentrations, using a continuous coupled assay. Error bars represent SD from triplicate measurements (n=3). Individual data points are indicated. **k.** Summary of K_{cat} values derived from ATPase assays for PRP22 variants. Source data and uncropped gels and blots are provided in the Source Data file.

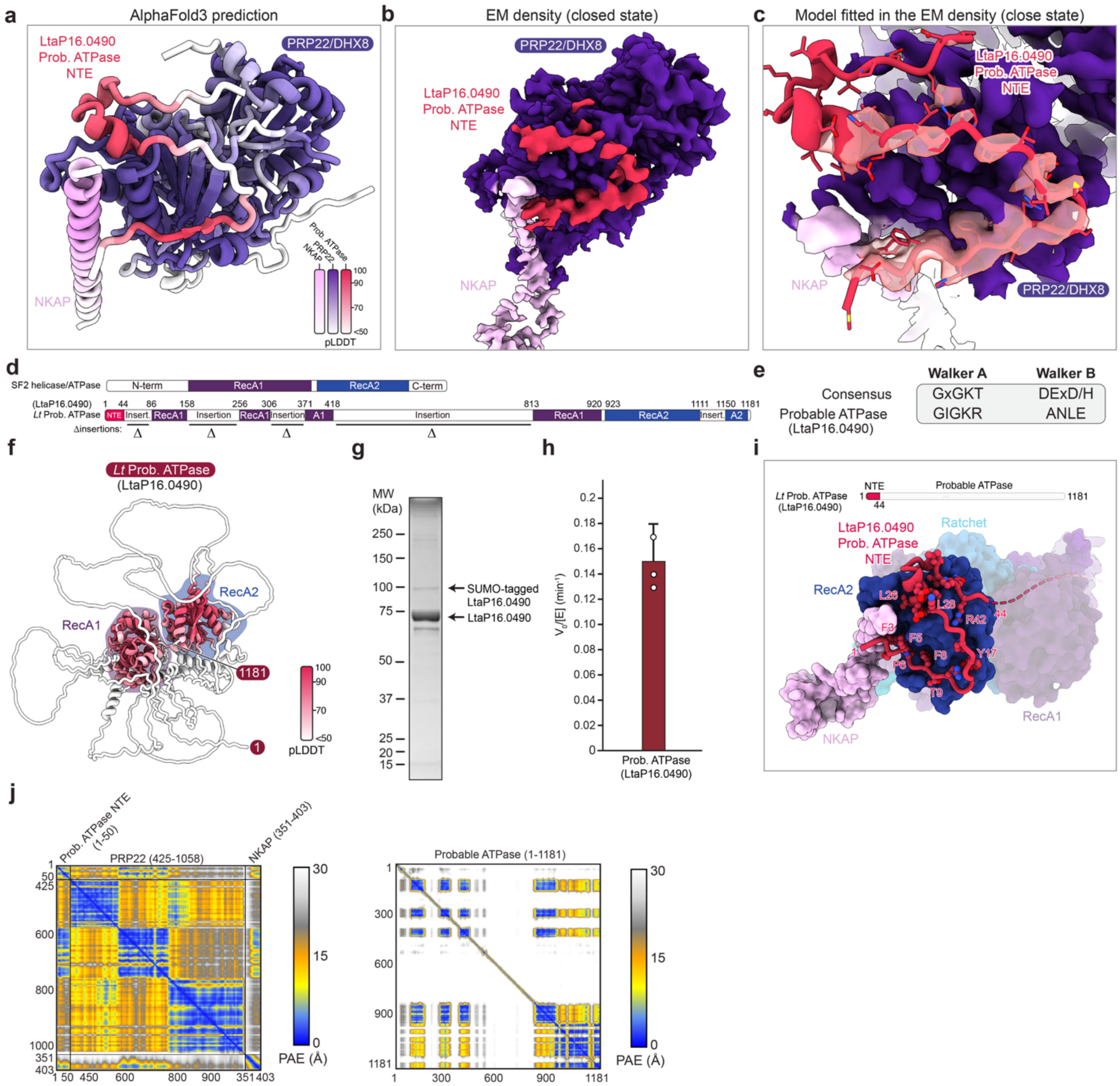

Supplementary Fig. 21. Structural and functional characterization of a PRP22-associated non-canonical ATPase (LtaP16.0490).

a. AlphaFold3 prediction of the interaction between PRP22/DHX8 and the N-terminal region of LtaP16.0490 (hereafter referred to probable ATPase). Confidence levels (pLDDT) are color-coded for each protein. **b.** Cryo-EM density corresponding to the probable ATPase bound to PRP22/DHX8 in a closed state. **c.** AlphaFold3 prediction fitted and adjusted into the cryo-EM density shown in panel b, highlighting the good fit of aromatic and aliphatic residues at the interface between the two proteins. **d.** Domain architectures of a generic SF2 helicase/ATPase and the probable ATPase, highlighting multiple loop insertions in the RecA1 domain of the probable ATPase. **e.** Comparison of Walker A and Walker B motifs between canonical DExD/H helicases and the probable ATPase, highlighting degeneration of the Walker B motif in the probable ATPase. **f.** AlphaFold3 model of the probable ATPase colored by pLDDT confidence. The two RecA domains are indicated. **g.** SDS–PAGE analysis of the purified probable ATPase lacking

insertion regions (Δinsertions), as indicated in d, stained with Coomassie. The position of the truncated protein (~74 kDa) is indicated. Uncropped gel in Source Data. **h.** ATPase activity of the probable ATPase, measured as initial ATP hydrolysis rates at 2 mM ATP, normalized for enzyme concentrations. The error bar represents standard deviation (SD) from triplicate measurements (n=3). Individual measurements are indicated. **i.** Structural details of the PRP22 RecA2 lobe decorated by the NTE (residues 1-44, red) of LtaP16.0490, a non-canonical ATPase, involving many aromatic and apolar residues. **j.** AlphaFold3 PAE plots for structure predictions in panels (**a**) and (**f**). Source data are provided as a Source Data file.

Supplementary Table 1. Overall, focused and composite cryo-EM maps generated in this study.

Overall & composite maps					
	**EMDB ID**	**Map**	**Particles no°**	**B-factor sharpening**	**Resolution (FSC-0.143)**
**trans-C***	EMD-52793	Overall	184554	-64.36	2.69
	EMD-52843	Composite			
**trans-P**	EMD-52799	Overall	141035	-65.65	2.83
	EMD-52844	Composite			

Focused maps					
	**EMDB ID**	**Map**	**Particles no°**	**B-factor sharpening**	**Resolution (FSC-0.143)**
**trans-C***	EMD-52794	Core	184554	-61.48	2.65
	EMD-52795	EJC	184554	-64.25	2.85
	EMD-52796	SNU114	184554	-87.10	3.33
	EMD-52797	U5-40K	184554	-68.00	3.10
	EMD-52798	U6 LSm ring	184554	-65.80	2.99
**trans-P**	EMD-52800	Core	141035	-60.83	2.84
	EMD-52801	CWC22	141035	-66.30	2.99
	EMD-52802	SNU114	141035	-66.32	3.04
**Merged trans-C* & trans-P**	EMD-52803	NTC-NTR 1	325589	-82.39	3.36
	EMD-52804	NTC-NTR 2	325589	-116.33	3.66
	EMD-52805	U2 snRNP	325589	-68.53	2.90
	EMD-52806	U5 Sm ring	325589	-81.65	3.04
	EMD-52807	NTC core	325589	-74.14	3.00
	EMD-52808	PRP22 closed	98912	-85.82	3.51
	EMD-52809	PRP22 open	115573	-92.76	3.69

Supplementary Table 2. Splicing proteins identified in *L. tarentolae* trans-C* and trans-P complexes and their orthologs/functional analogs in yeast and human.

Color coding corresponds to the functional classification used throughout the manuscript and figures. The proteins indicated in bold are unique to trypanosomatids. Italicized proteins are functional analogs. An asterisk (*) indicates proteins identified in TriTrypDB but not referenced in Uniprot. A double asterisk (**) marks genes absent from both UniProt and TriTrypDB but identified by BLASTN searches using orthologs from *Trypanosoma* species. The corresponding protein sequences are available in Supplementary Table 4.

	This study		orthologs/analogs		
	**Uniprot ID**	***L. tarentolae***	***S. cerevisiae***	***H. sapiens***	**Color**
**U2 snRNP**	A0A640KNR9	U2A-40K	Lea1	RU2A	
	A0A640KKX2	U2B	Msl1	RU2B	
	A0A640KNU1	**Sm15K**	unknown	unknown	
	A0A640KZZ6	**Sm16.5K**	unknown	unknown	
**U5 snRNP**	A0A640KTJ2	PRP8	Prp8	PRPF8	
	A0A640KDJ4	BRR2	Brr2	BRR2	
	A0A640KRB5	SNU114	Snu114	SNU114	
	A0A640KKK2	U5-40K	unknown	SNR40	
**SL/U2/U5 Sm proteins**	A0A640KQU2	SmB	SmB1	RSMB	
	A0A640KHR4	SmD1	SmD1	SMD1	
	A0A640KR16	SmD2	SmD2	SMD2	
	A0A640KTK0	SmD3	SmD3	SMD3	
	A0A640KLY5	SmE	SmE1	RUXE	
	A0A640KUD7	SmF	Smx3	RUXF	
	A0A640KQ84	SmG	Smx2	RUXG	
**U6 LSm proteins**	A0A640KDV9	LSm2	LSm2	LSM2	
	A0A640K801	LSm3	LSm3	LSM3	
	A0A640KQL9	LSm4	LSm4	LSM4	
	A0A640KR67	LSm5	LSm5	LSM5	
	A0A640K754	LSm6	LSm6	LSM6	
	A0A640KI01	LSm7	LSm7	LSM7	
	A0A640KIZ1	LSm8	LSm8	LSM8	
**EJC**	A0A640KRB9	eIF4A3	eIF4A/Fal1	eIF4A3	
	A0A640KMM3	MAGOHB	Mnh1	MAGOHB	
	A0A640KP41	RBM8A	unknown	RBM8A/Y14	
	A0A640KSY0	**Zn-Knuckle**	unknown	unknown	
**NTC core**	A0A640KDH5	CDC5L	Cef1	CDC5L	
	A0A640KWH7	PLRG1	Prp46	PLRG1	
	A0A640KLK2	PRP19	Prp19	PRPF19	
	A0A640KPK5	SPF27	Snt309	SPF27	

	This study		orthologs/analogs		
	**Uniprot ID**	***L. tarentolae***	***S. cerevisiae***	***H. sapiens***	**Color**
**NTC-NTR**	A0A640KB76	BUD31	Bud31	BUD31	
	A0A640KV53	CRNL1	Clf1	SYF3	
	A0A640KWX5	CWC15	cwc15	CWC15	
	A0A640KE26	ISY1	Isy1	ISY1	
	A0A640KHX2	PPIL1	unknown	PPIL1	
	A0A640KRN7	**PRC3**	*Syf2*	*SYF2*	
	A0A640KWM9	**PRC5**	*Ntc20*	*CCD12*	
	**	**RBP1**	unknown	unknown	
	**	**SDE2-like**	unknown	unknown	
	LtaP15.1050*	SNW1	Prp45	SNW1	
	A0A640KGK2	SYF1	Syf1	SYF1	
**trans-C* and trans-P factors**	A0A640KRR3	CACTIN	unknown	CACTIN	
	A0A640K740	CWC21	Cwc21	*SRRM2*	
	A0A640KX85	CWC22	Cwc22	CWC22	
	A0A640KDU3	**Lta_1601800**	*Fyv6*	*FAM192A*	
	A0A640KXH6	NKAP	unknown	NKAP	
	A0A640KD47	PRKRIP1	unknown	PRKRIP1	
	A0A640KJ83	PRP17	cdc40	CDC40	
	A0A640KFR7	PRP18	Prp18	PRPF18	
	A0A640KB40	SLU7	Slu7	SLU7	
**ATPases**	A0A640KVV3	PRP22	Prp22	DHX8	
	LtaP16.0490*	**Prob. ATPase**	unknown	unknown	
**Not found in *L. tarentolae***		unknown	unknown	AQR	
		unknown	unknown	FAM32A	
		unknown	unknown	FAM50A	
		unknown	unknown	NOSIP	
		unknown	unknown	PPIE	
		unknown	unknown	PPIL3	
		unknown	CPR1/CPR3	PPWD1	
		unknown	Cwc2/Ecm2	RBM22	
		unknown	unknown	STEEP	
		unknown	unknown	TLS1	
		unknown	unknown	ESS2	

Supplementary Table 3. PCR primers and oligonucleotide probes used in this study.

Name	Sequence
F3001	5'- GATCTGGTTGATTCTGCCAGTAG -3'
A1715	5'- TATTCGTTGTCAGATGGCGCAC -3'
P1442	5'- CCGACTGCAACAAGGTGTAG -3'
A264	5'- CATCTATAGAGAAGTACACGTAAAAG -3'
SL snRNA outron probe	5'- Biotin - AGCGCCTGCGCGCTTCCAAAATCTTGC -3'
U2 snRNA probe	5'- Biotin - CCTTTGCCCGAAGGCAGTATCAGGAGTTACTCTG -3'
U4 snRNA probe	5'- Biotin - CCTGAGGTTCTTGCGTTCACATCTCCCTGCGC -3'
U5 snRNA probe	5'- Biotin - TAGGGTAGGGAGCGCTGCTTAGTAAAAGTCGAAGC -3'
U6 snRNA probe	5'- Biotin - GCCTTGCGCAGGGAGAGTGCTAATCTTCTCTGTTG -3'
NheI-PRP22_Fw	5'- GTCAGCTAGCATGAACGCTGTCGGCAGCAC -3'
NotI-PRP22_Rev	5'- GACTGCGGCCGCTCAACGCCGTTGCTTTTTAAGACGC -3'
NheI-PRP22ΔNTD_Fw	5'- CTCAGCTAGCGGTCTACCCGAGACGATGCAGAC -3'

Supplementary Table 4. Amino acid sequences of proteins not referenced on UniProt and TriTrypDB but identified in the cryo-EM structures.

Proteins detected in our cryo-EM reconstructions that are not annotated in either UniProt or TriTrypDB were manually built and further identified through motif matching and BLASTN searches using orthologs from *Trypanosoma* species.

Name	Sequence
RBP1	MVTVIRIRNLPATYSAEKLLRLCACFGEVTAFTAVGEEGAGGHSAATAD<br>VSFAEKSDALAAAQNMEGMEFDGVFLQVFVRQE
Sde2-like	MPAGLPRALCCTLLPVQKVISLTVLQATKDAILEALSKQTDSSPQTFELY<br>VKENNKLVALRENISSYDSIFALVRCRGGGKGGFRKQLEKKGRGFARAK<br>MKEKRNSTKAAPEEEKSQQKKCDTVAVKKRLAKAEPSTTRTLVKQGLA<br>FVINSFQRGNHA

Supplementary Table 5. Cryo-EM data collection and refinement statistics.

**Cryo-EM reconstructions**	Trans-splicing C* complex	Trans-splicing P complex	PRP22/DHX8 RNA-bound	PRP22/DHX8 open conformation
PDB IDs	PDB 9IF7	PDB 9IF8	PDB 9IBC	PDB 9IBD
EMDB IDs				
Composite maps	EMD-52843	EMD-52844		
Overall maps	EMD-52793	EMD-52799		
Focused map				
Core	EMD-52794	EMD-52800		
EJC	EMD-52795			
SNU114	EMD-52796	EMD-52802		
U5-40K	EMD-52797	EMD-52797		
U5 Sm ring	EMD-52806	EMD-52806		
U2 snRNP	EMD-52805	EMD-52805		
U6 Sm ring	EMD-52798	EMD-52798		
CWC22		EMD-52801		
NTC-1	EMD-52803	EMD-52803		
NTC-2	EMD-52804	EMD-52804		
NTC core	EMD-52807	EMD-52807		
PRP22/DHX8 closed conformation	EMD-52808	EMD-52808	EMD-52808	
PRP22/DHX8 open conformation				EMD-52809
**Data collection and processing**				
Microscope	Titan Krios	Titan Krios	Titan Krios	Titan Krios
Voltage (keV)	300	300	300	300
Camera	K3	K3	K3	K3
Magnification	64,000	64,000	64,000	64,000
Pixel size at detector (Å/pixel)	1.065	1.065	1.065	1.065
Total electron exposure (e-/Å2)	50	50	50	50
Exposure rate (e-/pixel/sec)	30	30	30	30
Number of frames collected (no.)	40	40	40	40
Defocus range (μm)	-0.5 to -2.5	-0.5 to -2.5	-0.5 to -2.5	-0.5 to -2.5
Energy filter slit width (V)	20	20	20	20
Automation software	SerialEM	SerialEM	SerialEM	SerialEM
Micrographs collected (no.)	31,410	31,410	31,410	31,410
Total extracted particles (no.)	1,214,398	1,214,398	1,214,398	1,214,398
**For each reconstruction**				
Final particle images (no.)	184,554	141,035	98,912	115,573
Point-group symmetry	C1	C1	C1	C1
Resolution (global, Å)*				
FSC 0.5 (unmasked/masked)	6.19/3.13	7.53/3.30	9.79/4.04	9.44/6.82
FSC 0.143 (unmasked/masked)	3.56/2.69	3.77/2.83	7.26/3.51	7.10/3.68
Resolution range (local at FSC 0.5, Å)	2.35-45.37	2.35-45.36	3.04/56.61	3.11-62.17
Map sharpening *B* factor ($Å^2$)				
Overall map	-64.36	-65.65		
Focused maps	-61.48 to -116.33	-60.83 to -116.33	-85.82	-95.67
**Model composition**				
Non-hydrogen atoms	135,855	128,394	9,503	9,227
Protein residues	17,475	16,581	1,174	1,192
RNA bases	332	319	7	0
Ligands (Mg^{2+}/Zn^{2+}/ K^{+}/ GTG/ADP/GTP/IHP/G5J)	12/5/1/ 1/1/1/1/1	12/4/0/ 1/0/1/1/1	0/0/0/ 0/0/0/0/0	0/0/0/ 0/0/0/0/0
**Model refinement**				
Refinement package (real space)	Phenix 1.19.1	Phenix 1.19.1	Phenix 1.19.1	Phenix 1.19.1
Nominal resolution (Å)	3.0	3.2	3.8	3.8
Map cross-correlation (within mask)	0.75	0.74	0.67	0.69
Model-to-map FSC, threshold 0.50 (masked/unmasked, Å)	2.98/2.98	3.32/3.32	3.96/4.13	4.06/4.17

**RMSDs**				
Bond lengths (Å)	0.005	0.007	0.004	0.004
Bond angles (°)	0.984	1.035	0.964	0.961
**B-factors**				
Average B-factors (A^2)				
Protein	72.49	94.13	91.92	126.78
RNA	78.52	95.23	96.38	
Ligand	52.88	59.29		
**Validation**				
Clash score	1.81	2.30	3.43	3.18
MolProbity score	1.36	1.45	1.13	1.11
CaBLAM outliers	0.67	0.78	0.88	0.69
Poor rotamers (%)	3.61	3.82	0.00	0.00
C-beta deviations	0.00	0.00	0.00	0.00
EMRinger score	3.11	2.87	1.51	1.37
**Ramachandran plot**				
Favored (%)	98.77	98.47	98.44	98.30
Allowed (%)	1.23	1.53	1.56	1.70
Outliers (%)	0.00	0.00	0.00	0.00
**RNA validation**				
Average suiteness (%)	57.0	55.1	25.0	
Good sugar puckers (%)	99.4	98.7	100	
