# Supplementary material for: Structural basis of step II spliced leader RNA trans-splicing in trypanosomatid parasites
Source: Nat Commun. 2026 Sep 23;17:9450. doi: 10.1038/s41467-026-77480-6 (PMC13601561; doi:10.1038/s41467-026-77480-6)
Supplement: Supplementary file 2 [file 41467_2026_77480_MOESM7_ESM.pdf]

nature portfolio

Corresponding author(s): Arnaud Vanden Broeck

Last updated by author(s): Jul 27, 2026

Reporting Summary

Nature Portfolio wishes to improve the reproducibility of the work that we publish. This form provides structure for consistency and transparency in reporting. For further information on Nature Portfolio policies, see our Editorial Policies and the Editorial Policy Checklist.

Statistics

For all statistical analyses, confirm that the following items are present in the figure legend, table legend, main text, or Methods section.

n/a	Confirmed	
☐	☒	The exact sample size (n) for each experimental group/condition, given as a discrete number and unit of measurement
☐	☒	A statement on whether measurements were taken from distinct samples or whether the same sample was measured repeatedly
☒	☐	The statistical test(s) used AND whether they are one- or two-sided<br>*Only common tests should be described solely by name; describe more complex techniques in the Methods section.*
☒	☐	A description of all covariates tested
☒	☐	A description of any assumptions or corrections, such as tests of normality and adjustment for multiple comparisons
☐	☒	A full description of the statistical parameters including central tendency (e.g. means) or other basic estimates (e.g. regression coefficient) AND variation (e.g. standard deviation) or associated estimates of uncertainty (e.g. confidence intervals)
☒	☐	For null hypothesis testing, the test statistic (e.g. F, t, r) with confidence intervals, effect sizes, degrees of freedom and P value noted<br>*Give P values as exact values whenever suitable.*
☒	☐	For Bayesian analysis, information on the choice of priors and Markov chain Monte Carlo settings
☒	☐	For hierarchical and complex designs, identification of the appropriate level for tests and full reporting of outcomes
☒	☐	Estimates of effect sizes (e.g. Cohen's d, Pearson's r), indicating how they were calculated

Our web collection on statistics for biologists contains articles on many of the points above.

Software and code

Policy information about availability of computer code

Data collection	Cryo-EM datasets were collected using SerialEM 4.1 (https://bio3d.colorado.edu/SerialEM/) on an FEI Titan Krios (FEI/Thermofisher) microscope using an energy filter at a slit width of 20 eV operating at 300 kV and 64,000x magnification. Two datasets were collected at super-resolution pixel size of 0.54 Å/px, with defocus values ranging from -1 to -2.5 µm.
Data analysis	All software used for data analysis in this study were available online:<br>1. RELION 5.0 (http://www2.mrc-lmb.cam.ac.uk/relion): Cryo-EM data analysis<br>2. cryoSPARC v4 (https://cryosparc.com): Cryo-EM data analysis<br>3. Coot 0.9 (https://www2.mrc-lmb.cam.ac.uk/ personal/pemsley/coot/): Model building<br>4. Phenix 1.19 (https://www.phenix-online.org): Model refinement<br>5. UCSF Chimera X 1.10 (https://www.cgl.ucsf.edu/chimera/): Density maps and structural models<br>6. PyMol 3 (https://www.pymol.org): structural models<br>7. GraphPad Prism 11.0.0 (https://www.graphpad.com/)<br>8. Fiji 2.18 (https://imagej.net/software/fiji/)

For manuscripts utilizing custom algorithms or software that are central to the research but not yet described in published literature, software must be made available to editors and reviewers. We strongly encourage code deposition in a community repository (e.g. GitHub). See the Nature Portfolio guidelines for submitting code & software for further information.

Data

Policy information about availability of data

All manuscripts must include a data availability statement. This statement should provide the following information, where applicable:

- Accession codes, unique identifiers, or web links for publicly available datasets
- A description of any restrictions on data availability
- For clinical datasets or third party data, please ensure that the statement adheres to our policy

Raw unaligned multi-frame movies and consensus polished particles stack have been deposited in the Electron Microscopy Public Image Archive (EMPIAR-13613). The composite cryo-EM maps and atomic models have been deposited in the Electron Microscopy Data Bank (EMDB) and the Protein Data Bank (PDB), respectively: trans-C* complex (EMD-52843, PDB 9IF7), trans-P complex (EMD-52844, PDB 9IF8), RNA-bound PRP22 in the closed conformation (EMD-52808, PDB 9IBC), and PRP22 in the open conformation (EMD-52809, PDB 9IBD). All overall and focused refinement maps have been deposited in the EMDB under accession numbers EMD-52794 to EMD-52809. The following atomic models were used for structural comparison and figure generation: 6SH7, 7W5A, 8C6J, 9DTR and 9FMD.

Research involving human participants, their data, or biological material

Policy information about studies with human participants or human data. See also policy information about sex, gender (identity/presentation), and sexual orientation and race, ethnicity and racism.

Reporting on sex and gender	n/a
Reporting on race, ethnicity, or other socially relevant groupings	n/a
Population characteristics	n/a
Recruitment	n/a
Ethics oversight	n/a

Note that full information on the approval of the study protocol must also be provided in the manuscript.

Field-specific reporting

Please select the one below that is the best fit for your research. If you are not sure, read the appropriate sections before making your selection.

☒ Life sciences ☐ Behavioural & social sciences ☐ Ecological, evolutionary & environmental sciences

For a reference copy of the document with all sections, see nature.com/documents/nr-reporting-summary-flat.pdf

Life sciences study design

All studies must disclose on these points even when the disclosure is negative.

Sample size	Sample sizes for cryo-EM datasets were not predetermined. 31,524 movies were collected. The number of particles extracted from these micrographs were not predetermined. For kinetic analyses, ATP hydrolysis rates were measured at saturating concentration of ATP for all samples. Kcat values were determined using a single ATP concentration at least tenfold higher than the Km.
Data exclusions	For cryo-EM analyses, poorly aligned, damaged, or contaminating particles were excluded during image processing prior to final reconstructions. No data were excluded from the functional assays.
Replication	For the ATPase assays, three technical replicates were performed for each data point (n = 3). During the revision, we repeated the ATPase assays in two independent biological replicates and obtained consistent results. No findings reported in this study failed to be reproduced.
Randomization	For cryo-EM analyses, particles were randomly divided into two independent half-sets for gold-standard refinement. These half-sets were refined independently and compared to estimate resolution.
Blinding	Blinding was not applicable to this study. In single-particle cryo-EM analysis, particles are randomly divided into two independent half-sets for refinement, ensuring unbiased resolution estimation.

Reporting for specific materials, systems and methods

We require information from authors about some types of materials, experimental systems and methods used in many studies. Here, indicate whether each material, system or method listed is relevant to your study. If you are not sure if a list item applies to your research, read the appropriate section before selecting a response.

Materials & experimental systems

n/a	Involved in the study
☐	☒ Antibodies
☐	☒ Eukaryotic cell lines
☒	☐ Palaeontology and archaeology
☒	☐ Animals and other organisms
☒	☐ Clinical data
☒	☐ Dual use research of concern
☒	☐ Plants

Methods

n/a	Involved in the study
☒	☐ ChIP-seq
☒	☐ Flow cytometry
☒	☐ MRI-based neuroimaging

Antibodies

Antibodies used

Anti-HA antibody, Mouse monoclonal (Sigma, H3663) - dilution 1:5000
HRP-conjugated secondary antibody rabbit anti-mouse IgG (Abcam, ab6728) - dilution 1:10000

Validation

Monoclonal Anti-HA antibody produced in mouse (Sigma, H3663) has been validated for and used in immunoblotting, immunocytochemistry and immunoprecipitation.

Rabbit anti-Mouse IgG H&L (HRP) (ab6728) is a horseradish peroxidase (HRP) conjugated secondary antibody suitable for both chemiluminescent and chromogenic detection. Ideal for Western blot, ELISA and IHC applications.

Eukaryotic cell lines

Policy information about cell lines and Sex and Gender in Research

Cell line source(s)

Leishmania tarentolae Parrot P10 ATCC 30267 obtained from Jena Bioscence

Authentication

The cell line was not authenticated

Mycoplasma contamination

Cell lines were not tested for mycoplasma contamination.

Commonly misidentified lines
(See ICLAC register)

No commonly misidentified cell lines were used in the study.

Plants

Seed stocks

n/a

Novel plant genotypes

n/a

Authentication

n/a
