# Supplementary material for: Structural basis of step II spliced leader RNA trans-splicing in trypanosomatid parasites
Source: Nat Commun. 2026 Sep 23;17:9450. doi: 10.1038/s41467-026-77480-6 (PMC13601561; doi:10.1038/s41467-026-77480-6)
Supplement: Supplementary file 3 [file 41467_2026_77480_MOESM8_ESM.pdf]

nature portfolio

Peer Review File

Structural basis of step II Spliced Leader RNA trans-splicing in trypanosomatid parasites

Corresponding Author: Dr Arnaud Vanden Broeck

This file contains all reviewer reports in order by version, followed by all author rebuttals in order by version.

Version 0:

Reviewer comments:

Reviewer #1

(Remarks to the Author)
This article presents structural studies of the trypanosomatid trans-spliceosome during exon ligation and appears to provide the first structural characterization of assembled trans-spliceosomes. Two spliceosomal intermediates are described: the C* complex, primed for exon ligation, and the post-catalytic P complex. The cryo-EM data processing and model building are generally of high quality, with acceptable refinement statistics indicative of a well-conducted study. The manuscript is accompanied by useful PyMOL sessions and the most relevant EM maps.
Despite its clear novelty, the manuscript at times lacks clarity in distinguishing features that are specific to the trans-spliceosome from those already well established in cis-splicing systems. Consequently, the biological insights unique to trans-splicing are not sufficiently emphasized. In addition, certain aspects of the work rely on interpretations that are either questionable or insufficiently explained. Finally, the interest of the "broad pairwise protein–protein interaction (PPI) screen using AlphaFold2-multimer" is unclear and contributes to making what should be the main message(s) of the paper less evident.
Overall, this study is of definite interest but would benefit from more conceptual and clearer analysis. In addition some structural interpretations made need clarification.
Below are my specific comments.
The authors used CDC5L as an affinity handle to purify catalytic spliceosomes. Similar approaches have previously been employed to capture multiple intermediates of the yeast spliceosome (Cef1 was used in that case). CDC5L is a core component of the NTC, which is recruited upon spliceosome activation and is expected to associate with the spliceosome from the Bact stage onward. This raises the question of whether the strategy presented here selectively captured only the C* and P complexes. Could the authors clarify if other splicing intermediates, such as earlier Bact or C complexes, or later intermediates such as the ILS, were also present in the collected datasets? If they were absent, would that suggest differences in the kinetics of recruitment of the NTC/active site formation between trypanosomatid and opisthokonts? While the presence of Y-branched pre-mRNA largely supports completion of the first catalytic step, some "intact" SL RNA is still visible in Supplementary Fig. 1F (if my interpretation is correct and indeed the "SLint" corresponds to a probe against the SLoutron) and given the apparent heterogeneity of pre-mRNAs captured it seems difficult to exclude the presence of step I spliceosomes on biochemistry alone.
If relevant, I would like to encourage the authors to be as transparent as possible regarding how the ab initio reconstructions were performed and the rationale for classes selection from 3D classification. In particular, the map representing ~21% of the particles shows a reasonably well-defined structure, albeit with weaker density (possibly corresponding to the SL RNP ring). Could this class represent a later spliceosome intermediate undergoing disassembly? There also appears to be an inconsistency in particle numbers: in Supplementary Fig. 3 ("Data cleanup 2"), 47% of 545,945 particles does not correspond to 181,522 particles. Please clarify. Finally, from their MS analysis, the author mention "72 were within two orders of magnitude in abundance relative to the bait protein CDC5L (Supplementary Data 1). Among these, we identified 52 proteins associated with the late stages of SL trans-splicing". Could the author also discuss what are the 20 other factors? Were factors specific to distinct spliceosome states also detected?

Regarding the section entitled "Trypanosomatid-specific regulation of the DEAH-box helicase PRP22/DHX8", the authors initially appear very cautious when describing model building of the putative helicase N-ter but much less so when drawing biological conclusions regarding DDX41 (in this part and in discussion). Could they elaborate on the reasons for this assignment? The authors should clarify the evidence supporting this interpretation (for example, by providing representative density for the most distinctive features of the modeled peptide) and harmonize the degree of certainty in their interpretation

throughout the text.

P10-5: “Proteins typically associated with intron stabilization, such as RBM22 (Cwc2/Ecm2 in yeast) and the AQR helicase, are absent, reflecting structural adaptations to SL trans-splicing. RBM22 is replaced by the Sm ring of the SL snRNP, and in the absence of AQR helicase, the U6 LSm ring occupies its position on SYF1.” In yeast and human release of LSm ring of U6 occurs during Bact formation and is thought to be an important marker for spliceosome activation. This is not adequately discussed in the paper.

P10-l10 : “Strikingly, the branch helix, formed by base pairing between the pre-mRNA and U2 snRNA, is not visible, and nucleotides surrounding the branchpoint remain flexible, consistent with the absence of a conserved branchpoint motif in trypanosomatids and reinforcing the view that branchpoint selection is instead primarily guided by the downstream polypyrimidine tract” The link between the flexible nature of the branch and the low degree of conservation of the contacts is questionable since a similar argument could be brought forward in human where the branch sequence is very variable but well visible in the cryo-EM structures.

P10” with unique RNA-protein interactions that compensate for the absence of canonical introns and a branchpoint recognition motif, while preserving the fundamental chemistry of exon ligation.” Is probably true but the text fails to clearly convey the idea by giving concrete examples.

The section describing the EJC is somewhat confusing and needs clarification/reinterpretation.
On p.8, l.10, the authors state: “A key distinction at the protein level between the trans-C and trans-P complexes is the release of the EJC and the destabilization of the step II factors CACTIN, SLU7, and PRP18 following exon ligation, as similarly observed in the yeast P complex.” The term “protein level” is misleading, as it suggests biochemical evidence, whereas the conclusions presented here are derived exclusively from structural observations.
More importantly, it remains unclear how the authors interpret the fate of the EJC between the C* and P states. If the EJC undergoes “detachment” (p11 and discussion) during this transition, what precisely is meant by it remaining “tethered” (Supplementary Fig. 7B; Discussion, p.16)? The EJC is also depicted in Fig. 2B as binding the 5′ exon. Finally, the statement in the Discussion that “Through its NTE, PRP18 transmits these events to the EJC, which detaches but remains tethered to the trans-spliceosome via the SL exon” is particularly intriguing and difficult to reconcile with the structural data. If I understood correctly, the authors appear to propose that the EJC remains clamped onto the 5′ exon after dissociating from Cwc22. However, inspection of the cryo-EM densities argues against this interpretation: there is no residual density in the region where the EJC would be expected in the P complex, and given the length of the RNA protruding from the spliceosome in this region, an EJC still bound to the RNA should remain at least partially ordered and thus partially visible. An alternative explanation would be that the EJC dissociates entirely, which would be a surprising and potentially important finding.
I therefore encourage the authors to clarify their interpretation of the EJC state between the C* and P complexes and to reconcile it more explicitly with the observed densities. In addition, further discussion of prior work on DEAD-box helicase-mediated RNA clamping, and how nucleotide state influences RNA engagement and release, would greatly strengthen this section and help contextualize the proposed mechanism in line with their observation that ADP and not ATP would be bound to eIF4AIII...
P11 “We also identified the extended N-terminus of PRP18 forming direct contacts with the CWC22 NTD, thereby coupling SL exon binding by the EJC with SL exon ligation sensing through the PRP18 insertion loop into the catalytic site”. Regardless of the comment above, this is an appealing hypothesis. Could the author justify better their decision to attribute this density to Prp18, could they show some density supporting this attribution?

Along the text, which is generally rather condensed, some statements are repeated several times. For example, p10 l5 “RBM22 is replaced by the Sm ring of the SL snRNP, and in the absence of AQR helicase, the U6 LSm ring occupies its position on SYF1” and p7 l20 “It protrudes outward, positioned atop BUD31, occupying the site normally held by intron-bound RBM22 in human spliceosomes or Cwc2/Ecm2 in yeast spliceosomes” and p7 l10 “U6 snRNP is composed of U6 snRNA and a heptameric LSm ring (LSm2–8), which is clearly resolved and docked onto the Nineteen Complex (NTC), occupying the site typically held by the intron-stabilizing AQR helicase in human spliceosomes” and p13 l5 “the LSm ring docks on SYF1 in a position normally occupied by the intron-binding helicase AQR in metazoans, creating a unique conformation of U2/U6 helix II”.

Reviewer #2

(Remarks to the Author)
Trypanosomatids such as Trypanosomes and Leishmania employ a unique trans-splicing mechanism. Their genes were transcribed in a single polycistronic pre-mRNA and splicing of each gene occurs in trans with a splice-leader (SL) RNA. As a result, each resulting pre-mRNA has the same splice-leader exon at their 5' end.

In this paper, the authors provided the first insight into this SL-trans-splicing process. They tagged a late-stage splicing subunit in Leishmania, purified the complex, characterised its composition and potentially protein-protein interactions using AlphaFold. They then determined the structures of the purified complex and resolved the structures of the C* (prior to second step of splicing) and P (post splicing). Through focused classification/refinement they obtained higher resolution maps at different flexible parts of the structures, yielding a composite maps. The structures revealed a conserved core that is highly similar to the canonical cis-splicing complexes. They then discussed several features of the structures: 1) SL RNA recognition by the Exon Junction Complex (EJC), which binds at exon-intron junctions and marks the RNA for non-sense

mediated decay (NMD). 2) Trypanosome-specific proteins or extensions that have been adapted to the shortening of the snRNAs. 3) the RNA helicase Prp22, which release the SL capped pre-mRNA from the trans-spliceosome.

Generally, the strength of the paper is that it provides the first structural insights into SL-trans-splicing. It will be of broad interest to people in both the splicing field and parasite biology. The cryo-EM analysis is done nicely. On the other hand, there are also some weaknesses. There is no functional data to support any key interactions observed and to elucidate the roles of some of trypanosome-specific factors. As a result, the paper contains mostly detailed descriptions but it lacks deep analyses of how their observations relate to the unique mechanism of trans-splicing. Some of the suggested functional implications of the structural observations are purely speculative. Therefore, while I think it is a nice piece of work, addition of functional work will be critical to validate some of the structural observation and elevate the impact of the paper.

Below are specific major comments:

1. Page 8, line 7. The authors identified a protein that they called "putative" RNA helicase and suggested that it could be a functional analog of human DDX41. With only 10% sequence identity, they should provide evidence including helicase assay and ATPase assay to support this helicase assignment. Similarly, for the DDX41 analog assignment, functional data should be required.
2. Page 9 Line 20: for the following claim "G39 is likely stochastically flipping in and out until the second trans-esterification reaction occurs. This dynamic conformation is enabled by the absence of a FAM32 ortholog in trypanosomatids", the authors should provide more explanations and make a figure comparing with other spliceosome structures containing FAM32.
3. Page 12, lined 11-14. The authors made an interesting observation about the nucleotide binding state of the eIF4A3 and suggested that previous structures may have mis-interpreted it. How can they rule out that species-specific difference could account for the difference in the nucleotide state? Or the sub-state of the spliceosome in their structure may be different from those reported and could account for such difference. Additional evidence to back their suggestion should be provided to rule out these possibilities.
4. They also proposed a "locking and docking" mechanism for eIF4A3 without additional evidence. Either experiments are performed to show this or they should explain why experiments are not feasible.
5. Page 14, lines 2-6. These descriptions lack a figure to go with it. What do the authors mean by "activated"? Activated for what? Where is the evidence for this "activation" of SDE2-like protein?
6. Page 14, lines 6-7, line 11. the suggested roles of this SDE2-like protein and LtaPh1601800 are speculative without evidence. Evidence the authors could provide includes expression of mutant proteins (either on top of wild-type protein or in addition in case this could act as a nominant negative mutant) and examine the outcome of RNA splicing via sequencing.
7. Page 15, lines 7-9. Similar to the point above, there is no provided evidence for the role of the linker region of PRP22 in regulating PRP22 itself. Either expression of Prp22 and test its RNA binding/helicase activity in vitro with mutation at this linker would be required to support this statement.
8. Figure S3. 3D classification without alignment, please provide T-value used.
9. Figure S4. The trans-P complex, gold-standard FSC is 2.8 and model-vs-map FSC is 3.3. There is a large gap between these resolutions, suggesting overfitting in the model refinement. The authors should re-refine the structure to make sure this issue is fixed.
Also number labels in this figure are very small and difficult to see even. Please enlarge them for visibility.
10. Figure S5. The SL SM ring and Brr2 density shown are poor, raising the question of the confidence of the fitting. The authors need to explain what procedure they used for the current fitting of SL Sm ring and BRR2-Prp8 and report confidence scores of the fitting, and maybe present other possible fittings.

Minor comments:
11. Page 2 Line 10: "in silico" should be italic.
12. Page 8, line 14. Fig. 1B, C have no comparison with the canonical spliceosome.
13. Page 9 Line 2: label "loop I" in Figure 2.
14. 3'-splice site abbreviation is not defined.
15. Page 9, lines 3 and 11, referrals are not specific and very difficult for readers to find which figures refer to which description. Figure S12 seems to be out of order.
16. Figure 1B. The legend reads Trypanosome-specific proteins but the figure has proteins that are not trypanosome-specific. It makes it a bit unclear.
17. Figure 2D, the snRNAs are very transparent. It would be helpful to make them easier to see.

Reviewer #3

(Remarks to the Author)
I co-reviewed this manuscript with one of the reviewers who provided the listed reports. This is part of the Nature Communications initiative to facilitate training in peer review and to provide appropriate recognition for Early Career Researchers who co-review manuscripts.

Version 1:

Reviewer comments:

Reviewer #1

(Remarks to the Author)
The revised manuscript adresses my concerns.

Reviewer #2

(Remarks to the Author)
In the revised manuscript and the associated response, the authors have clarified several points that were previously unclear. They addressed most comments about the lack of functional data through removing functional claims rather than functional experiments. Nonetheless, they provided ATPase assays for the “helicase” assignments in response to Points 1 and 7. I found the responses are mostly satisfactory, except for two remaining concerns, particularly in the additional experiments provided, as outlined below.

1. The purity of Prp22 protein (both full-length and truncated) subjected to the ATPase assays is poor with many additional bands unannotated. That of the ATPase in point 1 is better but still there are faint bands at ~100 and 70 KDa. Those contaminants, for example Hsp proteins, can also have ATPase activities themselves, which would affect the results. They also affect protein concentration calculating, leading to incorrect estimation of enzyme kinetic parameters. To avoid such potential problems, the authors should consider purifying the protein further for these assays. This would be the preferred option. Otherwise, they should identify the bands to make sure they themselves do not have ATPase activity or include a negative control, in which they perform similar pulldowns with lysates from cells not integrated with the GFP proteins and subject the elution to ATPase assays. If those are common contaminants, they would come down in the elution yet do not contribute to the ATPase activity observed.

Given that these are the only additional experiments provided in this revised version, I strongly recommend the authors to consider the point below and provide more rigorous controls to ensure the validity of the data.

2. Figure S4. The composite map is not proper for calculating model-to-map FSC. The author should use a refinement map resulting from a cryo-EM refinement for this purpose.

Reviewer #3

(Remarks to the Author)
I co-reviewed this manuscript with one of the reviewers who provided the listed reports. This is part of the Nature Communications initiative to facilitate training in peer review and to provide appropriate recognition for Early Career Researchers who co-review manuscripts.

Version 2:

Reviewer comments:

Reviewer #2

(Remarks to the Author)
In this second revision, the authors have done an excellent job in addressing the issues we raised about of the protein purity and FSC calculations in their previous revision. I am satisfied with this revision and can recommend the work for publication.

Reviewer #3

(Remarks to the Author)
I co-reviewed this manuscript with one of the reviewers who provided the listed reports. This is part of the Nature Communications initiative to facilitate training in peer review and to provide appropriate recognition for Early Career Researchers who co-review manuscripts.

Open Access This Peer Review File is licensed under a Creative Commons Attribution 4.0 International License, which permits use, sharing, adaptation, distribution and reproduction in any medium or format, as long as you give appropriate credit to the original author(s) and the source, provide a link to the Creative Commons license, and indicate if changes were made.
In cases where reviewers are anonymous, credit should be given to 'Anonymous Referee' and the source.
The images or other third party material in this Peer Review File are included in the article's Creative Commons license, unless indicated otherwise in a credit line to the material. If material is not included in the article's Creative Commons license and your intended use is not permitted by statutory regulation or exceeds the permitted use, you will need to obtain permission directly from the copyright holder.
To view a copy of this license, visit https://creativecommons.org/licenses/by/4.0/

<u>Point by point response to reviewers' comments</u>

We thank all the reviewers for the time they have taken to carefully review our work and for their comments and suggestions, which have helped us to significantly improve our manuscript. We address each reviewer comments below (shown in black), with our responses in blue and the modified text from the main manuscript or supplementary information in red. The revised manuscript is also provided with track changes. Newly added text is highlighted in gray.

Reviewer #1 (Remarks to the Author)

This article presents structural studies of the trypanosomatid trans-spliceosome during exon ligation and appears to provide the first structural characterization of assembled trans-spliceosomes. Two spliceosomal intermediates are described: the C* complex, primed for exon ligation, and the post-catalytic P complex. The cryo-EM data processing and model building are generally of high quality, with acceptable refinement statistics indicative of a well-conducted study. The manuscript is accompanied by useful PyMOL sessions and the most relevant EM maps.

Despite its clear novelty, the manuscript at times lacks clarity in distinguishing features that are specific to the trans-spliceosome from those already well established in cis-splicing systems. Consequently, the biological insights unique to trans-splicing are not sufficiently emphasized. In addition, certain aspects of the work rely on interpretations that are either questionable or insufficiently explained. Finally, the interest of the "broad pairwise protein–protein interaction (PPI) screen using AlphaFold2-multimer" is unclear and contributes to making what should be the main message(s) of the paper less evident.

Overall, this study is of definite interest but would benefit from more conceptual and clearer analysis. In addition some structural interpretations made need clarification.

We thank the reviewer for their constructive feedback and for the careful evaluation of our manuscript. We appreciate the positive assessment of the novelty and interest of our work. We have now substantially revised the manuscript to provide a clearer conceptual analysis of our findings. In particular, we more explicitly distinguish features that are unique to SL trans-splicing from those that are shared with canonical cis-splicing systems in opisthokonts, as well as trypanosomatid-specific adaptations. We have also clarified or toned down several interpretations that were previously insufficiently explained. In addition, we have restructured the presentation of the PPI screen to better highlight its relevance and have moved this section to a more appropriate place in the manuscript. Finally, we have addressed all reviewer's specific comments below.

Below are my specific comments.

1. The authors used CDC5L as an affinity handle to purify catalytic spliceosomes. Similar approaches have previously been employed to capture multiple intermediates of the yeast spliceosome (Cef1 was used in that case). CDC5L is a core component of the NTC, which is recruited upon spliceosome activation and is expected to associate with the spliceosome from the Bact stage onward. This raises the question of whether the strategy presented here selectively captured only the C* and P complexes. Could the authors clarify if other splicing intermediates, such as earlier Bact or C complexes, or later intermediates such as the ILS, were also present in the collected datasets?

   We thank the reviewer for this helpful comment, which gives us the opportunity to clarify this point in the manuscript. By using CDC5L as an affinity handle, our goal was indeed to purify all activated trans-spliceosomal intermediates from the B^{act} stage to the Y-structure trans-spliceosome (YST; equivalent to the ILS in cis-splicing). However, using this strategy we were only able to biochemically isolate step II intermediates, specifically the trans-C* and trans-P complexes. This conclusion is supported by our downstream biochemical, mass spectrometry and cryo-EM analyses, which we address in response to the reviewers' specific comments below.

   If they were absent, would that suggest differences in the kinetics of recruitment of the NTC/active site formation between trypanosomatid and opisthokonts?

   We agree that the absence of B^{act}, step I, or YST intermediates in our datasets suggests potential kinetic differences compared to the yeast system. Specifically, activation/active site formation and step I may proceed more rapidly than step II, which could represent rate-limiting intermediates. Furthermore, the absence of the YST in our data could reinforce the notion that step II intermediates are rate-limiting, although we cannot exclude the possibility that the NTC is absent in the YST in trypanosomatids. Notably, a similar approach to ours was used by Lu *et al*. who tagged CDC5L or PRP19 (another NTC core factor) in *C. reinhardtii* and likewise isolated only C* complexes[1] . Similarly, Vorländer *et al*. reported cryo-EM structures of *C. elegans* ILS intermediates by tagging PRP19[2] . In their study, no other states could be identified using this bait, despite PRP19 being present from B^{act} to ILS in *C. elegans*, further supporting the notion of species-specific spliceosomal kinetics. Another possibility not to exclude is that the trans-splicing reaction continues during purification and accumulates step II complexes.

   While the presence of Y-branched pre-mRNA largely supports completion of the first catalytic step, some "intact" SL RNA is still visible in Supplementary Fig. 1F (if my interpretation is correct and indeed the "SLint" corresponds to a probe against the SLoutron) and given the apparent heterogeneity of pre-mRNAs captured it seems difficult to exclude the presence of step I spliceosomes on biochemistry alone.

   The reviewer's interpretation is correct: SL_{int} corresponds to the probe against the SL outron, which we have now corrected in the figure to SL_{outron}. Using northern blotting with the SL_{outron} probe, we indeed observed mainly Y-branched pre-mRNA, with only a small fraction of intact SL RNA relative to the Y-branched signal, consistent with a minor population of step I trans-

spliceosomes in our sample. However, the SYBR Green-stained RNA gel shows almost no signal for intact SL RNA compared to the other snRNAs, further supporting that step I trans-spliceosomes are rare in the purified sample. We have now clarified this point in the manuscript (see text below in red).

"The predominance of Y-branched endogenous pre-mRNAs, together with only a minor amount of intact SL RNA, indicates that the first transesterification reaction (step I) has largely occurred and that the purified complexes mainly correspond to step II trans-spliceosomes (Supplementary Fig. 1d-f)."

If relevant, I would like to encourage the authors to be as transparent as possible regarding how the ab initio reconstructions were performed and the rationale for classes selection from 3D classification.

Throughout data processing, we carefully inspected all classes to ensure that no relevant spliceosomal states other than trans-C* and trans-P were overlooked. However, we were only able to identify these two states. During the cleanup of dataset 1, we observed strong preferential particle orientation and the presence of damaged particles, contributing to anisotropic reconstructions. This required an additional round of 3D classification without alignment to remove these particles (Dataset 1, data cleanup 2) (see figure below). Dataset 2, obtained from the same sample but from a different vitrified grid, did not exhibit this problem, as the thicker ice layer mitigated the preferential orientation issue and thus did not require an additional round of 3D classification.

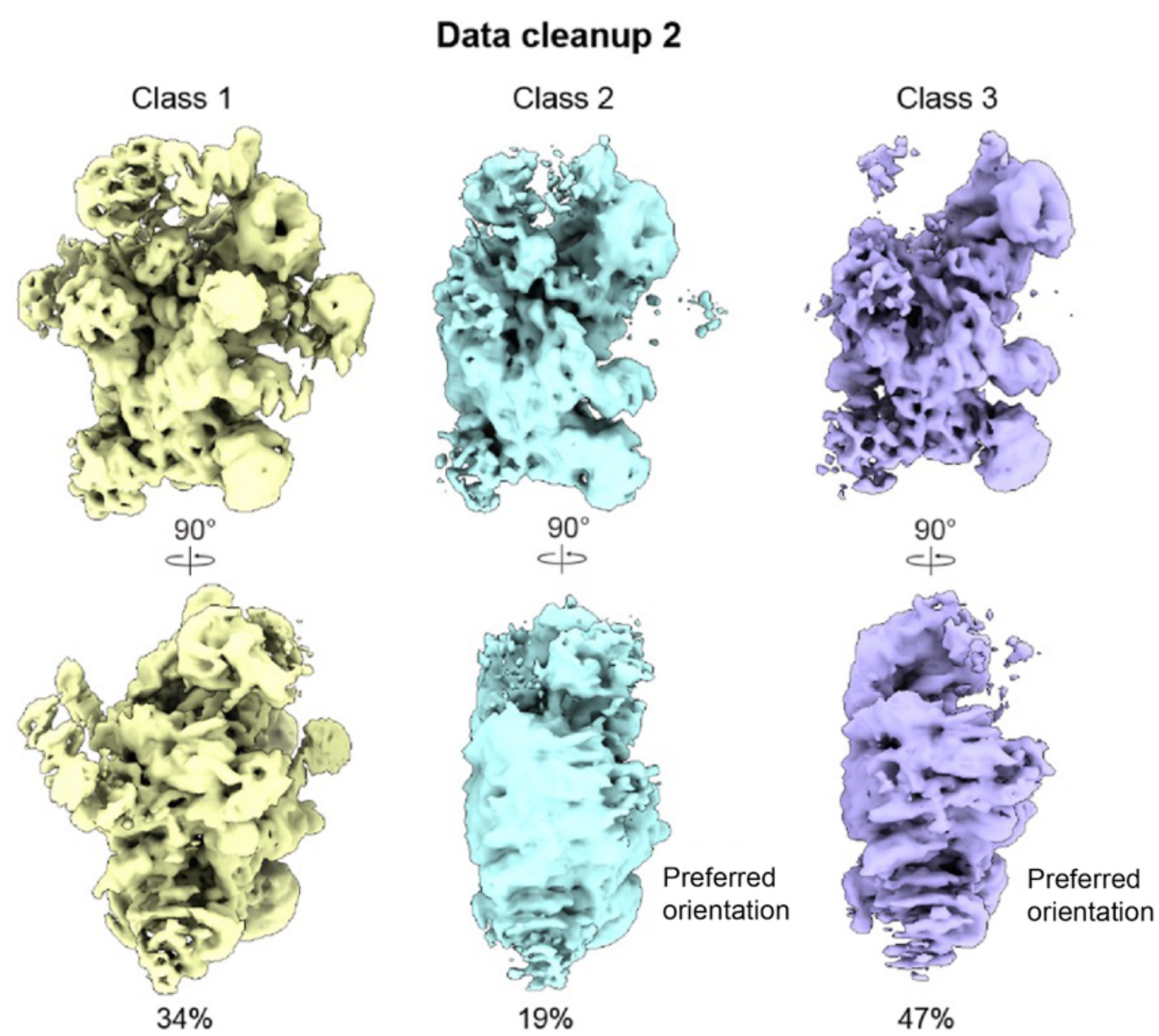

We have now included additional details in the Methods section and Supplementary Fig. 2 regarding the ab initio reconstruction procedure and the criteria used for class selection during 3D classifications (see text below in red).

"Particles from each dataset were extracted at a pixel size of 4.32 Å and initially cleaned using 2D classification. For dataset 1, ab-initio reconstruction was performed with two classes and standard parameters, except that the maximum resolution was set to 10 Å. The resulting ab-initio models included one "good" class resembling a trans-spliceosome and one "bad" class containing "junk" particles. These models were then used as initial references for both datasets (1x good class and 5x bad class) for further cleaning through heterogeneous refinement (Data cleanup 1). For dataset 1, an additional round of 3D classification without alignment (T-value = 4) was performed to remove particles contributing to anisotropic reconstructions (Data cleanup 2). For each dataset, after eliminating as many "junk" particles as possible, the particles contributing to the reconstruction of a defined trans-spliceosome were selected, re-extracted at a pixel size of 1.08 Å and merged into a stack of 571,611 particles."

In particular, the map representing ~21% of the particles shows a reasonably well-defined structure, albeit with weaker density (possibly corresponding to the SL RNP ring). Could this class represent a later spliceosome intermediate undergoing disassembly?

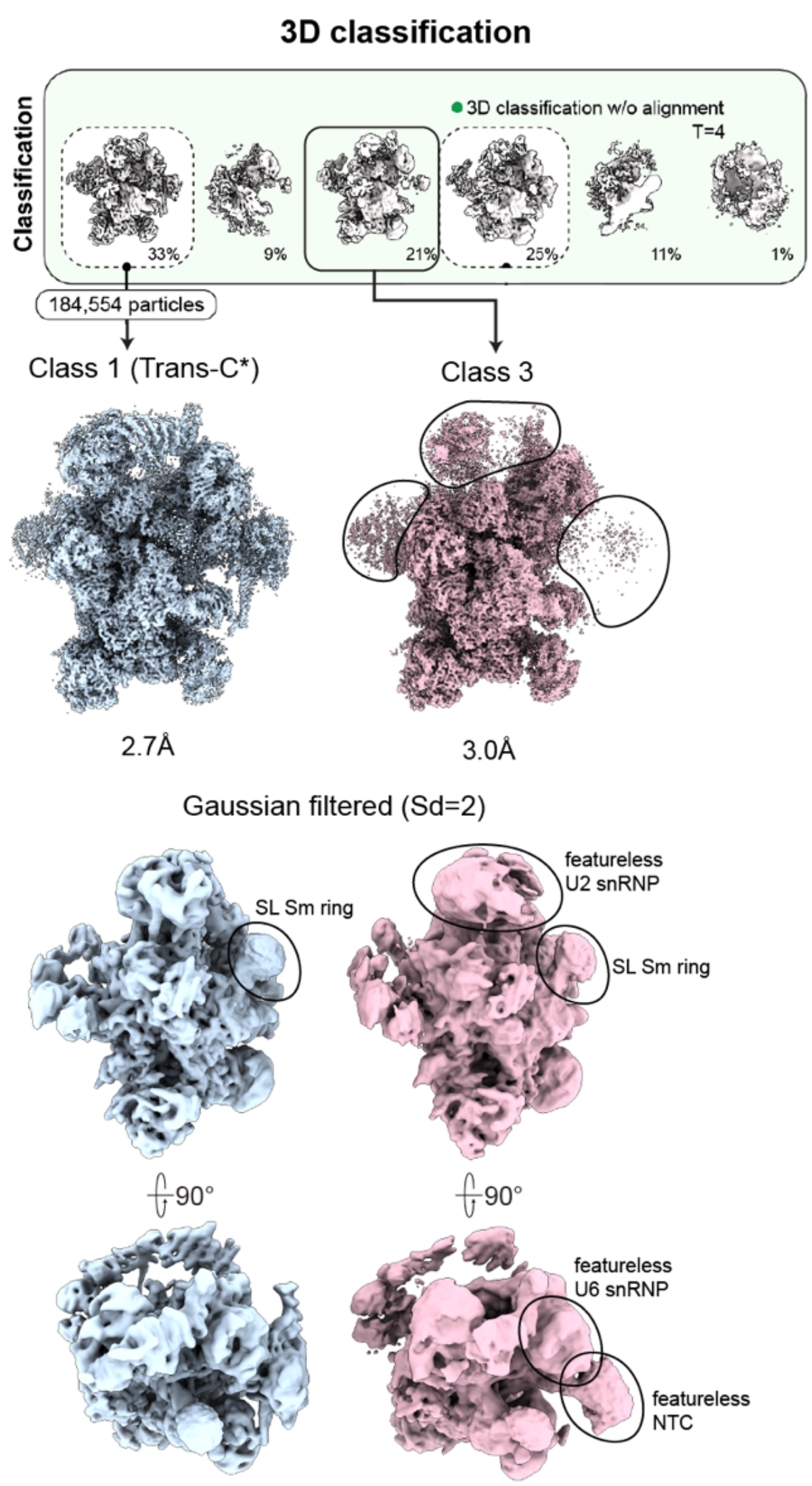

Following the merging of the two datasets, 3D classification yielded indeed a map representing 21% of the particles (class 3). After inspection of the 3D refinement, this class corresponds to a lower-resolution reconstruction of the trans-C* state and lacked defined features of the U2/U6 snRNPs and NTC subcomplexes (see figure on the left). Beyond this, no specific features supported assignment of this class to a disassembling trans-spliceosome. We therefore excluded these particles from the final trans-C* refinement. For clarity, this analysis is not shown in the manuscript, but a brief explanation was added to the Supplementary Fig. 2.

There also appears to be an inconsistency in particle numbers: in Supplementary Fig. 3 ("Data cleanup 2"), 47% of 545,945 particles does not correspond to 181,522 particles. Please clarify.

We thank the reviewer for spotting this discrepancy. We simply inverted the 47% with 34% in the figure. Class 1 represents 34% (185,522) of the particles and class 3 represents 47%.

Finally, from their MS analysis, the author mention "72 were within two orders of magnitude in abundance relative to the bait protein CDC5L (Supplementary Data 1). Among these, we identified 52 proteins associated with the late stages of SL trans-splicing". Could the author also discuss what are the 20 other factors? Were factors specific to distinct spliceosome states also detected?

The 20 other factors include either clear contaminants (e.g., ribosomal protein S6, intraflagellar transport (IFT) proteins, α/β-tubulins, histone subunits), proteins known to associate broadly with spliceosomes (e.g., HSP70 and HSP70-related chaperones), RNA-binding proteins (e.g., C3H1 zinc-finger proteins, R3H domain-containing proteins), as well as proteins of unknown function that were not visible in our cryo-EM reconstructions and for which we could not identify orthologs in opisthokonts using BLAST or Foldseek searches. Notably, we also did not observe any strong predicted interactions through our AlphaFold-Multimer–based PPI screen between these 20 factors and the proteins identified in the step II complexes (Supplementary Fig. 6).

Consistent with our biochemical data showing predominantly step II SL trans-spliceosomes and with our cryo-EM analysis, in which only trans-C* and trans-P complexes were identified, we did not detect or enrich factors characteristic of other trans-spliceosome intermediates. For example, we did not detect SF3B1, expected in B^{act} complexes, nor helicases corresponding to PRP2 (B^{act}), PRP16 (C), or DHX15 (ILS). We have now streamlined this section in the main text (see text below in red).

"To complement this observation at the protein level, we performed mass spectrometry analysis of the purified sample (Supplementary Data 1). This revealed the presence of ubiquitous U2, U5, and U6 snRNP-associated proteins, NTC-NTR components, and step II-specific factors, including SLU7, PRP17, PRKRIP1, the helicase PRP22/DHX8, and components of the exon junction complex (EJC) (Supplementary Fig. 1g). Importantly, proteins characteristic of Bact, step I or disassembly intermediates were not detected, consistent with enrichment of step II complexes. As expected, many of the identified proteins are highly divergent, showing only 10-30% sequence identity to opisthokont orthologs, and several have no identifiable orthologs (Supplementary Fig. 1h)."

Altogether, our biochemical, mass spectrometry, and cryo-EM analyses strongly support that our preparation contains predominantly, if not exclusively, step II SL trans-splicing intermediates, even though our goal was to capture complexes from B^{act} to YST states. This is now indicated more clearly the main text (see text below in red).

“Together, the RNA and protein profiles indicate that our sample is highly enriched in step II trans-spliceosomes. This was unexpected as CDC5L is a core component of the NTC normally present from activation through disassembly, suggesting that step II may represent a rate-limiting stage in trypanosomatid SL trans-splicing, with activation and step I occurring more rapidly. Notably, similar affinity strategies targeting core NTC components have also yielded late spliceosomal intermediates, including the isolation of C complexes upon CDC5L or PRP19 tagging in Chlamydomonas reinhardtii [20] and ILS complexes upon PRP19 tagging in Caenorhabditis elegans [21], consistent with system-specific spliceosomal kinetics.”*

2. Regarding the section entitled “Trypanosomatid-specific regulation of the DEAH-box helicase PRP22/DHX8”, the authors initially appear very cautious when describing model building of the putative helicase N-ter but much less so when drawing biological conclusions regarding DDX41 (in this part and in discussion). Could they elaborate on the reasons for this assignment? The authors should clarify the evidence supporting this interpretation (for example, by providing representative density for the most distinctive features of the modeled peptide) and harmonize the degree of certainty in their interpretation throughout the text.

We thank the reviewer for this comment and agree that the original text overstated the certainty of the DDX41 assignment. The attribution of the observed cryo-EM density to LtaP16.0490 rests on three independent lines of evidence: (i) a high-confidence protein-protein interaction predicted by AlphaFold2-multimer and AlphaFold3 between LtaP16.0490 and PRP22/DHX8; (ii) high peptide abundance of LtaP16.0490 in our LC–MS/MS analysis; and (iii) good agreement between the predicted interaction model and the most distinctive features of the cryo-EM density at the RecA2 lobe of PRP22. Representative cryo-EM density supporting the modeling of the N-terminal region of LtaP16.0490, together with the AlphaFold3 prediction, are now shown in Supplementary Fig. 21a–c.

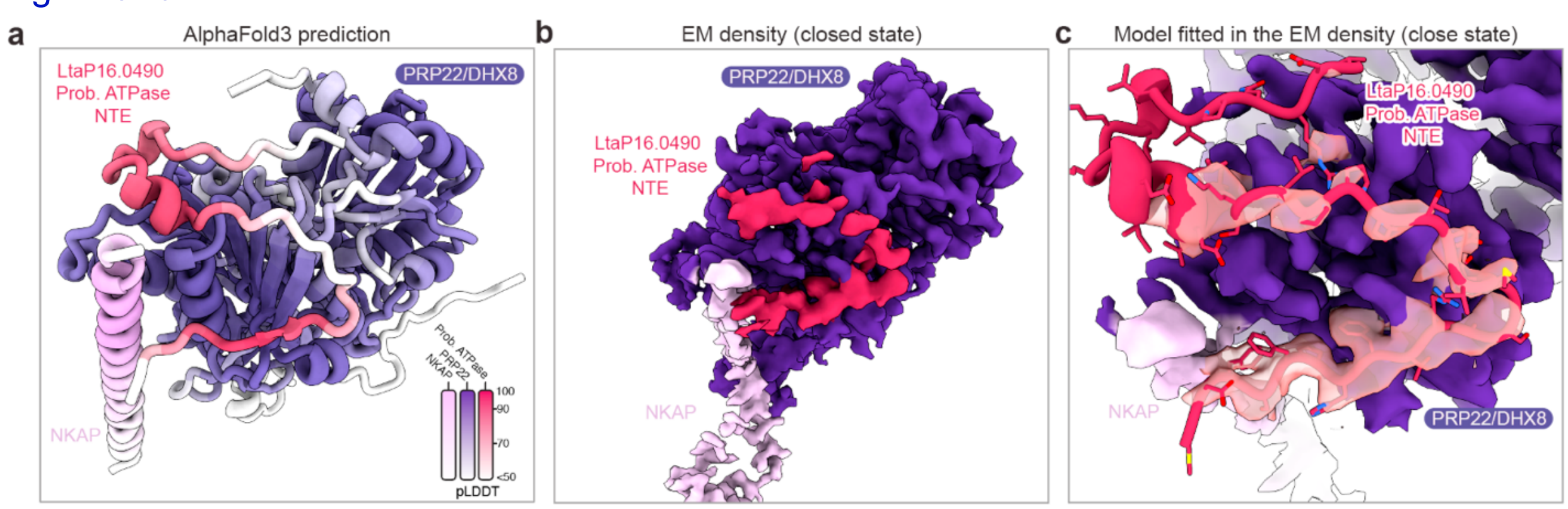

Supplementary Fig. 21. Structural and functional characterization of a PRP22-associated non-canonical ATPase (LtaP16.0490).

a *AlphaFold3 prediction of the interaction between PRP22/DHX8 and the N-terminal region of LtaP16.0490 (hereafter referred to probable ATPase). Confidence levels (pLDDT) are color-coded for each protein.* ***b*** *Cryo-EM density corresponding to the probable ATPase bound to PRP22/DHX8 in a closed state.* ***c*** *AlphaFold3 prediction fitted and adjusted into the cryo-EM*

density shown in panel b, highlighting the good fit of aromatic and aliphatic residues at the interface between the two proteins.

Our initial rationale for assigning LtaP16.0490 as a potential ortholog of DDX41 was based on two main lines of evidence. First, FoldSeek structural similarity searches classify LtaP16.0490 within the SF2 DExD/H helicase/ATPase family, with a predicted fold comprising two RecA-like domains, although multiple sequence alignments reveal degenerate Walker A and B motifs (key residues involved in ATP binding) and numerous insertion loops. Second, previous structural work in humans showed that DDX41 associates with the RecA2 lobe of DHX8/PRP22 in the C* complex[3] , in a spatial arrangement similar to what we observe between LtaP16.0490 and PRP22. However, we agree that this assignment was speculative without functional evidence, especially considering the very low percentage of sequence identity (10%).

Taking into account reviewer #2's comment, we have now experimentally assessed the ATPase activity of LtaP16.0490. We have overexpressed and purified a truncated but soluble version of LtaP16.0490 where we removed all unfolded insertion loops and measured its ATPase activity. We now show that the protein exhibits weak ATPase activity (kcat = 0.13 ± 0.03 min^{-1}), despite its degenerate ATP-binding motifs. This turnover rate is comparable to other DExD/H-box helicases, such as human DHX15 (kcat = 0.168 min^{-1})[4] , supporting the classification of LtaP16.0490 as a bona fide ATPase. We then tested by biolayer interferometry if the ATPase could bind to an immobilized poly-$(A)_{10}$ ssRNA substrate but no binding was detected at concentrations up to 10 µM of LtaP16.0490. Although this result does not demonstrate that LtaP16.0490 binds ssRNA, we cannot exclude that it requires additional cofactors to enhance its affinity for nucleic acids, as reported for DHX15 [4] . Alternatively, its physiological substrate may differ from ssRNA, or the insertions in the RecA1 domain, removed to improve expression and solubility, may contribute to substrate recognition.

Taken together, these data establish that a non-canonical SF2-like ATPase interacts with PRP22/DHX8, in an analogous architectural arrangement to what has been reported in humans, in which a second SF2 helicase docks near the RecA2 lobe of DHX8/PRP22 [3] . Whether this reflects a conserved functional coupling or convergent structural organization remains to be determined. The text and discussion have been revised throughout to reflect this level of certainty (see text below in red). We have also added the functional characterization of LtaP16.0490 in Supplementary Fig. 21f-i.

"Finally, we identified density corresponding to the N-terminal extension of an uncharacterized protein (LtaP16.0490) decorating the RecA2 lobe and stabilizing NKAP (Fig. 5e and Supplementary Fig. 21a-c). Structural similarity searches with FoldSeek revealed that this protein adopts the fold of an SF2 DExD/H helicase/ATPase, characterized by two RecA domains. Interestingly, multiple sequence alignments show that it contains numerous insertion loops and harbors degenerate Walker A (GIGKR instead of the canonical GxGKT) and Walker B (ANLE instead of the canonical DExD/H) motifs (Supplementary Fig. 21d-f). Given these deviations from canonical SF2 motifs, we sought to experimentally assess whether this protein retains ATPase activity. The purified protein revealed an intrinsic ATP turnover rate (k_{cat} = 0.13 ± 0.03 min^{-1}) comparable to the human DHX15 (k_{cat} = 0.168 min^{-1}) [55], supporting the classification of

LtaP16.0490 as a bona fide ATPase despite degenerate ATP-binding motifs (Supplementary Fig. 21g,h). Together, these data establish that a second non-canonical ATPase associates with PRP22/DHX8, although its precise functional role in SL trans-splicing remains to be determined (Supplementary Fig. 21i). Interestingly, in humans, PRP22/DHX8 has also been reported to associate with an additional SF2 RNA helicase, DDX41 [28], suggesting that docking of a second ATPase onto PRP22 may represent a conserved architectural feature across eukaryotes."

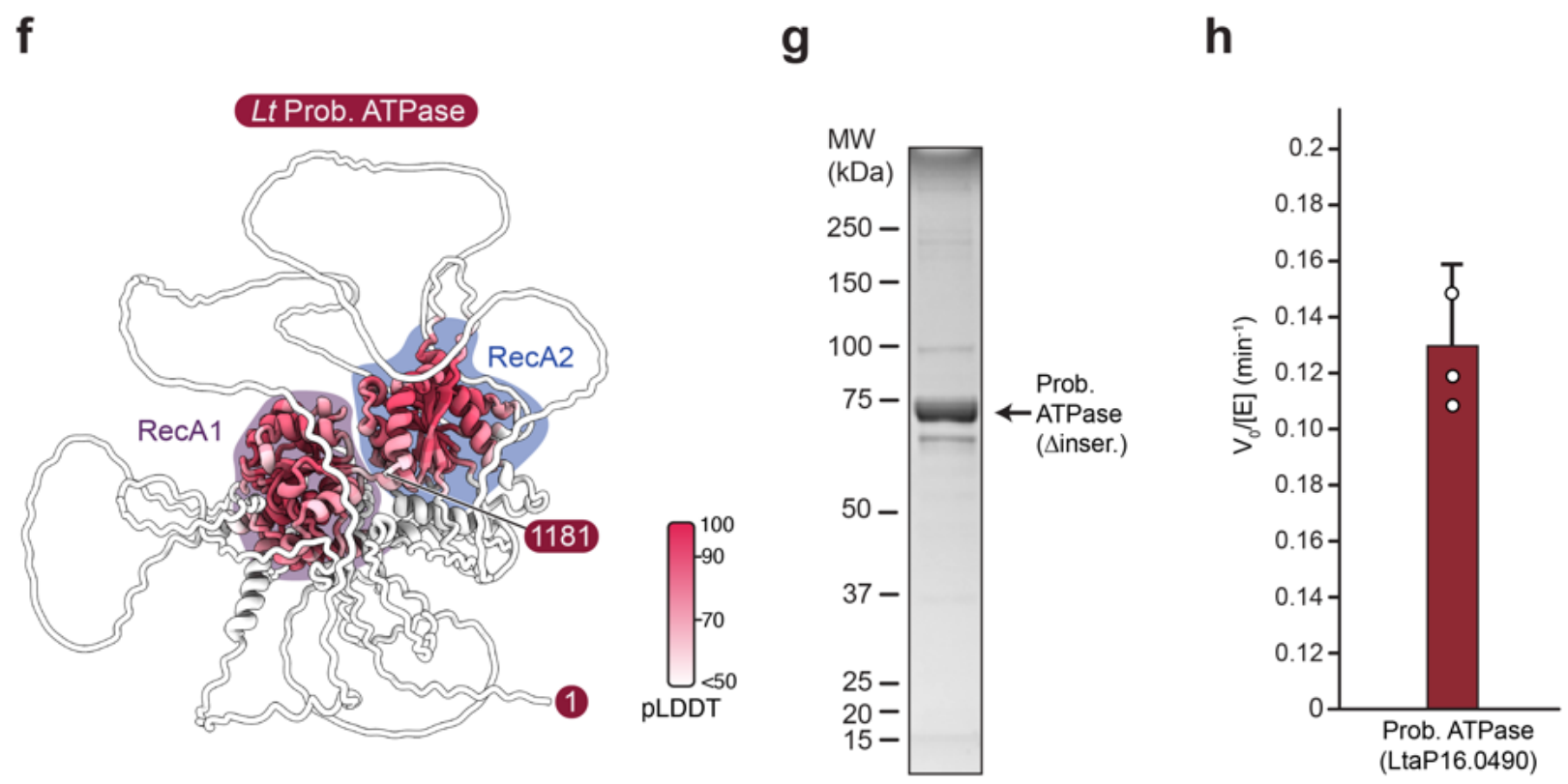

"Supplementary Fig. 21. Structural and functional characterization of a PRP22-associated non-canonical ATPase (LtaP16.0490).

f *AlphaFold3 model of the probable ATPase colored by pLDDT confidence. The two RecA domains are indicated.* ***g*** *SDS–PAGE analysis of the purified probable ATPase lacking insertion regions (Δinsertions), stained with Coomassie. The position of the truncated protein (~74 kDa) is indicated. Uncropped gel in Source Data.* ***h*** *ATPase activity of the probable ATPase, measured as initial ATP hydrolysis rates at 2 mM ATP, normalized for enzyme concentrations. The error bar represents standard deviation (SD) from triplicate measurements. Individual measurements are indicated. Source data are provided as a Source Data file."*

3. P10-5: "Proteins typically associated with intron stabilization, such as RBM22 (Cwc2/Ecm2 in yeast) and the AQR helicase, are absent, reflecting structural adaptations to SL trans-splicing. RBM22 is replaced by the Sm ring of the SL snRNP, and in the absence of AQR helicase, the U6 LSm ring occupies its position on SYF1." In yeast and human release of LSm ring of U6 occurs during Bact formation and is thought to be an important marker for spliceosome activation. This is not adequately discussed in the paper.

We thank the reviewer for raising this important point. We fully agree that the biological significance of the post-activation retention of the U6 LSm ring in trypanosomatids was not adequately discussed in the original manuscript. We have now added a dedicated discussion of this feature in the revised text (see text below in red).

“A defining trypanosomatid-specific feature is the post-activation retention of the U6 LSm ring and its stable docking onto the NTR scaffold formed by SYF1 and CRNL1, in a position normally occupied by the intron-binding helicase AQR in metazoans step II spliceosomes [27,30] (Figs. 1a and 4, Supplementary Figs. 14a). This retention is structurally enabled by two features: the absence of AQR in trypanosomatids, leaving its binding site on SYF1 vacant, and the shortened 3' end of U6 snRNA, which maintains the LSm ring in close proximity to the NTR scaffold. The LSm ring establishes tight contacts with SYF1 and PPIL1 through extensions of its LSm subunits, effectively bridging the NTC core and the NTR and reinforcing the integrity of the NTC–NTR assembly. As a consequence of this interaction network, U2/U6 helix II adopts and maintains a distinctive bent conformation (Supplementary Fig. 16a,b). Together, these interactions position the LSm ring as an integral structural component of the activated trans-spliceosome rather than a transient pre-activation factor, representing a major divergence from canonical splicing systems. Indeed, in yeast and human spliceosomes, the U6 LSm ring is released during activation between the pre-$B^{act\text{-}I}$ and pre-$B^{act\text{-}II}$ stages [50] , and its dissociation is considered a hallmark of catalytic activation [51,52]. The persistence of the LSm ring raises mechanistic questions. In opisthokonts, release of the LSm ring exposes the 3' end of U6 for engagement by DHX15 during spliceosome disassembly [23]. In trypanosomatids, by contrast, the 3' end remains shielded by the LSm ring in step II complexes. Whether the LSm ring is displaced at a later stage or whether disassembly proceeds via a distinct mechanism remains unknown. Because the molecular choreography of activation and disassembly has not yet been structurally characterized in trypanosomatids, it is unclear whether LSm retention represents a compensatory adaptation to the absence of AQR or reflects a broader divergence in spliceosome dynamics.”

4. P10-I10 : “Strikingly, the branch helix, formed by base pairing between the pre-mRNA and U2 snRNA, is not visible, and nucleotides surrounding the branchpoint remain flexible, consistent with the absence of a conserved branchpoint motif in trypanosomatids and reinforcing the view that branchpoint selection is instead primarily guided by the downstream polypyrimidine tract” The link between the flexible nature of the branch and the low degree of conservation of the contacts is questionable since a similar argument could be brought forward in human where the branch sequence is very variable but well visible in the cryo-EM structures.

   We thank the reviewer for this comment. Indeed, the branch helix is visible in cryo-EM structures of human step II spliceosomes, even though the branch sequences are highly variable, whereas it is not resolved in our trypanosomatid structures. We interpret this difference as resulting from two factors. First, trypanosomatid pre-mRNA lacks a branchpoint recognition motif, unlike in humans where a motif is present despite sequence variability. Second, the heterogeneity of endogenous pre-mRNA in our sample likely further prevents formation of a stable branch helix detectable by cryo-EM. We have revised the main text accordingly (see text below in red).

   “Interestingly, nucleotides immediately surrounding the branchpoint adenosine remain flexible and are not stabilized by base pairing with U2 snRNA (Supplementary Figs. 10, 11b, 13). The lack of density at the branchpoint likely reflects the absence of a conserved branchpoint recognition motif in both the U2 snRNA and the pre-mRNA involved in trypanosomatid SL trans-splicing [11,35],

which may hinder formation of stable interactions. Consistently, the branch helix upstream of the branchpoint, normally formed by base pairing between the pre-mRNA and U2 snRNA, is not resolved in our structures. This apparent flexibility and lack of defined density could be further influenced by the heterogeneity of endogenous pre-mRNA substrates in our sample. By contrast, in human spliceosomes, even highly variable pre-mRNA branch sequences can still engage with the conserved U2 branchpoint recognition motif, producing a defined branch helix despite sequence degeneracy [23,28]. Together, these observations are consistent with the view that branchpoint selection in trypanosomatids is primarily guided by the downstream polypyrimidine tract during early stages of trans-spliceosome assembly, as also observed in metazoans. Still, the underlying molecular mechanism remains incompletely understood in trypanosomatids, as the precise roles of U2AF35, U2AF65, and SF1 in branchpoint recognition and U2 snRNP recruitment are still not fully defined [36,37] ."

5. P10" with unique RNA-protein interactions that compensate for the absence of canonical introns and a branchpoint recognition motif, while preserving the fundamental chemistry of exon ligation." Is probably true but the text fails to clearly convey the idea by giving concrete examples.

We thank the reviewer for this remark. To clarify this point, we have revised the text to include specific examples illustrating how the interaction network is reorganized in the absence of canonical intron-stabilizing factors (see text below in red).

"The absence of these intron-stabilizing factors is also accompanied by extensive rewiring of the surrounding protein network. For example, PPIL1 in trypanosomatids has evolved an extended C-terminal tail that engages the WD40 domain of PRP19 atop the PRP19 tetramer, compensating for the simplified interface caused by the loss of RBM22. The N-terminal tails of PRP17 and SNW1 adopt new contacts with PPIL1, creating a distinct interface that differs markedly from human and yeast spliceosomes. At the NTC core, interactions between SNW1, CDC5L, and PRP17 are reorganized: in humans, RBM22 stabilizes an extensive network between these proteins, whereas in trypanosomatids, alternative contacts form directly among SNW1, CDC5L, and PRP17 to maintain stability of the catalytic site and the U6–SL outron helix (Supplementary Fig. 14b). Together, these examples illustrate how trypanosomatids have reconfigured protein–protein interactions to stabilize RNA duplexes forming the catalytic core in the absence of canonical intron-associated factors, highlighting a structural architecture adapted for SL trans-splicing while preserving the fundamental chemistry of exon ligation."

6. The section describing the EJC is somewhat confusing and needs clarification/reinterpretation. On p.8, l.10, the authors state: "A key distinction at the protein level between the trans-C and trans-P complexes is the release of the EJC and the destabilization of the step II factors CACTIN, SLU7, and PRP18 following exon ligation, as similarly observed in the yeast P complex." The term "protein level" is misleading, as it suggests biochemical evidence, whereas the conclusions presented here are derived exclusively from structural observations.

We thank the reviewer for the insightful comments and for encouraging us to clarify our interpretation of the EJC state during the transition from the trans-C* to the trans-P complex. We agree that the wording "at the protein level" could be misleading, as the results presented in this section derive exclusively from cryo-EM structural observations. We therefore revised the sentence as follows (see text below in red):

"Among step II factors, CACTIN, SLU7, and PRP18 are present in the trans-C complex and become destabilized in the post-catalytic trans-P complex, as observed in the yeast P complex [29] (Supplementary Fig. 9)*

…

Prior to ligation, the SL exon is associated with an extended exon junction complex (EJC). In the post-catalytic state, density corresponding to the EJC is absent, indicating that the EJC is no longer stably associated with the trans-spliceosome through RNA or protein-mediated contacts following SL exon ligation (Fig. 1a,b and Supplementary Fig. 2)."

More importantly, it remains unclear how the authors interpret the fate of the EJC between the C* and P states. If the EJC undergoes "detachment" (p11 and discussion) during this transition, what precisely is meant by it remaining "tethered" (Supplementary Fig. 7B; Discussion, p.16)? The EJC is also depicted in Fig. 2B as binding the 5′ exon. Finally, the statement in the Discussion that "Through its NTE, PRP18 transmits these events to the EJC, which detaches but remains tethered to the trans-spliceosome via the SL exon" is particularly intriguing and difficult to reconcile with the structural data. If I understood correctly, the authors appear to propose that the EJC remains clamped onto the 5′ exon after dissociating from Cwc22. However, inspection of the cryo-EM densities argues against this interpretation: there is no residual density in the region where the EJC would be expected in the P complex, and given the length of the RNA protruding from the spliceosome in this region, an EJC still bound to the RNA should remain at least partially ordered and thus partially visible. An alternative explanation would be that the EJC dissociates entirely, which would be a surprising and potentially important finding. I therefore encourage the authors to clarify their interpretation of the EJC state between the C* and P complexes and to reconcile it more explicitly with the observed densities. In addition, further discussion of prior work on DEAD-box helicase-mediated RNA clamping, and how nucleotide state influences RNA engagement and release, would greatly strengthen this section and help contextualize the proposed mechanism in line with their observation that ADP and not ATP would be bound to eIF4AIII...

We thank the reviewer for carefully examining this section and for raising important points regarding the interpretation of the EJC state between the trans-C* and trans-P complexes. We agree that the previous wording could lead to confusion regarding the fate of the EJC following SL exon ligation.

In the original version of the manuscript, we suggested that the EJC might detach from its docking platform on the trans-spliceosome while remaining tethered to the SL exon through the RNA clamp formed by eIF4A3. However, as correctly pointed out by the reviewer, this interpretation is not supported by our cryo-EM data. In the trans-P complex we do not observe any residual density corresponding to the EJC in the region where it would be expected if it remained associated with the SL exon.

We therefore agree with the reviewer that the structural data are more consistent with complete dissociation of the EJC from the SL exon following exon ligation. We have revised the manuscript accordingly and removed the interpretation that the EJC remains “tethered” to the trans-spliceosome. The text now clearly states that density corresponding to the EJC is absent in the trans-P complex, indicating that the EJC is no longer associated with the trans-spliceosome following SL exon ligation (Results and Discussion sections, Fig. 1 and Fig. 6).

Following the reviewer’s suggestion, we also expanded the mechanistic interpretation of the EJC nucleotide state. In particular, we now emphasize that our structure reveals ADP bound to eIF4A3 in the trans-C* complex, in contrast to previously reported spliceosomal structures in which ATP was modeled. Consistent with prior structural and biochemical work showing that the MAGOH–RBM8A heterodimer stabilizes a closed helicase conformation after ATP hydrolysis [5] , our data suggest that the EJC is captured in our structure in a post-hydrolysis ADP-bound state while bound to the SL exon. Because ATP hydrolysis is known to reduce RNA affinity in DEAD-box helicases [6,7] , this state may prime the complex for dissociation once spliceosomal interactions that stabilize the EJC are weakened.

Finally, we now discuss more explicitly that it remains unclear whether the ADP-bound state observed in our structure reflects a general feature of EJC dynamics during splicing or instead represents a species-specific adaptation associated with trans-splicing in trypanosomatids, given the limited high-resolution structural information currently available for EJCs within spliceosomes from other eukaryotes. The Results and Discussion sections have been revised accordingly to clarify these points (see text below in red).

Results

“Strikingly, as opposed to cryo-EM structures of cis-C/C/P complexes where ATP-bound eIF4A3 is modeled, we unambiguously identified ADP bound to eIF4A3 (Fig. 3a). Structural and biochemical studies have shown that eIF4A3 clamps RNA in a closed conformation upon ATP binding, a state further stabilized by the MAGOH–RBM8A heterodimer, which prevents nucleotide exchange and reopening of the helicase core after ATP hydrolysis [44–46]. Consistent with this mechanism of post-hydrolysis inhibition, our data suggest that ATP hydrolysis occurs within the assembled EJC, generating an ADP-bound eIF4A3 that remains in a closed conformation, clamping the EJC onto the SL exon in the trans-C* complex. Because ATP hydrolysis reduces RNA affinity in DEAD-box helicases[47,48] , this post-hydrolysis state may also prime the complex for dissociation from the SL RNA once EJC and spliceosomal protein-protein interactions are destabilized upon step II factors release. In agreement with this model, density corresponding to the EJC is absent in the post-catalytic trans-P complex, indicating that the EJC is no longer associated with the trans-spliceosome following SL exon ligation. Whether this ADP-bound state reflects a general feature of EJC dynamics during splicing or instead represents a species-specific adaptation associated with trans-splicing in trypanosomatids or a trans-C* substate after ATP hydrolysis remains unclear, particularly given the limited high-resolution structural information available for EJCs in spliceosomes from other eukaryotes [23,25,31,49].”*

Discussion

"Our structures further reveal that a complete exon junction complex (EJC) assembles on the conserved SL exon during trans-splicing. Unlike metazoans, where EJCs are deposited on multiple spliced exons and serve as critical markers for NMD, trypanosomatids are largely intronless, suggesting that the EJC is unlikely to participate in NMD [39]. Instead, the conserved SL exon provides a single EJC-binding platform that assembles transiently during trans-splicing. The release of the EJC following SL exon ligation suggests that, unlike in metazoans where EJCs remain associated with mRNAs until the pioneer round of translation and act as persistent markers for mRNP transport and downstream regulation[57] , EJC binding in trypanosomatids is likely transient. Rather than serving as a stable mRNA landmark, the EJC may instead stabilize the SL exon prior to ligation and signal completion of the splicing reaction upon its dissociation. This behavior therefore appears distinct from the canonical metazoan paradigm and may reflect species-specific adaptations associated with the unique organization of trypanosomatid gene expression. In this context, transient EJC assembly could provide a checkpoint that ensures proper assembly and catalysis of the trans-spliceosome before release of the mature SL-capped mRNA. Because the mechanisms governing mRNA export in trypanosomatids remain incompletely understood, it is currently unclear whether the EJC contributes to downstream mRNP maturation in these organisms [58,59]."

P11 "We also identified the extended N-terminus of PRP18 forming direct contacts with the CWC22 NTD, thereby coupling SL exon binding by the EJC with SL exon ligation sensing through the PRP18 insertion loop into the catalytic site". Regardless of the comment above, this is an appealing hypothesis. Could the author justify better their decision to attribute this density to Prp18, could they show some density supporting this attribution?

We thank the reviewer for this comment and for the opportunity to clarify the assignment of this density. The attribution of this density to the N-terminal extension (NTE) of PRP18 is supported by converging structural evidence. First, AlphaFold-Multimer predictions from our PPI screen, further supported by AlphaFold3 predictions, identify a high-confidence interaction between a helical segment of the PRP18 NTE (residues 76-99) and the CWC22 N-terminal domain (NTD). Second, this predicted N-terminal helix fits well into the cryo-EM density at the surface of the CWC22 NTD. In addition, the distal portion of the NTE (residues 1–15) can be confidently modeled into adjacent density, where well-resolved hydrophobic features at the protein-protein interface further support the assignment. Interestingly, neither AlphaFold-Multimer nor AlphaFold3 predicts an interaction between the distal segment of the PRP18 NTE (residues 1–15) and the CWC22 NTD. This discrepancy may be explained by the closed conformation of the CWC22 NTD in the predictions, in contrast to the more open conformation observed in the cryo-EM structure, which can accommodate this distal region. To further support this assignment, we have now included the AlphaFold3 prediction alongside representative cryo-EM density highlighting the key structural features of this interface (Supplementary Fig. 15d,e).

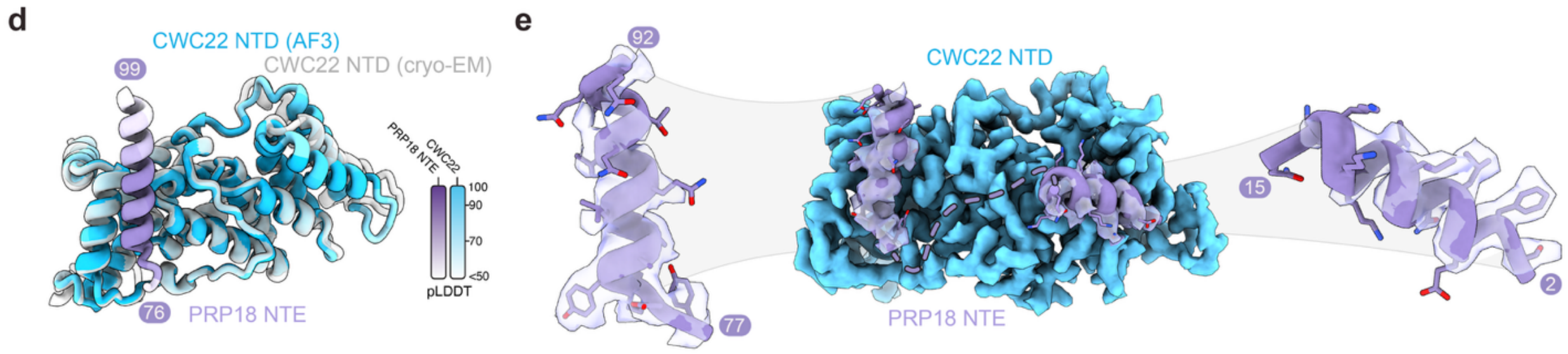

*"**Supplementary Fig. 15. d** AlphaFold3 prediction of the interaction between the PRP18 N-terminal extension (NTE) and the CWC22 N-terminal domain (NTD). Confidence levels (pLDDT) are color-coded for each protein. Notably, AF3 does not predict an interaction between the distal PRP18 NTE (residues 1–15) and the CWC22 NTD. This may be explained by the closed conformation of the CWC22 NTD in the prediction, in contrast to the more open conformation observed in the cryo-EM structure, which can accommodate the distal PRP18 NTE. **e** Cryo-EM density of the PRP18 NTE in complex with CWC22, with insets highlighting the well resolved hydrophobic residues at the protein-protein interface."*

7. Along the text, which is generally rather condensed, some statements are repeated several times. For example, p10 l5 "RBM22 is replaced by the Sm ring of the SL snRNP, and in the absence of AQR helicase, the U6 LSm ring occupies its position on SYF1" and p7 l20 "It protrudes outward, positioned atop BUD31, occupying the site normally held by intron-bound RBM22 in human spliceosomes or Cwc2/Ecm2 in yeast spliceosomes" and p7 l10 "U6 snRNP is composed of U6 snRNA and a heptameric LSm ring (LSm2–8), which is clearly resolved and docked onto the Nineteen Complex (NTC), occupying the site typically held by the intron-stabilizing AQR helicase in human spliceosomes" and p13 l5 "the LSm ring docks on SYF1 in a position normally occupied by the intron-binding helicase AQR in metazoans, creating a unique conformation of U2/U6 helix II".

We thank the reviewer for this helpful comment. We agree that several statements were repeated across the manuscript. We have now carefully revised the main text, in particular the section describing the overall architecture of the step II complexes, to streamline the presentation. The revised version explicitly frames the description in terms of features conserved with cis-splicing, trans-splicing-specific adaptations, and lineage-specific innovations in trypanosomatids, and removes redundant descriptions of these structural features. As a result, each feature is now introduced once in the appropriate context, which should improve clarity and reduce repetition throughout the manuscript (see text below in red).

*"**Overall structure of step II trypanosomatid trans-spliceosomes***

The overall architecture of the step II trypanosomatid trans-spliceosomes closely resembles that of canonical step II cis-spliceosomes [25–32] (Fig. 1, and Supplementary Figs. 7,8). Conserved features include the assembly of U2, U5, and U6 snRNPs into the RNA catalytic core, which is supported by the NTC–NTR network that stabilizes the activated conformation and facilitates the transesterification reactions. Among step II factors, CACTIN, SLU7, and PRP18 are present in

the trans-C complex and become destabilized in the post-catalytic trans-P complex, as observed in the yeast P complex [29] (Supplementary Fig. 9). The DEAH-box helicase PRP22/DHX8 is poised for mRNA release following SL exon ligation.*

Beyond this conserved core, two divergent aspects distinguish the step II trypanosomatid trans-spliceosome: adaptations specific to SL trans-splicing and lineage-specific features that are present across cis- and trans-splicing in trypanosomatids (Fig. 1b). Unique to SL trans-splicing is the SL snRNP, which functions both as an architectural snRNP and as a substrate, providing the SL exon for the step II reaction. Prior to ligation, the SL exon is associated with an extended exon junction complex (EJC). In the post-catalytic state, density corresponding to the EJC is absent, indicating that the EJC is no longer stably associated with the trans-spliceosome through RNA or protein-mediated contacts following SL exon ligation (Fig. 1a and Supplementary Fig. 3). Trypanosomatid-specific adaptations further shape the step II complexes, including retention of the U6 LSm ring, incorporation of numerous lineage-specific proteins such as Sm16.5K, Sm15K, PRC3, PRC5, SDE2-like and RBP1, and structural expansions and remodeling of conserved modules, including PRP18 NTE, CRNL1 CTE, CACTIN NTD, PRP22 NTD, CWC21 CTE, and PPIL1 CTE domains (Fig. 1b). These distinctive SL trans-splicing and trypanosomatid-specific features are described in more detail in the following sections. For an in-depth view of these states at the atomic coordinate level, PyMOL sessions and movies are available (Supplementary Data 2, 3, and Supplementary Movies 1, 2).”

Reviewer #2 (Remarks to the Author)

Trypanosomatids such as Trypanosomes and Leishmania employ a unique trans-splicing mechanism. Their genes were transcribed in a single polycistronic pre-mRNA and splicing of each gene occurs in trans with a splice-leader (SL) RNA. As a result, each resulting pre-mRNA has the same splice-leader exon at their 5' end.

In this paper, the authors provided the first insight into this SL-trans-splicing process. They tagged a late-stage splicing subunit in Leishmania, purified the complex, characterised its composition and potentially protein-protein interactions using AlphaFold. They then determined the structures of the purified complex and resolved the structures of the C* (prior to second step of splicing) and P (post splicing). Through focused classification/refinement they obtained higher resolution maps at different flexible parts of the structures, yielding a composite maps. The structures revealed a conserved core that is highly similar to the canonical cis-splicing complexes. They then discussed several features of the structures: 1) SL RNA recognition by the Exon Junction Complex (EJC), which binds at exon-intron junctions and marks the RNA for non-sense mediated decay (NMD). 2) Trypanosome-specific proteins or extensions that have been adapted to the shortening of the snRNAs. 3) the RNA helicase Prp22, which release the SL capped pre-mRNA from the trans-spliceosome.

Generally, the strength of the paper is that it provides the first structural insights into SL-trans-splicing. It will be of broad interest to people in both the splicing field and parasite biology. The cryo-EM analysis is done nicely. On the other hand, there are also some weaknesses. There is no functional data to support any key interactions observed and to elucidate the roles of some of trypanosome-specific factors. As a result, the paper contains mostly detailed descriptions but it lacks deep analyses of how their observations relate to the unique mechanism of trans-splicing. Some of the suggested functional implications of the structural observations are purely speculative. Therefore, while I think it is a nice piece of work, addition of functional work will be critical to validate some of the structural observation and elevate the impact of the paper.

We thank the reviewer for the careful evaluation of our manuscript and for the positive assessment of its novelty and broad interest. We also acknowledge the concern regarding the absence of functional validation. We fully agree that functional characterization is essential to further dissect the mechanistic implications of structural observations, and we always aim to complement our structural work with biochemical analyses whenever technically feasible. In this context, already prior to the first submission of this manuscript, we made extensive efforts to biochemically characterize *L. tarentolae* PRP22, including variants targeting its N-terminal linker region, which we hypothesize to be a key regulatory element. However, despite substantial efforts, these experiments proved technically unfeasible due to persistent solubility and stability issues (see point 7 below). As we believed that the detailed structural insights presented here already represented a substantial advance for the field of RNA splicing and would enable targeted functional investigations moving forward, we proceeded with submission of our manuscript.

Prompted by the reviewer's comments, we renewed our efforts during the revision to obtain functional insight into selected aspects raised by the reviewer. We also have revised the manuscript to further clarify the speculative nature of certain interpretations and to clearly distinguish features that are unique to SL trans-splicing from those that are shared with canonical cis-splicing systems in opisthokonts, as well as trypanosomatid-specific adaptations. Finally, we have addressed all of the reviewer's specific comments below.

1. Page 8, line 7. The authors identified a protein that they called "putative" RNA helicase and suggested that it could be a functional analog of human DDX41. With only 10% sequence identity, they should provide evidence including helicase assay and ATPase assay to support this helicase assignment. Similarly, for the DDX41 analog assignment, functional data should be required.

We thank the reviewer for this comment. Our initial assignment of LtaP16.0490 as a putative RNA helicase was based on converging evidence from sequence and structural analyses. FoldSeek searches and AlphaFold structural predictions consistently classify it within the SF2 helicase/ATPase family, revealing a fold composed of two RecA-like domains. Subsequent multiple sequence alignments revealed the presence of degenerate Walker A and B motifs (key residues involved in ATP binding) and numerous insertion loops. Prompted by the reviewer's suggestion, we were able to overexpress and purify this challenging protein and experimentally assessed its biochemical properties. First, we show that the protein exhibits weak ATPase activity (k_{cat} = 0.13 ± 0.03 min^{-1}), despite the degenerate ATP binding motifs. This ATP turnover activity is similar to other DExD/H-box helicases, such as human DHX15 (k_{cat} = 0.168 min^{-1}) [4] , supporting the classification of LtaP16.0490 as a bona fide ATPase (see figure below). We next assessed its ability to bind an ssRNA substrate, but no interaction with a chip-immobilized poly-$(A)_{10}$ ssRNA was detected by bio-layer interferometry, even at protein concentrations up to 10 µM (see figure below). Although this result could not confirm that LtaP16.0490 binds ssRNA, we cannot exclude that it requires additional cofactors to enhance its affinity for nucleic acids, as reported for DHX15 [4] , or that its physiological substrate differs from ssRNA.

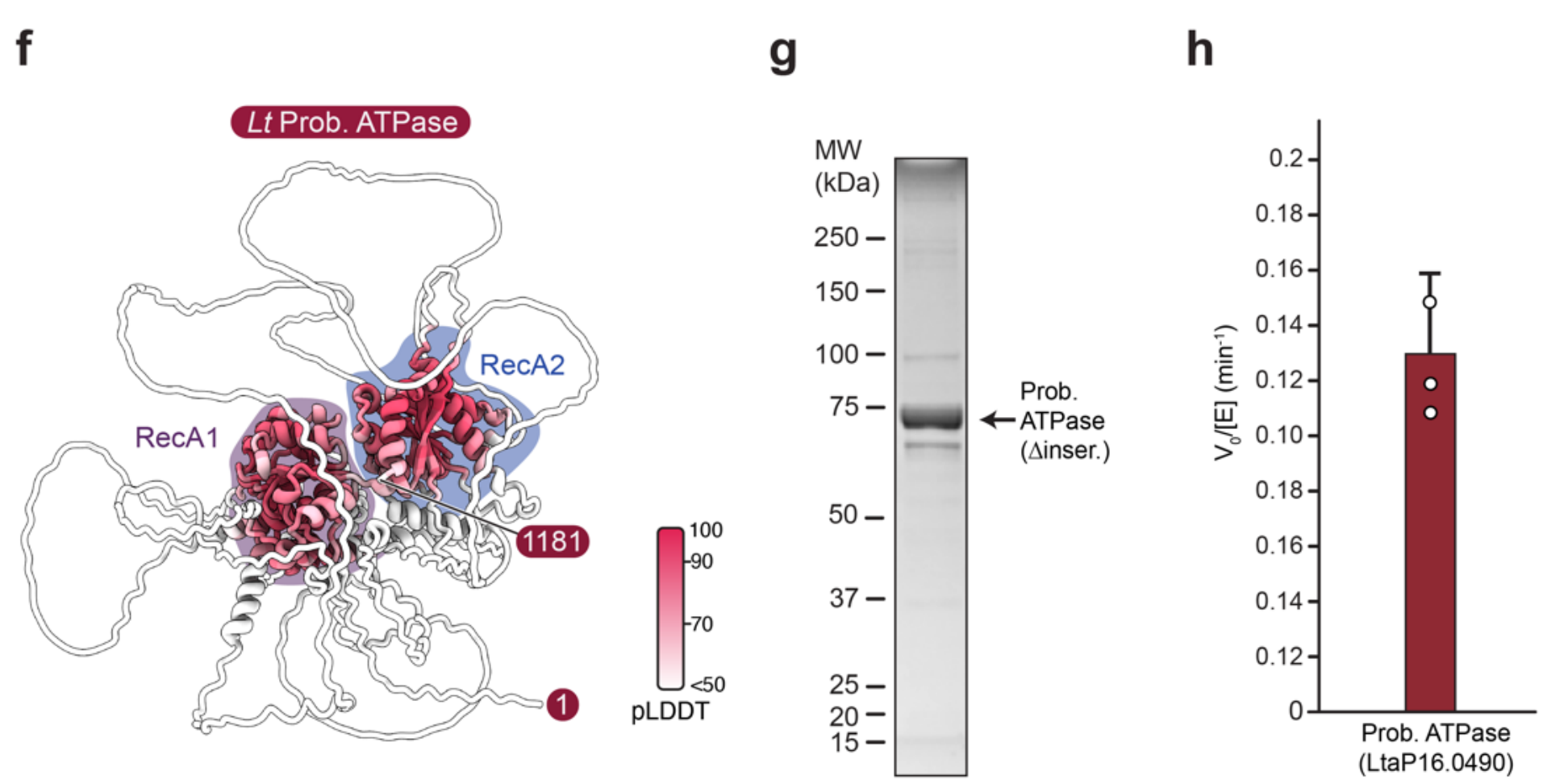

*“**Supplementary Fig. 21. Structural and functional characterization of a PRP22-associated non-canonical ATPase (LtaP16.0490).***

***f** AlphaFold3 model of the probable ATPase colored by pLDDT confidence. The two RecA domains are indicated. **g** SDS–PAGE analysis of the purified probable ATPase lacking insertion regions (Δinsertions), stained with Coomassie. The position of the truncated protein (~74 kDa) is indicated. Uncropped gel in Source Data. **h** ATPase activity of the probable ATPase, measured as initial ATP hydrolysis rates at 2 mM ATP, normalized for enzyme concentrations. The error bar represents standard deviation (SD) from triplicate measurements. Individual measurements are indicated. Source data are provided as a Source Data file.”*

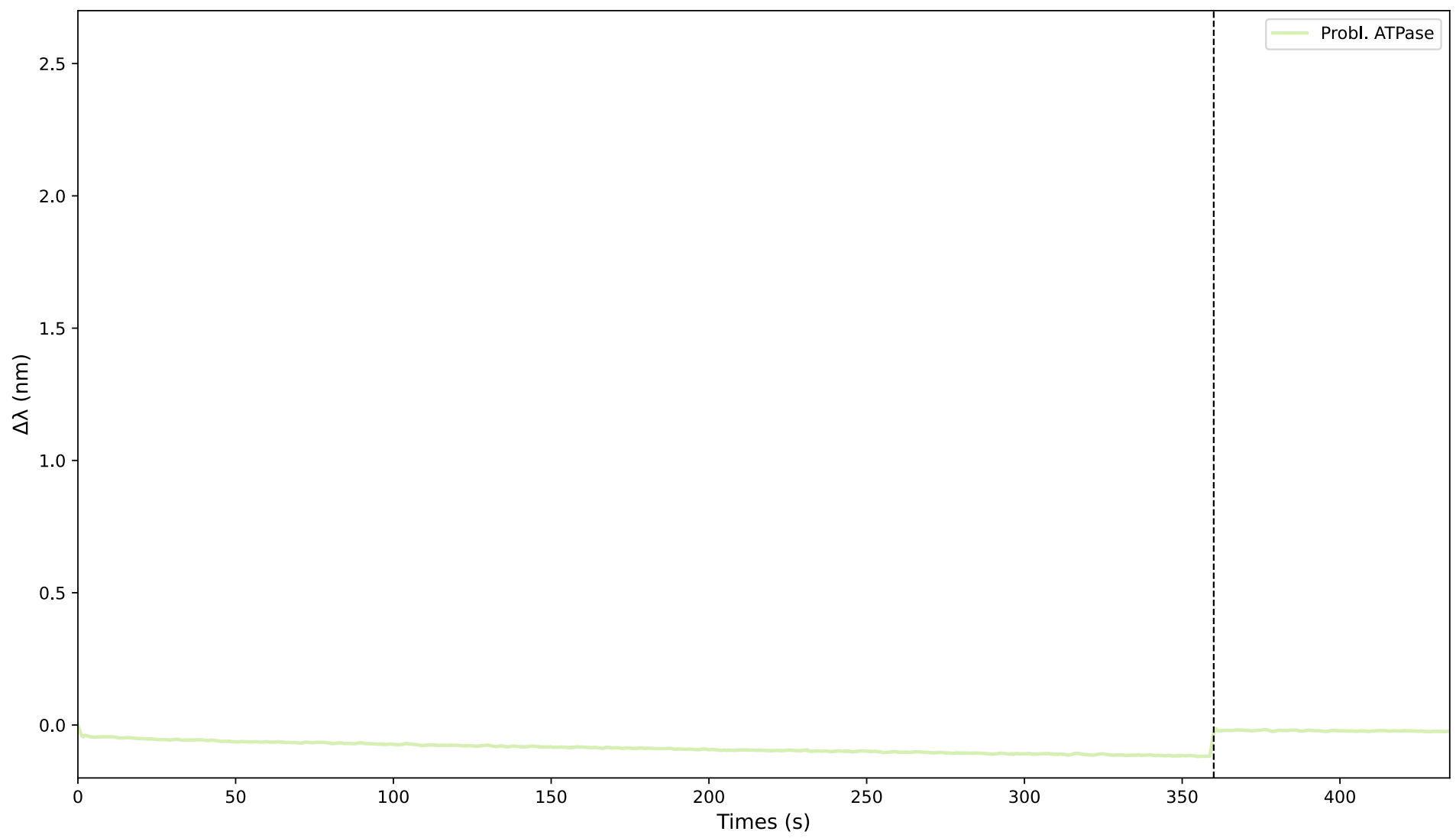

Biotinylated RNA was immobilized on streptavidin (SA)-coated biosensors. Briefly, SA biosensors were hydrated in buffer (50 mM HEPES pH 7.8, 100 mM NaCl, 20 mM $MgCl_2$, 1 mM DTT, 0.01% Tween-20, 0.1% BSA, 2 mM ATP) for 10 min prior to the measurements. The sensors were then loaded with biotinylated RNA (5 µg/mL) for 300 s. Remaining streptavidin binding sites were subsequently quenched by incubating the biosensors in biocytin (100 µg/mL) for 300 s. After a baseline step in buffer (180 s), the biosensors were dipped into solutions containing 10 µM LtaP16.0490 (Prob. ATPase), for 360 s to monitor the association phase. The dissociation phase was then monitored by transferring the biosensors into buffer for up to 900 s. As no interaction was detected, the experiment was stopped after 80 s of dissociation. The data shown in the figure represent the processed association and dissociation curves, where the signal obtained with biosensors lacking immobilized RNA was subtracted from the signals measured with RNA-loaded biosensors. As this result does not contribute to the characterization of LtaP16.0490, we have decided not to include it in the manuscript.

We believe that a full biochemical and mechanistic characterization of this protein would be very interesting but would require a dedicated study beyond the scope of the present manuscript. We therefore refer to LtaP16.0490 as a probable ATPase in the manuscript, as its precise function and substrate specificity remain to be established.

Regarding the potential analogy to human DDX41, our initial hypothesis was motivated by the observation that in the human C* complex, DDX41 has been proposed to localize near the RecA2 domain of DHX8 [3] . The trypanosomatid complex reveals a similar architectural arrangement in which a second ATPase protein is tethered to PRP22. However, we agree that this assignment was speculative without functional evidence, especially considering the very low percentage of sequence identity (10%). In the revised manuscript, we have clarified the wording to emphasize that this observation suggests a potentially conserved architectural feature rather than a demonstrated functional analogy (see text below in red).

"Finally, we identified density corresponding to the N-terminal extension of an uncharacterized protein (LtaP16.0490) decorating the RecA2 lobe and stabilizing NKAP (Fig. 5e and Supplementary Fig. 21a-c). Structural similarity searches with FoldSeek revealed that this protein adopts the fold of an SF2 DExD/H helicase/ATPase, characterized by two RecA domains. Interestingly, multiple sequence alignments show that it contains numerous insertion loops and harbors degenerate Walker A (GIGKR instead of the canonical GxGKT) and Walker B (ANLE instead of the canonical DExD/H) motifs (Supplementary Fig. 21d-f). Given these deviations from canonical SF2 motifs, we sought to experimentally assess whether this protein retains ATPase activity. The purified protein revealed an intrinsic ATP turnover rate (k_{cat} = 0.13 ± 0.03 min^{-1}) comparable to the human DHX15 (k_{cat} = 0.168 min^{-1}) [55], supporting the classification of LtaP16.0490 as a bona fide ATPase despite degenerate ATP-binding motifs (Supplementary Fig. 21g,h). Together, these data establish that a second non-canonical ATPase associates with PRP22/DHX8, although its precise functional role in SL trans-splicing remains to be determined (Supplementary Fig. 21i). Interestingly, in humans, PRP22/DHX8 has also been reported to associate with an additional SF2 RNA helicase, DDX41 [28], suggesting that docking of a second ATPase onto PRP22 may represent a conserved architectural feature across eukaryotes."

2. Page 9 Line 20: for the following claim "G39 is likely stochastically flipping in and out until the second trans-esterification reaction occurs. This dynamic conformation is enabled by the absence of a FAM32 ortholog in trypanosomatids", the authors should provide more explanations and make a figure comparing with other spliceosome structures containing FAM32.

We thank the reviewer for this comment and apologize for the lack of clarity. This comparison was already included in Supplementary Fig. 12, where we directly contrast the conformation of the terminal SL exon nucleotide (G39) with corresponding states of human spliceosomes that contain FAM32. As shown in the figure, in opisthokonts the terminal exon nucleotide (G-1) is stabilized in a reaction-ready conformation through interactions with FAM32 and U5 snRNA nucleotide U17. In contrast, in the *Leishmania* trans-C* complex, no FAM32 ortholog is present, and G39 lacks stabilizing interactions with U17, resulting in a flipped-out conformation. We have also revised the main text to make this point clearer (see text below in red).

"In the trans-C complex, before the SL exon is ligated to the pre-mRNA, the terminal nucleotide of the SL exon (G39) adopts a flipped-out conformation, positioned away from the 5' UTR and lacking stabilizing interactions with U17 of the U5 snRNA or coordination with the M1 catalytic*

metal ion. In contrast to the human C complex where the equivalent exon nucleotide is stabilized by FAM32 and U5 snRNA in a reaction-ready orientation, trypanosomatids lack a FAM32 ortholog. Based on this comparative structural analysis, we propose that, in the absence of these stabilizing interactions, G39 samples alternative conformations in the trans-C* complex until productive alignment for the second trans-esterification reaction is achieved. In the trans-P complex G39 adopts an inserted conformation and is ligated to the 5' UTR of the pre-mRNA. (Fig. 2c, and Supplementary Fig. 12).”*

3. Page 12, lined 11-14. The authors made an interesting observation about the nucleotide binding state of the eIF4A3 and suggested that previous structures may have mis-interpreted it. How can they rule out that species-specific difference could account for the difference in the nucleotide state? Or the sub-state of the spliceosome in their structure may be different from those reported and could account for such difference. Additional evidence to back their suggestion should be provided to rule out these possibilities.

We thank the reviewer for this important comment and for highlighting alternative explanations for the observed nucleotide state of eIF4A3. We agree that, based on our data alone, we cannot formally exclude the possibility that the ADP-bound state observed in our structure reflects either a species-specific feature of trypanosomatid splicing or a distinct conformational substate of the trans-spliceosome compared to previously reported cis-splicing complexes. We have therefore revised the manuscript to more clearly acknowledge these possibilities and to avoid overinterpretation. In light of prior biochemical and structural studies showing that human eIF4A3 adopt a closed RNA-clamped conformation following ATP hydrolysis, stabilized by MAGOH–RBM8A [5,8] , we propose that this state may represent a post-hydrolysis intermediate that primes the EJC for dissociation. However, we explicitly state that whether this reflects a general feature of spliceosomal EJC dynamics or a system-specific adaptation remains an open question. We have revised the corresponding sections in the Results section to clarify these points (see text below in red).

“Strikingly, as opposed to cryo-EM structures of cis-C/C/P complexes where ATP-bound eIF4A3 is modeled, we unambiguously identified ADP bound to eIF4A3 (Fig. 3a). Structural and biochemical studies have shown that eIF4A3 clamps RNA in a closed conformation upon ATP binding, which is stabilized by the MAGOH–RBM8A heterodimer and prevents reopening of the helicase core following ATP hydrolysis [44–46]. Consistent with this mechanism of post-hydrolysis inhibition, our data suggest that ATP hydrolysis has occurred within the assembled EJC, generating an ADP-bound state that maintains a closed helicase conformation and stabilizes docking of the complex on the SL exon in the trans-C* complex. Because ATP hydrolysis reduces RNA affinity in DEAD-box helicases[47,48] , this post-hydrolysis state may also prime the complex for dissociation from the SL RNA once spliceosomal protein-protein interactions are destabilized upon step II factors release. In agreement with this model, density corresponding to the EJC is no longer observed in the post-catalytic trans-P complex, indicating that the EJC is no longer associated with the trans-spliceosome following SL exon ligation. Whether this ADP-bound state reflects a general feature of EJC dynamics during splicing or instead represents a species-specific*

adaptation associated with trans-splicing in trypanosomatids remains unclear, particularly given the limited high-resolution structural information available for EJCs in spliceosomes from other eukaryotes [23,25,31,49].»

4. They also proposed a “locking and docking” mechanism for eIF4A3 without additional evidence. Either experiments are performed to show this or they should explain why experiments are not feasible.

   We thank the reviewer for this comment. Following this and related comments, we have removed the “locking and docking” model from the revised manuscript. This interpretation was based on our initial assumption that the EJC remained associated with the SL exon following exon ligation. However, as discussed above, our structural data are more consistent with a complete dissociation of the EJC in the trans-P complex, and therefore do not support a model in which the EJC remains stably engaged with the RNA. Instead, we have reframed our interpretation to focus on the experimentally supported observation that eIF4A3 is captured in an ADP-bound state in the trans-C* complex. We now discuss this finding in the context of known DEAD-box helicase mechanisms, where ATP hydrolysis is associated with reduced RNA affinity [6,7], and propose that this state may prime the EJC for dissociation upon destabilization of spliceosomal interactions. We have revised the Results sections accordingly to remove speculative mechanistic models and to ensure that all interpretations are directly supported by the structural data (see point 3 above).

5. Page 14, lines 2-6. These descriptions lack a figure to go with it. What do the authors mean by “activated”? Activated for what? Where is the evidence for this “activation” of SDE2-like protein?

   We thank the reviewer for pointing this out and agree that the term “activated” required clarification. By “activated,” we refer specifically to the proteolytic maturation of SDE2 from an inactive precursor into a spliceosome-competent form. SDE2 is synthesized as an inactive precursor containing an N-terminal ubiquitin-like domain fused to the functional C-terminal region via a conserved GGKGG motif. Proteolytic cleavage at the first di-glycine site by the ubiquitin-specific proteases releases the C-terminal fragment, which exposes an N-terminal lysine residue. This processing step is required for SDE2 to associate with spliceosomes and to function in pre-mRNA splicing. Evidence for this “activation” comes from previous studies showing that (i) uncleaved precursor SDE2 fails to incorporate into spliceosomes, (ii) mutations preventing cleavage phenocopy SDE2 loss, and (iii) loss of SDE2 or impairment of its processing leads to defective splicing of specific introns in a subset of pre-mRNAs[9,10] .

   We have revised the text to explicitly describe this step as proteolytic processing required for spliceosomal incorporation (see text below in red). A Supplementary Figure illustrating SDE2-like precursor processing conservation is also added to guide the reader (Supplementary Fig. 17).

"In addition, we identified an unexpected SDE2-like protein that undergoes proteolytic processing via cleavage of its N-terminal ubiquitin-like domain, a mechanism previously described in opisthokonts [53,54] (Supplementary Fig. 17a,b). This processing event generates the mature SDE2-C fragment, which we observe incorporated into the trans-spliceosome catalytic core near the U2/U6 helix I. In this position, SDE2-C contributes to stabilization of the active site, while its C-terminal helix docks onto SYF1 above the U2/U6 helix II in a manner analogous to the human C complex [28] (Supplementary Fig. 17c). Although no direct functional inference can be drawn from our data alone, its conserved positioning suggests a potential role in modulating 3' splice-site selection, similar to SDE2 in metazoans [53] (Fig. 4a, and Supplementary Fig. 13c)."*

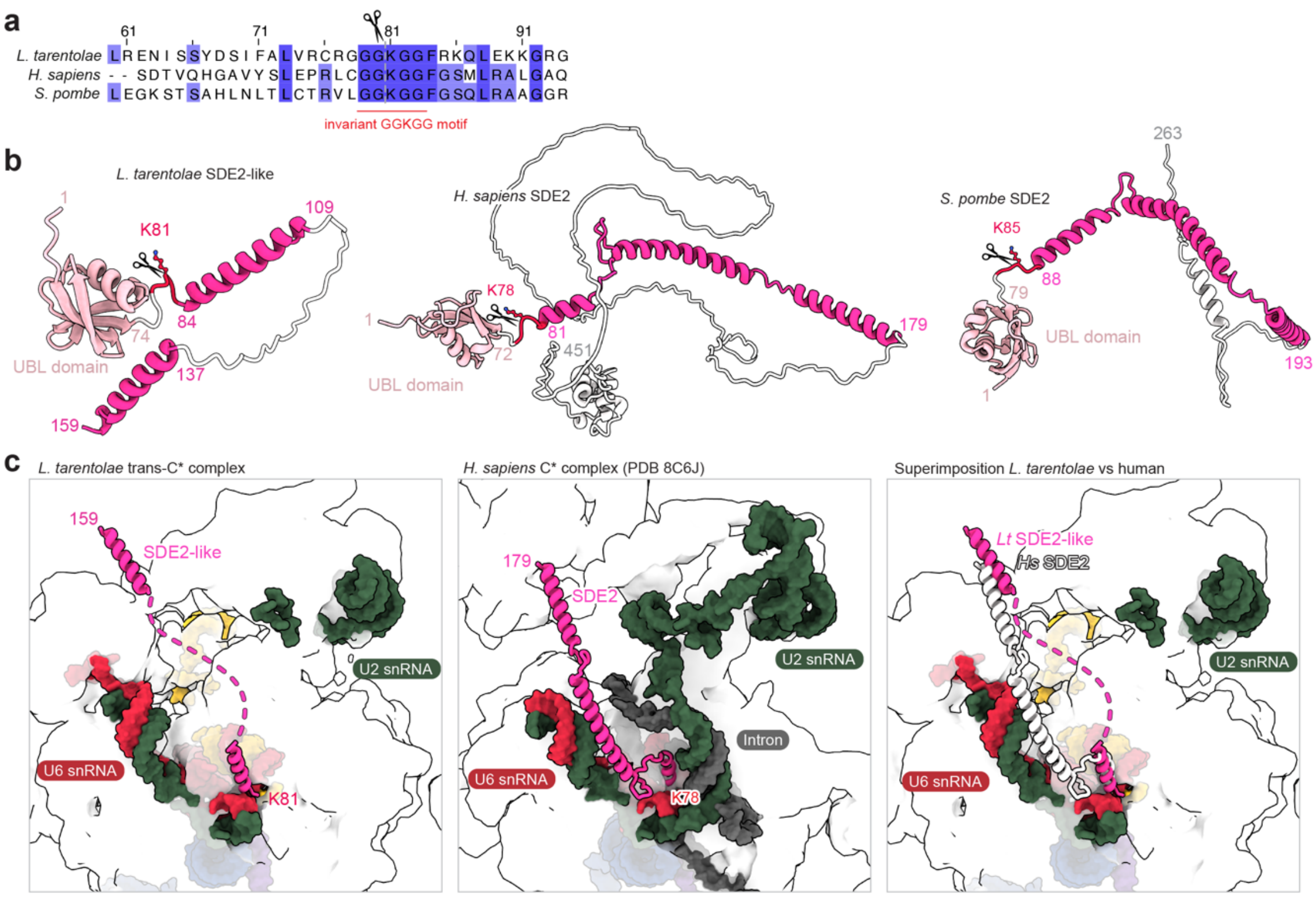

*"**Supplementary Fig. 17**. **Structural analogy of Leishmania tarentolae SDE2-like with SDE2 in opisthokonts**. a Sequence alignment of SDE2 homologs highlighting the conserved proteolytic motif required for maturation. The invariant glycine residues motif (GGKGG) and cleavage site are indicated. b Domain organization and structural representation of SDE2 proteins from different species. The UBL domain (light pink) is removed by proteolytic cleavage, generating the activated SDE2-C fragment (magenta). Residue numbering and key structural elements are indicated. c Localization of the activated SDE2 fragment (magenta) within the (trans-)spliceosome across species. In the L. tarentolae trans-spliceosome, the N-terminus of SDE2 is positioned near U2/U6 helix I, while its C-terminal helix docks onto SYF1 above U2/U6 helix II. Comparison with the human C* complex reveals a conserved spatial arrangement. Superposition highlights the structural analogy despite evolutionary divergence."*

6. Page 14, lines 6-7, line 11. the suggested roles of this SDE2-like protein and LtaPh1601800 are speculative without evidence. Evidence the authors could provide includes expression of mutant proteins (either on top of wild-type protein or in addition in case this could act as a nominant negative mutant) and examine the outcome of RNA splicing via sequencing.

We thank the reviewer for this comment and agree that direct functional validation would be required to definitively establish the roles of the SDE2-like protein and LtaPh1601800 in SL trans-splicing regulation. However, we respectfully note that such experiments (e.g., expression of mutant proteins in *Leishmania* combined with transcriptome-wide splicing analyses) would require extensive additional functional work that is beyond the scope of the present structural study and we believe is well outside a standard revision process.

Importantly, we do not intend to claim demonstrated functions for these factors. Instead, these statements are meant as contextual interpretations, integrating our structural observations with previously established functional data from opisthokonts. Throughout the manuscript, these points are framed as hypotheses or possibilities suggested by structural analogy, rather than experimentally validated conclusions. We would also like to emphasize that this study represents, to our knowledge, the first identification of these factors in trypanosomatids. The observation that proteins previously thought to be restricted to higher eukaryotes have convergent structural counterparts in these divergent eukaryotes provides important evolutionary insight and further supports the relevance of our structural analysis.

To avoid any ambiguity, we have revised the manuscript to further clarify the speculative nature of these interpretations and to explicitly state that functional validation will be required in future studies (see text below in red). These discussion points are included to help position the newly determined structure within the broader framework of the canonical spliceosome and to guide future mechanistic investigations, rather than to assert definitive functional roles.

"Although no direct functional inference can be drawn from our data alone, its conserved positioning suggests a potential role in modulating 3' splice-site selection, similar to SDE2 in metazoans [53] (Fig. 4a, and Supplementary Fig. 13c). Similarly, LtaPh1601800 associates with CRNL1 at a position corresponding to that occupied by FAM192A in humans and Fyv6 in yeast C complexes (Supplementary Fig. 18), both implicated in 3' SS selection [29,31]. While this structural correspondence does not establish a direct functional role, the conserved spatial organization suggests that LtaPh1601800 may fulfill an analogous function in trypanosomatids. Further biochemical and genetic analyses will be required to test this hypothesis. Together, these observations reveal that trypanosomatid spliceosomes combine lineage-specific innovations with unexpected structural analogs of opisthokont factors, pointing to convergent evolution of protein architectures that support catalytic competence."*

7. Page 15, lines 7-9. Similar to the point above, there is no provided evidence for the role of the linker region of PRP22 in regulating PRP22 itself. Either expression of Prp22 and test its RNA binding/helicase activity in vitro with mutation at this linker would be required to support this statement.

We thank the reviewer for this important comment and agree that functional validation is required to support a role for the PRP22 N-terminal linker in autoregulation. Prior to the first submission of this manuscript, we made extensive efforts to purify and biochemically characterize *Leishmania tarentolae* PRP22, including full-length ($PRP22^{FL}$) and N-terminally truncated ($PRP22^{\Delta NTD}$) variants. Despite testing multiple constructs (10His-$PRP22^{FL}$-TwinStrep, 10His-$PRP22^{\Delta NTD}$-TwinStrep, 10His-SUMO-$PRP22^{FL}$-TwinStrep, 10His-SUMO-$PRP22^{\Delta NTD}$-TwinStrep), expression systems (*E. coli* BL21, Rosetta, ArcticExpress), and purification conditions (time of induction, temperature and IPTG concentration) over several months, these attempts were unsuccessful due to persistent solubility and stability issues.

During the revision, we adopted an alternative strategy based on ectopic expression in *L. tarentolae,* which required several weeks of cell line engineering, and finally enabled purification of soluble $PRP22^{FL}$ and $PRP22^{\Delta NTD}$ variants. Yields, however, remained very low (~0.25 mg/12 L for culture for $PRP22^{FL}$ and ~1 mg/12 L for $PRP22^{\Delta NTD}$), highlighting the intrinsic difficulty of working with this protein from *Leishmania* compared to yeast and human PRP22/DHX8 homologs, which have been more readily characterized biochemically[11,12] .

Using the limited material available, we measured ATPase turnover (k_{cat}) and ATP affinity (K_M) for both constructs. These experiments revealed similar K_M but reduced ATPase turnover for $PRP22^{FL}$ compared to the $PRP22^{\Delta NTD}$ variant, consistent with a modulatory effect of the N-terminal region on PRP22 enzymatic activity, and in line with trends reported for human DHX8 [11] (see Supplementary Fig. 20 below)

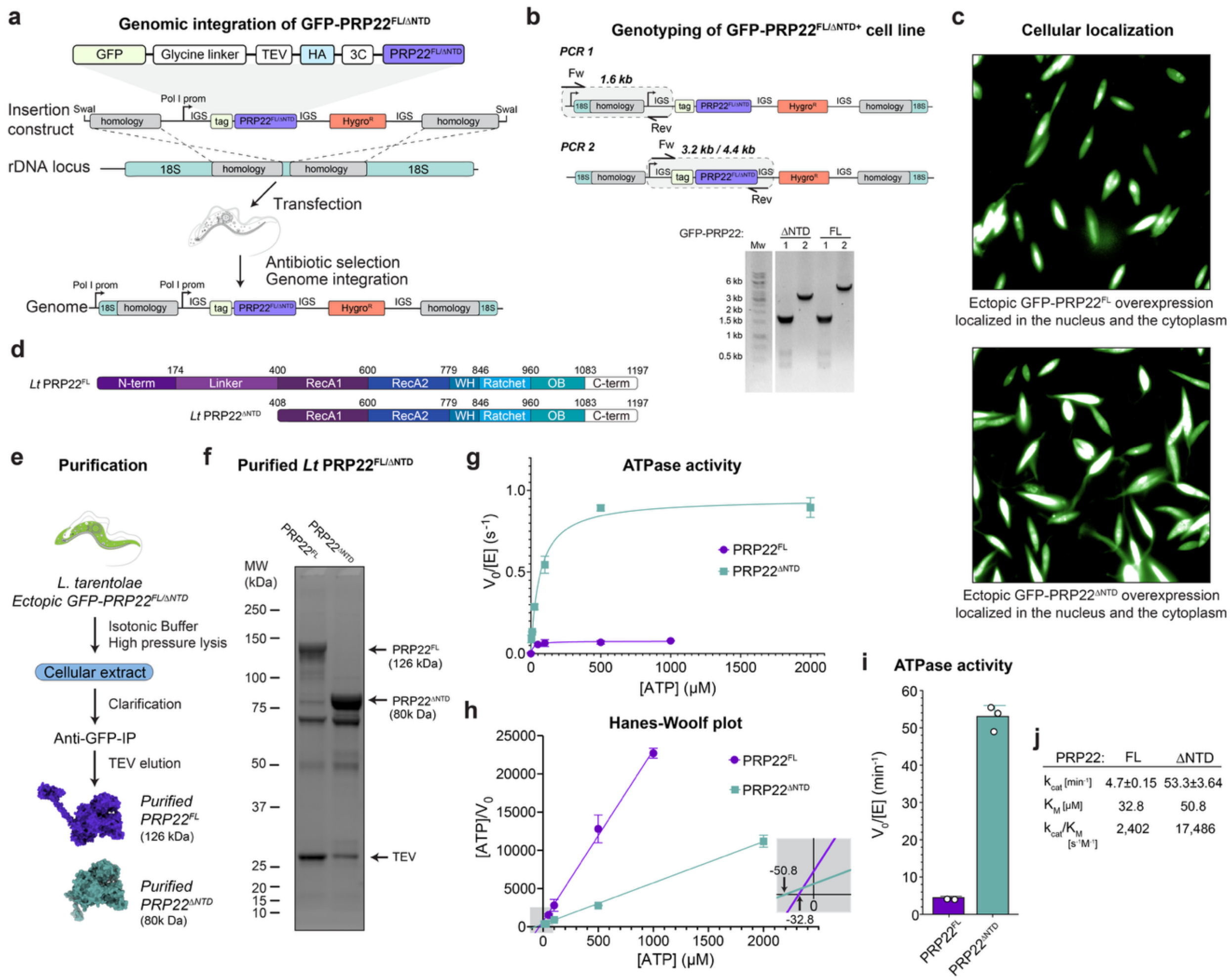

"Supplementary Fig. 20. Expression, purification, and ATPase characterization of PRP22 variants in Leishmania tarentolae.

***a** Schematic of the strategy used to integrate an ectopic copy of GFP-tagged PRP22 (full-length or ΔNTD) into one rDNA locus in L. tarentolae. The construct includes an N-terminal GFP tag followed by a TEV-cleavable glycine linker and a 3C-cleavable HA epitope. **b** Genotyping of GFP–PRP22 cell lines confirming correct genomic integration. Two independent PCR reactions validate insertion of the construct at the rDNA locus for both full-length (FL) and ΔNTD variants. Representative agarose gel analysis is shown. Uncropped gel in Source Data. **c** Fluorescence microscopy images of cells expressing GFP–PRP22^{FL} and GFP–PRP22$^{\Delta NTD}$, showing localization in both the nucleus and cytoplasm. **d** Domain organization of L. tarentolae PRP22 highlighting the N-terminal region, linker, and conserved helicase domains (RecA1, RecA2, WH, ratchet, OB, and C-terminal domains), and the truncation used for the ΔNTD construct. **e** Workflow for purification of PRP22 variants from L. tarentolae cells. **f** SDS–PAGE analysis of purified PRP22^{FL} and PRP22$^{\Delta NTD}$ proteins stained with Coomassie. The positions of PRP22^{FL} (~126 kDa), PRP22$^{\Delta NTD}$ (~80 kDa), and TEV protease are indicated. Uncropped gel in Source Data. **g** Michaelis–Menten analysis of ATPase activity for PRP22^{FL} and PRP22$^{\Delta NTD}$ measured using a continuous coupled assay. Time courses were measured in triplicate for PRP22$^{\Delta NTD}$ (n = 3) and duplicate for PRP22^{FL} (n = 2) due to the limited amount of purified enzyme. Rate constants determined by linear regression. Error bars indicate standard deviations (SD). **h** Hanes–Woolf plot derived from the ATPase measurements shown in panel g. The inset highlights the*

intersection of the linear regressions with the x-axis, corresponding to $-K_M$. Error bars indicate SD. ***i*** *Comparison of ATP turnover rates (v_0/[E]) for $PRP22^{FL}$ and $PRP22^{\Delta NTD}$, measured as initial ATP hydrolysis rates at 1 and 2 mM ATP, respectively, normalized for enzyme concentrations. The error bar represents standard deviation from duplicate or triplicate measurements. Individual measurements are indicated.* ***j*** *Summary of kinetic parameters (k_{cat}, K_M, and k_{cat}/K_M) derived from ATPase assays for both PRP22 variants. Source data are provided as a Source Data file."*

To further investigate the potential regulatory role of the linker at the residue level, we expressed and purified the PRP22 linker region (residues 231–331) fused to MBP, including wild-type and mutant variants targeting residues involved in interactions observed in our structure. We hypothesized that complementation in *trans* of $PRP22^{\Delta NTD}$ with the linker could recapitulate the effect observed with full-length PRP22, and that mutations in the linker might disrupt this modulation. Although we obtained pure and soluble proteins, we were not able to detect any interaction with $PRP22^{\Delta NTD}$ under the tested conditions using biolayer interferometry (1 µM and 10 µM of MBP-linker constructs). Consistently, no effect on ATPase activity was observed at 10 µM of MBP-linker (wild-type or mutant constructs). These results do not invalidate our hypothesis, as we were unable to recapitulate this interaction *in vitro* under the tested conditions, and instead suggest that the interaction observed in the structure may depend on the full spliceosomal context. As these experiments were not informative, we have not included them in the manuscript but provide them below for the reviewers' consideration.

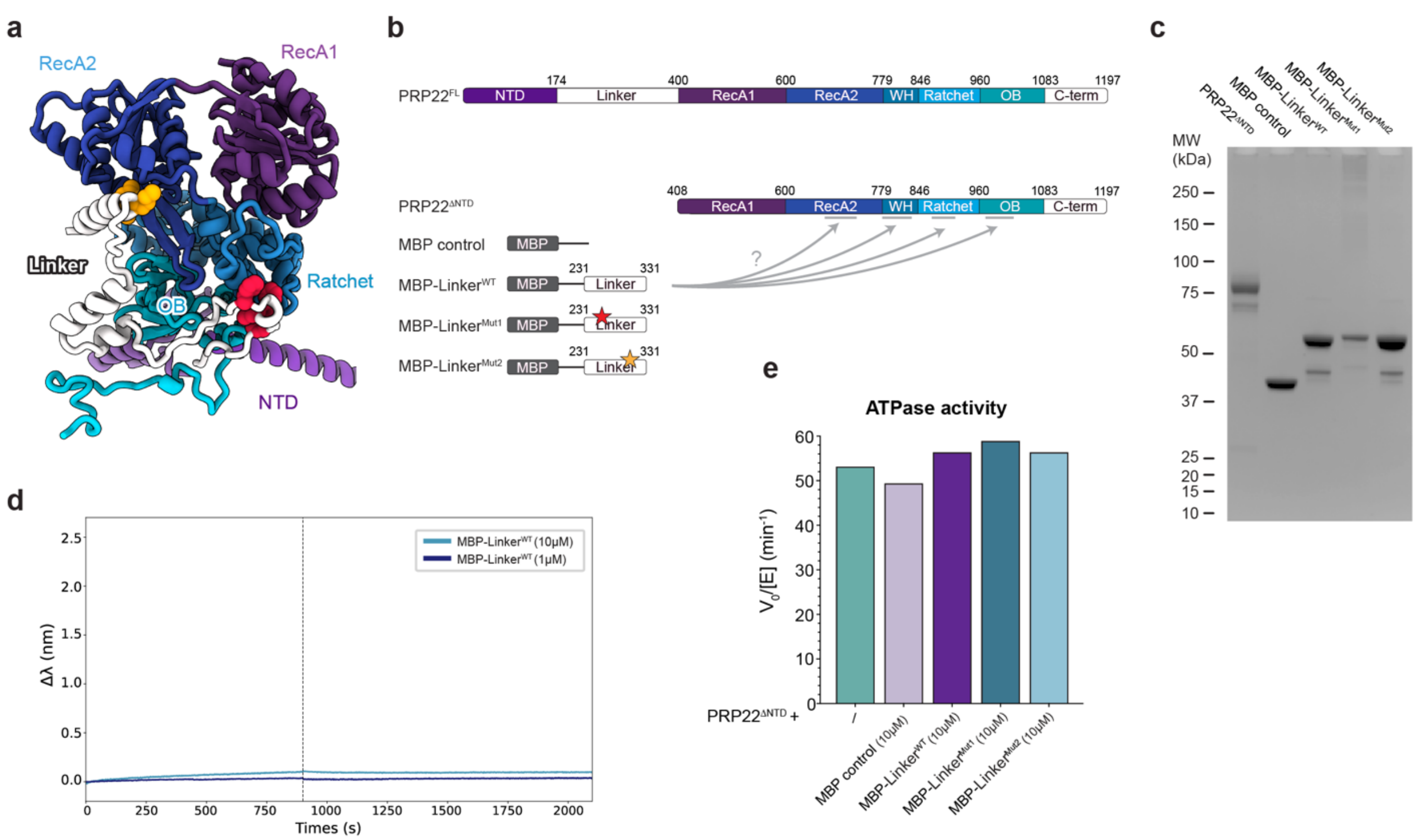

Rebuttal Fig. 1. Functional characterization of the PRP22 linker region.
a Structure of *L. tarentolae* PRP22 in the trans-spliceosome highlighting interactions of the linker region with other domains. Conserved residues of the linker at the interface are shown in orange and red. **b** Schematic representation of the MBP-fused PRP22 linker constructs (residues 231-331), including wild-type and mutant variants (residues shown in a). **c** SDS-PAGE analysis of purified PRP22ΔNTD and MBP-fused linker variants (WT, Mut1, Mut2), as well as an MBP control

lacking the linker. **d** Biolayer interferometry (BLI) analysis of binding between PRP22$^{\Delta NTD}$ and MBP-linker^{WT}. HA-tagged PRP22$^{\Delta NTD}$ was immobilized on AMC (Anti-Mouse Fc Capture) biosensors via a mouse anti-HA monoclonal antibody. AMC biosensors were hydrated in buffer (50 mM HEPES pH 7.8, 150 mM NaCl, 20 mM $MgCl_2$, 1 mM DTT, 0.01% Tween-20, 0.1% BSA) for 10 min prior to measurements. Sensors were loaded with anti-HA antibody (5 µg/mL) for 300s, followed by a 180s wash in buffer. HA-PRP22$^{\Delta NTD}$ was then captured (10 µg/mL, 300s), followed by a 180s baseline in buffer. Association was monitored by dipping sensors into solutions containing 1 or 10 µM MBP-linker^{WT} for 900s, and dissociation was monitored in buffer for 1200s. Curves represent processed association and dissociation signals, corrected by subtracting control measurements performed at the same MBP–linker concentrations in the absence of immobilized HA-PRP22$^{\Delta NTD}$. **e** ATPase activity assays showing ATP turnover rates (v_0/[E]) of PRP22$^{\Delta NTD}$ in the presence of MBP-linker variants. Initial ATP hydrolysis rates were measured at 2 mM ATP and normalized to enzyme concentration.

Overall, our structural data show that the PRP22 linker occupies a position analogous to regulatory G-patch proteins, and our new ATPase measurements indicate that the NTD-linker modulates ATPase activity. These findings support a potential autoregulatory role but the precise mechanistic details remain to be experimentally validated at the residue level. Accordingly, the manuscript has been revised to include the ATPase characterization of both PRP22 variants and to present the autoregulatory role of the linker as an hypothesis that requires further investigation (see text below in red).

"Moreover, we were able to trace the PRP22 linker, which makes extensive contacts with its WH, OB, and Ratchet domains, thereby rigidifying the helicase. Strikingly, these interactions overlap with the binding sites of G-patch proteins that regulate DHX15 activity and RNA affinity in opisthokonts 55 (Supplementary Fig. 19d). Consistently, the NTD linker in the yeast homolog binds to the same region as visualized in recent cryo-EM structures [29], and AlphaFold predictions of full-length human DHX8 similarly position the linker in this region, highlighting a conserved mode of interaction between the linker and the helicase core in PRP22/DHX8. Based on these observations, we hypothesized that the linker region may modulate PRP22 activity, analogous to how G-patch proteins regulate other DExD/H-box helicases. To test this, we attempted to overexpress in E. coli two constructs of the helicase, a full-length protein (PRP22^{FL}) and a truncated variant lacking the NTD and linker (PRP22$^{\Delta NTD}$). However, both variants proved difficult to purify in a soluble form. To overcome this limitation, we generated Leishmania tarentolae cell lines constitutively expressing tagged versions of each construct. This enabled their purification in soluble form, although yields remained low (~0.25 and 1 mg per 12 L culture, respectively). We then compared their ATPase activity using a continuous coupled assay. The K_M values for ATP were similar for PRP22^{FL} (K_M = 27.6 µM) and PRP22$^{\Delta NTD}$ (K_M = 49.6 µM). However, PRP22$^{\Delta NTD}$ displayed a ~10-fold increased ATP turnover rate (k_{cat} = 53.7 ± 3.02 min^{-1}) compared to the full-length enzyme (k_{cat} = 4.7 ± 0.30 min^{-1}), suggesting that the linker may exert an inhibitory effect on ATPase activity. This observation is consistent with reported ATPase activity of the human homolog DHX8, in which the ΔNTD variant exhibits a ~4-fold increase in ATP turnover rate [56], further supporting the idea that the NTD-linker may function as a conserved autoregulatory module. Further mutagenesis targeting residues involved in linker-helicase interactions will be required to dissect this regulatory mechanism at the residue level."

8. Figure S3. 3D classification without alignment, please provide T-value used.

We thank the reviewer for requesting this clarification. We used a standard T-value of 4 for all global classifications performed without alignment and without a mask. This information has been added both to Supplemental Fig. 2 and to the Methods section.

9. Figure S4. The trans-P complex, gold-standard FSC is 2.8 and model-vs-map FSC is 3.3. There is a large gap between these resolutions, suggesting overfitting in the model refinement. The authors should re-refine the structure to make sure this issue is fixed. Also number labels in this figure are very small and difficult to see even. Please enlarge them for visibility.

We thank the reviewer for this comment. In our initial submission, we mistakenly provided the model-to-map FSC calculated using the locally filtered maps, which lack the high-resolution features present in the composite map used for model building and refinement. This explains the apparent discrepancy between the gold-standard map FSC (0.143 criterion) and the model-to-map FSC (0.5 criterion), as the model cannot correlate with high-resolution signal that has been locally filtered out.

We have now included the model-to-map FSC calculated against the composite map, which shows improved agreement at high spatial frequencies. In addition, to rigorously assess potential refinement overfitting, we performed standard half-map cross-validation: models were refined against one half-map using identical refinement parameters used for refinement in the composite map, and model-to-map FSC curves were calculated against both the work (half A) and test (half B) maps [13,14] . The near-complete overlap between FSC-work and FSC-test demonstrates the absence of detectable overfitting during refinement (Supplementary Fig. 5c). We have added the procedure in the Methods section (see text below in red).

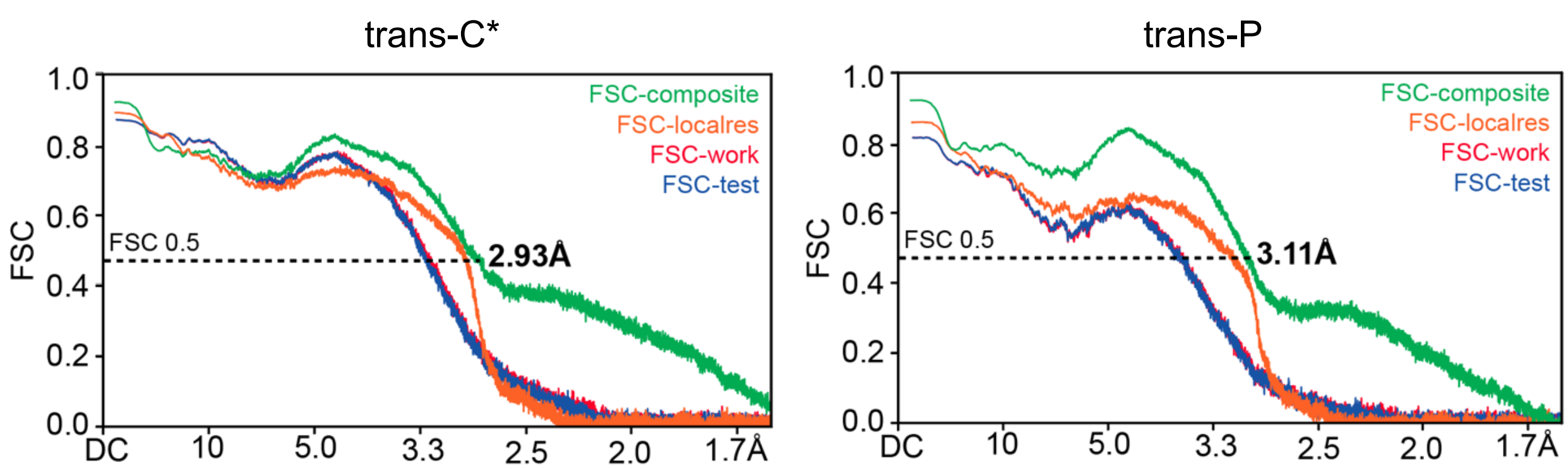

The half-map cross-validation procedure [78,79] was used to assess potential overfitting during refinement. Briefly, the final initial models were refined against one half-map using the same parameters as for the full map, and FSC curves were then calculated separately against half-map A (FSC_work) and half-map B (FSC_test). The near-complete overlap of FSC_work and FSC_test indicates that no detectable overfitting occurred (Supplementary Fig. 4c).

Consistently, model validation statistics reported in Supplementary Table 5 (MolProbity score, CaBLAM outliers, Cβ deviations, EMRinger) all fall within expected ranges for structures at this resolution, further indicating that refinement did not introduce model geometry bias.
Taken together, the FSC cross-validation, geometry metrics, and EMRinger analysis consistently support the conclusion that the model is not overfitted. We therefore consider additional re-refinement unnecessary.

Finally, we have enlarged the number labels in the figure to improve readability, as suggested by the reviewer.

10. Figure S5. The SL SM ring and Brr2 density shown are poor, raising the question of the confidence of the fitting. The authors need to explain what procedure they used for the current fitting of SL Sm ring and BRR2-Prp8 and report confidence scores of the fitting, and maybe present other possible fittings.

The SL Sm ring and BRR2 helicase are highly flexible modules in our dataset, which prevented the reconstruction of high-resolution focused maps despite their clear presence in the sample, as confirmed by mass spectrometry analysis. To nevertheless provide a structurally complete and informative model, these regions were interpreted using lowpass-filtered maps. Rigid-body fitting of BRR2 and the SL Sm ring was performed using the fit_search.py script from the ChimeraX Recipes collection within ChimeraX (https://rbvi.github.io/chimerax-recipes/fit_search/fit_search). The search was carried out with the following parameters: resolution = 25 Å, search = 50, and cutoff = 0.9. For BRR2, among the resulting placements with a cross-correlation above 0.9, we selected the fit (CC=0.912) most consistent with the previously established position and orientation of BRR2 in the human P complex[2] . For the SL Sm ring, among the placements with a cross-correlation above 0.9, we selected the fit (CC=0.953) that best satisfied the expected SL RNA connectivity. The intrinsic flexibility of both BRR2 and the SL Sm ring is thus reflected in their elevated B-factors in the atomic model.

We have now included a detailed description of this fitting procedure, including the fitting scores and selection criteria, in the Methods section (see text below in red).

"BRR2 helicase and the SL Sm ring were rigid-body fitted into their corresponding lowpass–filtered densities using the fit_search.py script from the ChimeraX Recipes collection within ChimeraX [70] (https://rbvi.github.io/chimerax-recipes/fit_search/fit_search.html). The search was performed with the following parameters: resolution 25, search 50, and cutoff 0.9. For BRR2, among the resulting placements with a cross-correlation above 0.9, we selected the fit (0.912) most consistent with the previously established position and orientation of BRR2 in the human P complex [23]. For the SL Sm ring, among the placements with a cross-correlation above 0.9, we selected the fit (0.953) that best satisfied the expected SL RNA connectivity."

Minor comments:

11. Page 2 Line 10: "in silico" should be italic.

We have corrected that.

12. Page 8, line 14. Fig. 1B, C have no comparison with the canonical spliceosome.

We thank the reviewer for raising this point. We have now revised panel b of Fig.1 to allow a direct visual comparison of canonical step II spliceosomes with our step II trans-spliceosomes. White surface represents structural elements of the human step II cis-spliceosome shared with the trans-C* complex, whereas components unique to the trans-C* complex are shown in colored surfaces and components unique to the human step II cis-spliceosome are shown in black surfaces. Trypanosomatid-specific proteins and extensions of conserved proteins are indicated with colored labels, whereas SL trans-splicing-specific components are highlighted using boxed labels. Proteins only found in the human step II spliceosome are indicated with black labels

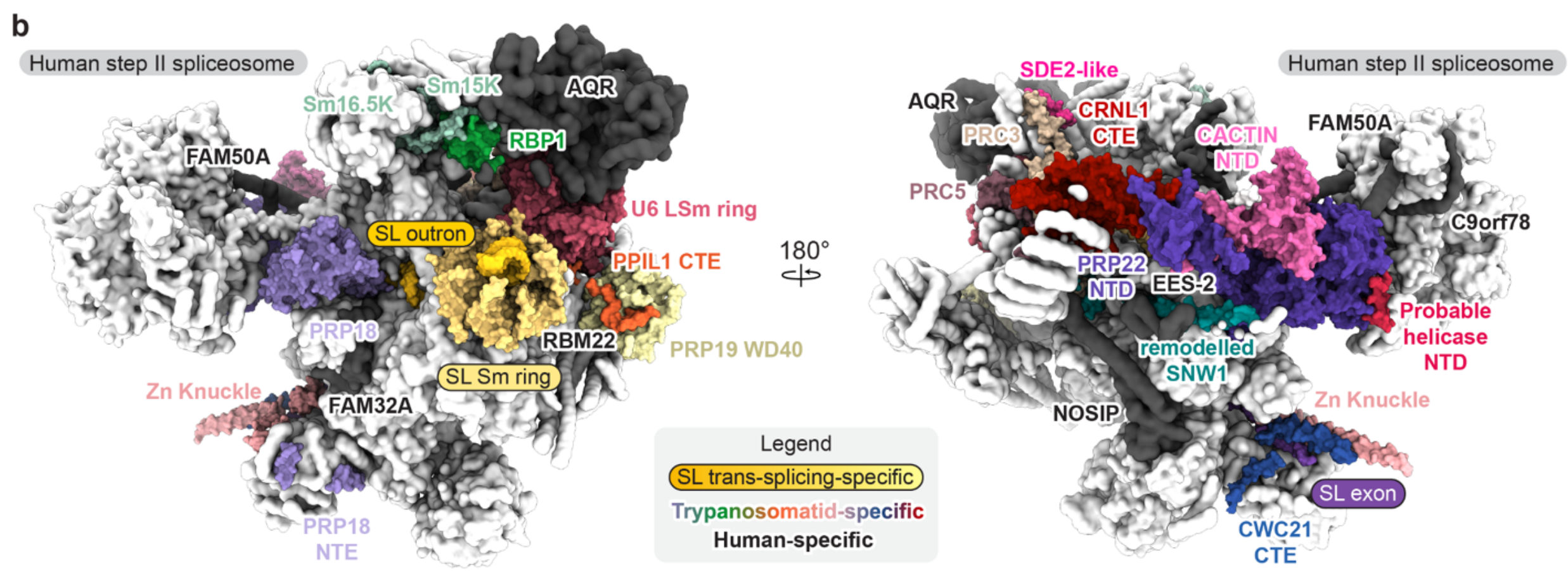

Fig 1. b *Structural comparison between the L. tarentolae trans-C* complex and the human step II cis-spliceosome shown in two views rotated by 180°. White surface represents structural elements of the human step II cis-spliceosome shared with the trans-C* complex, whereas components unique to the trans-C* complex are shown in colored surfaces and components unique to the human step II cis-spliceosome are shown in black surfaces. Trypanosomatid-specific proteins and extensions of conserved proteins are indicated with colored labels, whereas SL trans-splicing-specific components are highlighted using boxed labels. Proteins only found in the human step II spliceosome are indicated with black labels.*

13. Page 9 Line 2: label "loop I" in Figure 2.

We thank the reviewer for this suggestion. We have added the label in Figure 2.

14. 3'-splice site abbreviation is not defined.

We thank the reviewer for finding this omission. We have now defined the 3' splice site in the text before its abbreviation.

15. Page 9, lines 3 and 11, referrals are not specific and very difficult for readers to find which figures refer to which description. Figure S12 seems to be out of order.

We thank the reviewer for this helpful comment. We have made the figure references more specific and renumbered Supplementary Figs. 12 and 13 to restore the correct order.

16. Figure 1B. The legend reads Trypanosome-specific proteins but the figure has proteins that are not trypanosome-specific. It makes it a bit unclear.

We thank the reviewer for this comment. We have now changed panel b to distinguish features that are unique to SL trans-splicing from those that are shared with canonical cis-splicing systems in opisthokonts, as well as trypanosomatid-specific adaptations (either conserved proteins with trypanosomatid-specific N- or C- extensions or lineage-specific novel proteins).

17. Figure 2D, the snRNAs are very transparent. It would be helpful to make them easier to see.

We thank the reviewer for this comment. Initially, we have now increased the contrast of the different snRNA species.

Reviewer #3 (Remarks to the Author)

I co-reviewed this manuscript with one of the reviewers who provided the listed reports. This is part of the Nature Communications initiative to facilitate training in peer review and to provide appropriate recognition for Early Career Researchers who co-review manuscripts.

We thank reviewer #3 for their participation to the reviewing process that allowed us to improve our manuscript.

Rebuttal references

1. Lu, Y., Liang, K. & Zhan, X. Structure of a step II catalytically activated spliceosome from Chlamydomonas reinhardtii. EMBO J. 44, 975–990 (2025).
2. Vorländer, M. K. et al. Mechanism for the initiation of spliceosome disassembly. Nature 632, 443–450 (2024).
3. Dybkov, O. et al. Regulation of 3′ splice site selection after step 1 of splicing by spliceosomal C* proteins. Sci. Adv. 9, eadf1785 (2023).
4. Studer, M. K., Ivanović, L., Weber, M. E., Marti, S. & Jonas, S. Structural basis for DEAH-helicase activation by G-patch proteins. Proc. Natl. Acad. Sci. 117, 7159–7170 (2020).
5. Nielsen, K. H. et al. Mechanism of ATP turnover inhibition in the EJC. RNA 15, 67–75 (2009).
6. Liu, F., Putnam, A. & Jankowsky, E. ATP hydrolysis is required for DEAD-box protein recycling but not for duplex unwinding. Proc. Natl. Acad. Sci. 105, 20209–20214 (2008).
7. Iost, I., Dreyfus, M. & Linder, P. Ded1p, a DEAD-box Protein Required for Translation Initiation in Saccharomyces cerevisiae, Is an RNA Helicase*. J. Biol. Chem. 274, 17677–17683 (1999).
8. Andersen, C. B. F. et al. Structure of the Exon Junction Core Complex with a Trapped DEAD-Box ATPase Bound to RNA. Science 313, 1968–1972 (2006).
9. Thakran, P. et al. Sde2 is an intron-specific pre-mRNA splicing regulator activated by ubiquitin-like processing. EMBO J. 37, 89–101 (2018).
10. Hales, L. T. et al. A Proteolytic Switch: USP5 controls SDE2 function via UBL-directed cleavage. bioRxiv 2025.05.23.655772 (2025) doi:10.1101/2025.05.23.655772.
11. Felisberto-Rodrigues, C. et al. Structural and functional characterisation of human RNA helicase DHX8 provides insights into the mechanism of RNA-stimulated ADP release. Biochem. J. 476, 2521–2543 (2019).
12. Tanaka, N. & Schwer, B. Characterization of the NTPase, RNA-Binding, and RNA Helicase Activities of the DEAH-Box Splicing Factor Prp22 †. Biochemistry 44, 9795–9803 (2005).
13. Brown, A. et al. Tools for macromolecular model building and refinement into electron cryo-microscopy reconstructions. Acta Crystallogr. Sect. D: Biol. Crystallogr. 71, 136–153 (2015).
14. Pintilie, G. & Chiu, W. Validation, analysis and annotation of cryo-EM structures. Acta Crystallogr. Sect. D 77, 1142–1152 (2021).

Point by point response to reviewers' comments

We would like to thank the reviewers for their careful evaluation of our revised manuscript and for their constructive comments. We are pleased that the reviewers found that the revised version addressed the majority of the concerns raised during the initial review process. We have carefully considered the remaining comments (black text) and have revised the manuscript accordingly. Below, we provide a detailed point-by-point response to each concern (blue text).

Reviewer #1 (Remarks to the Author):

The revised manuscript adresses my concerns.

We thank the reviewer for their careful evaluation of the revised manuscript and for their constructive comments throughout the review process. We are pleased that the revisions have addressed their concerns.

Reviewer #2 (Remarks to the Author):

In the revised manuscript and the associated response, the authors have clarified several points that were previously unclear. They addressed most comments about the lack of functional data through removing functional claims rather than functional experiments. Nonetheless, they provided ATPase assays for the "helicase" assignments in response to Points 1 and 7. I found the responses are mostly satisfactory, except for two remaining concerns, particularly in the additional experiments provided, as outlined below.

We thank the reviewer for their careful and thorough assessment of our revised manuscript and for their constructive feedback throughout the review process. We are pleased that the reviewer found that the revisions have clarified the major points raised during the initial round of review. We appreciate the opportunity to further improve our work and have carefully addressed the remaining concerns below.

1. The purity of Prp22 protein (both full-length and truncated) subjected to the ATPase assays is poor with many additional bands unannotated. That of the ATPase in point 1 is better but still there are faint bands at ~100 and 70 KDa. Those contaminants, for example Hsp proteins, can also have ATPase activities themselves, which would affect the results. They also affect protein concentration calculating, leading to incorrect estimation of enzyme kinetic parameters. To avoid such potential problems, the authors should consider purifying the protein further for these assays. This would be the preferred option. Otherwise, they should identify the bands to make sure they themselves do not have ATPase activity or include a negative control, in which they perform similar pulldowns with lysates from cells not integrated with the GFP proteins and subject the elution to ATPase assays. If those are common contaminants, they would come down in the elution yet do not contribute to the ATPase activity observed.

Given that these are the only additional experiments provided in this revised version, I strongly recommend the authors to consider the point below and provide more rigorous controls to ensure the validity of the data.

We thank the reviewer for this comment and for the opportunity to improve the quality of our purified proteins.

A) As noted in our previous revision, the yields of PRP22^{FL} and PRP22$^{\Delta NTD}$ purified from Leishmania cells were relatively low (0.25–1 mg protein from 12 L of culture), which limited our ability to perform conventional second-step purification procedures such as ion-exchange or size-exclusion chromatography. We agree that the preparations used in the previous revision contained additional protein bands and which could potentially possess ATPase activity.

To address this concern, we repeated the purification of both PRP22 variants using the same anti-GFP affinity purification strategy as in the previous revision. Similar yields, purity, and band patterns were obtained (**Rebuttal Fig. 2a,b**). Following the reviewer's suggestion, we included two additional control purifications in which anti-GFP beads were incubated either with wild-type cells lacking the GFP-HA-PRP22 construct or with cells expressing GFP-HA alone, using identical bead-to-lysate and washing conditions. No detectable proteins were recovered from either control purification, indicating that the additional bands observed in the PRP22 preparations are specifically associated with PRP22 rather than resulting from nonspecific binding to the affinity matrix (**Rebuttal Fig. 2b**).

To determine whether these bands corresponded to degradation products or copurifying proteins, we performed western blot analysis using an anti-HA antibody. Because the HA tag is located at the N-terminus of PRP22, this experiment would have allowed us to detect potential C-terminal degradation products. We did not observe any major degradation products. Together, these results suggest that the additional bands do not arise from PRP22 proteolysis (**Rebuttal Fig. 2b-c**). We next excised the major ~70 kDa contaminant band and identified it by in-gel mass spectrometry. Consistent with the reviewer's suggestion, this band corresponded predominantly to HSP70 (**Rebuttal Table 1**).

PRP22$^{\Delta NTD}$

Accession	Description	Sum PEP Score	Coverage	Peptides	PSMs	Unique Peptides	AAs	MW [kDa]
**LtaP26.1170.mRNA-p1**	Heat shock protein 70-related protein	74.732	34.1653666	19	53	19	641	70.6
**LtaP28.2840.mRNA-p1**	Heat shock protein 70	10.717	2.25903614	2	6	2	664	72.4
**LtaP28.1270.mRNA-p1**	Heat shock protein 70-related protein	3.769	5.47112462	4	4	4	658	71.8

PRP22^{FL}

Accession	Description	Sum PEP Score	Coverage	Peptides	PSMs	Unique Peptides	AAs	MW [kDa]
**LtaP26.1170.mRNA-p1**	Heat shock protein 70-related protein	86.346	32.7613105	21	59	21	641	70.6
**LtaP28.2840.mRNA-p1**	Heat shock protein 70	18.75	2.25903614	2	8	2	664	72.4
**LtaP28.1270.mRNA-p1**	Heat shock protein 70-related protein	4.398	7.90273556	5	5	5	658	71.8

Rebuttal Table 1. Proteins identified by in-gel mass spectrometry.

To eliminate HSP70 contamination, we developed an alternative purification strategy based on the N-terminal HA tag using magnetic anti-HA beads (**Rebuttal Fig. 2d**). Bound proteins were subjected to extensive washing with ATP/$MgCl_2$-containing buffer to promote dissociation of HSP70, followed by high-salt washes. PRP22 proteins were subsequently eluted by 3C protease cleavage. This procedure yielded highly purified preparations of both $PRP22^{FL}$ and $PRP22^{\Delta NTD}$, with the only visible additional band corresponding to the 3C protease used for elution (**Rebuttal Fig. 2e**). Protein concentrations were determined by densitometric comparison with a known amount of purified 3C protease. We then repeated the ATPase assays using these highly purified preparations. Both PRP22 variants retained ATPase activity which allowed us to calculate their ATP turnover (Kcat). We found that $PRP22^{\Delta NTD}$ displayed a ~3-fold increased ATP turnover rate (kcat = 43.29 ± 0.79 min^{-1}) compared to the full-length enzyme (kcat = 16.4 ± 0.25 min^{-1}), consistent with our hypothesis that the NTD-linker exert an inhibitory effect on ATPase activity (**Rebuttal Fig. 2f-g**).

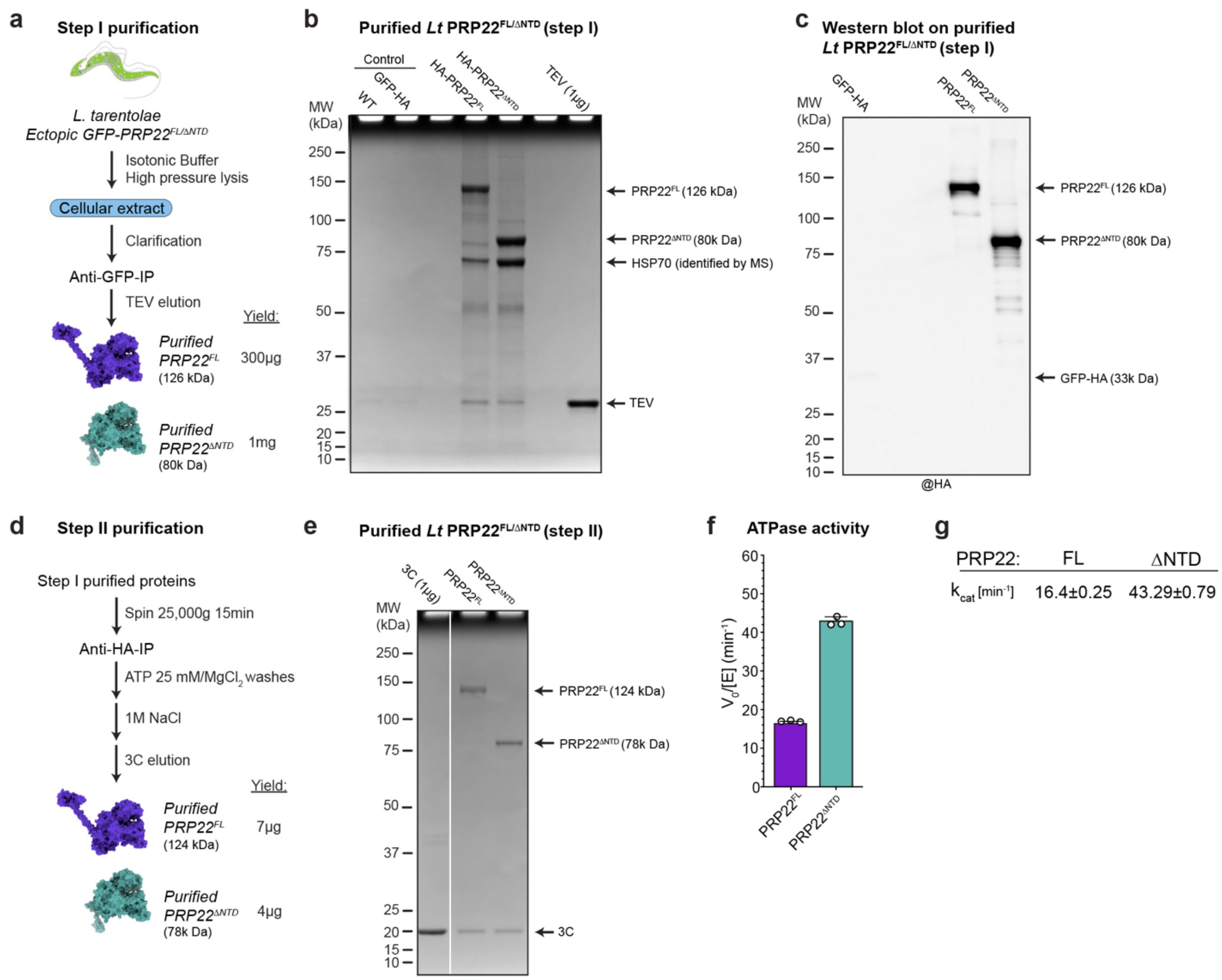

Rebuttal Fig. 2. New 2-step purification of $PRP22^{FL}$ and $PRP22^{\Delta NTD}$ proteins and updated K_{cat} measurements.

a Workflow for the GFP-based purification (step I) of PRP22 variants from L. tarentolae cells. **b** SDS-PAGE analysis of purified $PRP22^{FL}$ and $PRP22^{\Delta NTD}$ proteins obtained from step I, stained with Coomassie. The positions of HA-PRP22FL (~126 kDa), HA-PRP22ΔNTD (~80 kDa), HSP70

(~70 kDa) and TEV protease are indicated. **c** Western blot analysis of step I-purified PRP22FL and PRP22ΔNTD using anti-HA antibody. **d** Workflow for the HA-based purification (step II) of PRP22 variants. **e** SDS–PAGE analysis of purified PRP22FL and PRP22ΔNTD proteins obtained from step II, stained with Coomassie. The positions of PRP22FL (~124 kDa), PRP22ΔNTD (~78 kDa) and 3C protease are indicated. **f** Comparison of ATP turnover rates (v0/[E]) for PRP22FL and PRP22ΔNTD, measured as initial ATP hydrolysis rates at 2 mM ATP and normalized for enzyme concentrations, using a continuous coupled assay. Error bars represents SD from triplicate measurements (n=3). Individual data points are indicated. **g** Summary of kcat values derived from ATPase assays for both PRP22 variants.

B) Regarding the putative ATPase described in point 1 of the first revision, we suspect that the band at ~100 kDa corresponds to SUMO-tagged protein, a small fraction of which likely eluted from the affinity resin during on-column cleavage with Ulp1. Because the protein was readily recovered in the soluble fraction following cell lysis and could be purified at high yield, we consider it unlikely that the ~70 kDa band corresponds to a chaperone such as HSP70.

Nevertheless, to further assess this possibility, we performed an additional purification step consisting of a second Ulp1 cleavage followed by anion-exchange chromatography on a Q-Sepharose column. After sample loading, the column was extensively washed with ATP/$MgCl_2$-containing buffer to promote dissociation of any potential HSP70 contaminants. The protein was subsequently eluted in a single salt step to maximize recovery. The ~100 kDa band was reduced following this additional purification, supporting its assignment as residual SUMO-tagged protein. In contrast, the lower-molecular-weight bands persisted despite extensive ATP/$MgCl_2$ washes, consistent with degradation products rather than HSP proteins or other ATPase chaperones. This interpretation is further supported by the appearance of an additional lower molecular weight band, which is consistent with partial protein degradation during purification (**Rebuttal Fig. 3)**.

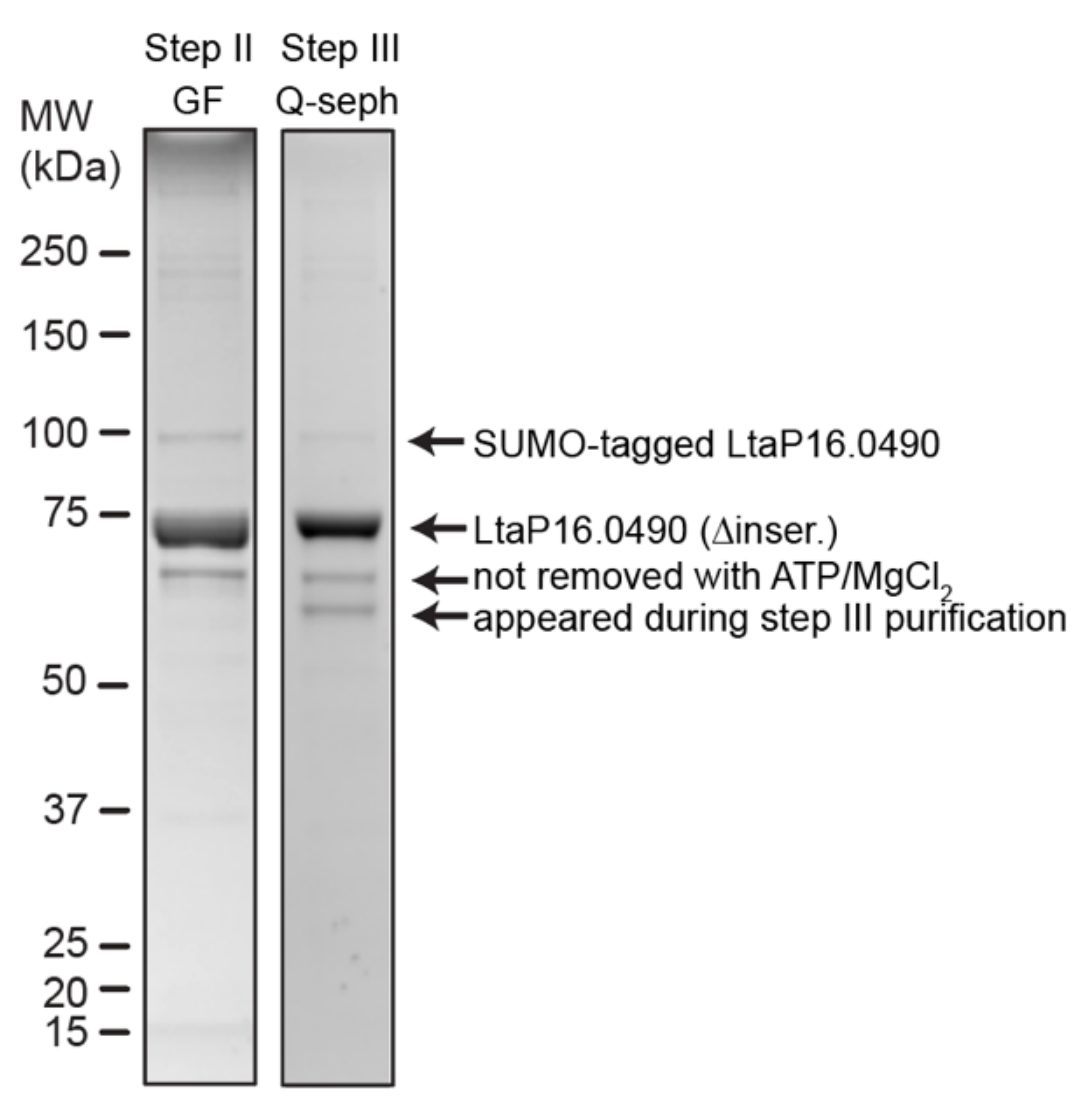

Rebuttal Fig. 3. Comparison of the step II (revision 1) and step III (revision 2) purification of LtaP16.0490.

As the ATPase measurements were performed prior to this additional purification, and our results indicate that the remaining contaminant bands are unlikely to correspond to HSP proteins, we retained the original ATPase activity dataset. However, the enzyme concentration was refined by densitometric analysis, excluding contaminating bands, and k_{cat} values were recalculated accordingly.

We thank the reviewer again for their recommendation to improve our sample quality. We believe that these additional experiments and controls adequately address the concerns regarding protein purity and the origin of the observed ATPase activity. Supplementary Figs. 20 and 22 have been updated accordingly to include the new purifications and ATPase assays.

2. Figure S4. The composite map is not proper for calculating model-to-map FSC. The author should use a refinement map resulting from a cryo-EM refinement for this purpose.

We thank the reviewer for this comment. We agree that model-to-map FSC calculations should be performed against the corresponding cryo-EM refinement maps rather than a composite map. We have therefore updated Supplementary Figure 4 and now report model-to-map FSC curves for both the trans-C* and trans-P states calculated against their respective refinement maps. Additionally, we also provide the standard half-map cross-validations curves. The near-complete overlap between FSC-work and FSC-test demonstrates the absence of detectable overfitting during refinement (see figure below).

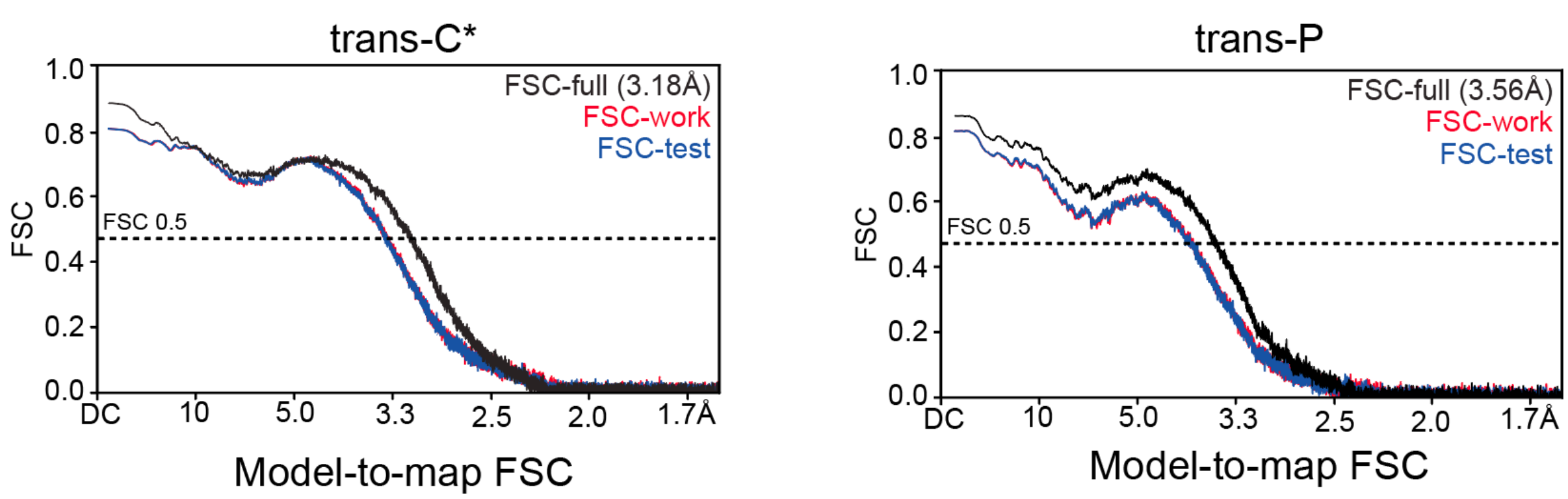

Reviewer #3 (Remarks to the Author):

I co-reviewed this manuscript with one of the reviewers who provided the listed reports. This is part of the Nature Communications initiative to facilitate training in peer review and to provide appropriate recognition for Early Career Researchers who co-review manuscripts.

We thank the reviewers and co-reviewer for their time, careful assessment of our work, and constructive feedback throughout the entire review process.
